# Prediction of Cardiovascular Events in Statin-Treated Stable Coronary Patients of the Treating to New Targets Randomized Controlled Trial by Lipid and Non-Lipid Biomarkers

**DOI:** 10.1371/journal.pone.0114519

**Published:** 2014-12-22

**Authors:** Benoit J. Arsenault, Philip Barter, David A. DeMicco, Weihang Bao, Gregory M. Preston, John C. LaRosa, Scott M. Grundy, Prakash Deedwania, Heiner Greten, Nanette K. Wenger, James Shepherd, David D. Waters, John J. P. Kastelein, the Treating to New Targets (TNT) Investigators

**Affiliations:** 1 Department of Vascular Medicine, Academic Medical Center, University of Amsterdam, Amsterdam, the Netherlands; 2 The Heart Research Institute, Sydney, Australia; 3 Pfizer Inc, New York, NY, United States of America; 4 State University of New York Health Science Center, Brooklyn, NY, United States of America; 5 University of Texas Southwestern Medical Center, Dallas, TX, United States of America; 6 Veterans Affairs Central California Health Care System and University of California San Francisco School of Medicine, San Francisco, CA, United States of America; 7 Universitätsklinikum, Eppendorf, Germany; 8 Emory University School of Medicine, Atlanta, GA, United States of America; 9 University of Glasgow, Glasgow, United Kingdom; 10 San Francisco General Hospital, San Francisco, CA, United States of America; New York University School of Medicine, United States of America

## Abstract

Several plasma non-lipid biomarkers have been shown to predict major cardiovascular events (MCVEs) in population studies. Our objective was to investigate the relationship between lipid and non-lipid biomarkers levels achieved during statin therapy and the incidence of MCVEs in patients with stable coronary heart disease (CHD). We conducted a substudy of the TNT (Treating to New Targets) study, which was a randomized trial that compared the efficacy of high (80 mg) versus low (10 mg) dose atorvastatin for the secondary prevention of CHD. Fasting plasma levels of standard lipids and of 18 non-lipid biomarkers were obtained after an 8-week run-in period on atorvastatin 10 mg in 157 patients who experienced MCVEs during the 4.9 years of study follow-up and in 1349 controls. MCVE was defined as CHD death, nonfatal, non-procedure-related myocardial infarction, resuscitated cardiac arrest, and fatal or nonfatal stroke. After adjusting for age, sex and treatment arm, plasma levels of high-density lipoprotein (HDL) cholesterol, triglycerides, high-sensitivity C-reactive protein (hsCRP), insulin, neopterin, N-terminal pro-brain natriuretic peptide (BNP), lipoprotein(a) [Lp(a)], and the soluble receptor for advanced glycation end products (sRAGE) were predictive of recurrent MCVEs (*P*≤0.02 for each doubling of plasma concentration). However, no significant association was observed between the risk of recurrent MCVEs and plasma levels of low-density lipoprotein cholesterol, adiponectin, cystatin C, lipoprotein-associated phospholipase A2, monocyte chemotactic protein-1, matrix metalloproteinase-9, myeloperoxidase, osteopontin, soluble CD40 ligand, soluble intercellular adhesion molecule-1, or soluble vascular cell adhesion molecule-1. After further adjustment for diabetes, hypertension, smoking, and BMI, the relationship between hsCRP, insulin and MCVE were no longer significant, while the relationship between Lp(a), neopterin, NT-proBNP and sRAGE and MCVE remained statistically significant. In conclusion, in patients with CHD treated with atorvastatin, plasma levels of Lp(a), neopterin, NT-proBNP, and sRAGE are associated with the risk of recurrent MCVEs.

**Trial Registration:**

ClinicalTrials.gov NCT00327691.

## Introduction

There has been much recent interest in the ability of non-lipid biomarkers associated with systemic inflammation, oxidative stress, tissue remodelling and/or insulin resistance to predict adverse cardiovascular outcomes and to identify individuals at high risk of future coronary heart disease (CHD) events and stroke [1], [2]. The concentration of one of these, high-sensitivity C-reactive protein (hsCRP), predicts future cardiovascular events in apparently healthy individuals and in subjects treated with statins [3]–[5]. The results of these studies have been used to support the argument that the concentration of non-lipid biomarkers such as hsCRP should be included in algorithms designed to predict cardiovascular outcomes and to measure the efficacy of statin treatment [6]. However, there are inconsistencies, with some studies finding that levels of non-lipid biomarkers have minimal predictive power beyond of established CHD risk factors [7]–[9]. We further address this issue by investigating how the concentrations of plasma lipids and non-lipid biomarkers relate to cardiovascular events in the Treating to New Targets (TNT) study.

## Methods

In 2007, the investigators of the TNT trial launched an initiative aimed at identifying blood-derived biomarkers that predicted cardiovascular risk. We had previously published the entire content of this manuscript in 2011 [10]. However, discrepancies between the anonymized and the clinical database were noticed in late 2012. After having realized that the results of this investigation had impacted the results of the manuscript, we have immediately retracted it (see notice of retraction for further details [11]). As mentioned in the retraction notice, since the error was discovered, we have created a new anonymized clinical and biomarker database by restoring the original set of anonymized identifiers. We have reanalyzed all the data according to our original study plans and hereby present our results.

### Study design

**Figure 1:**
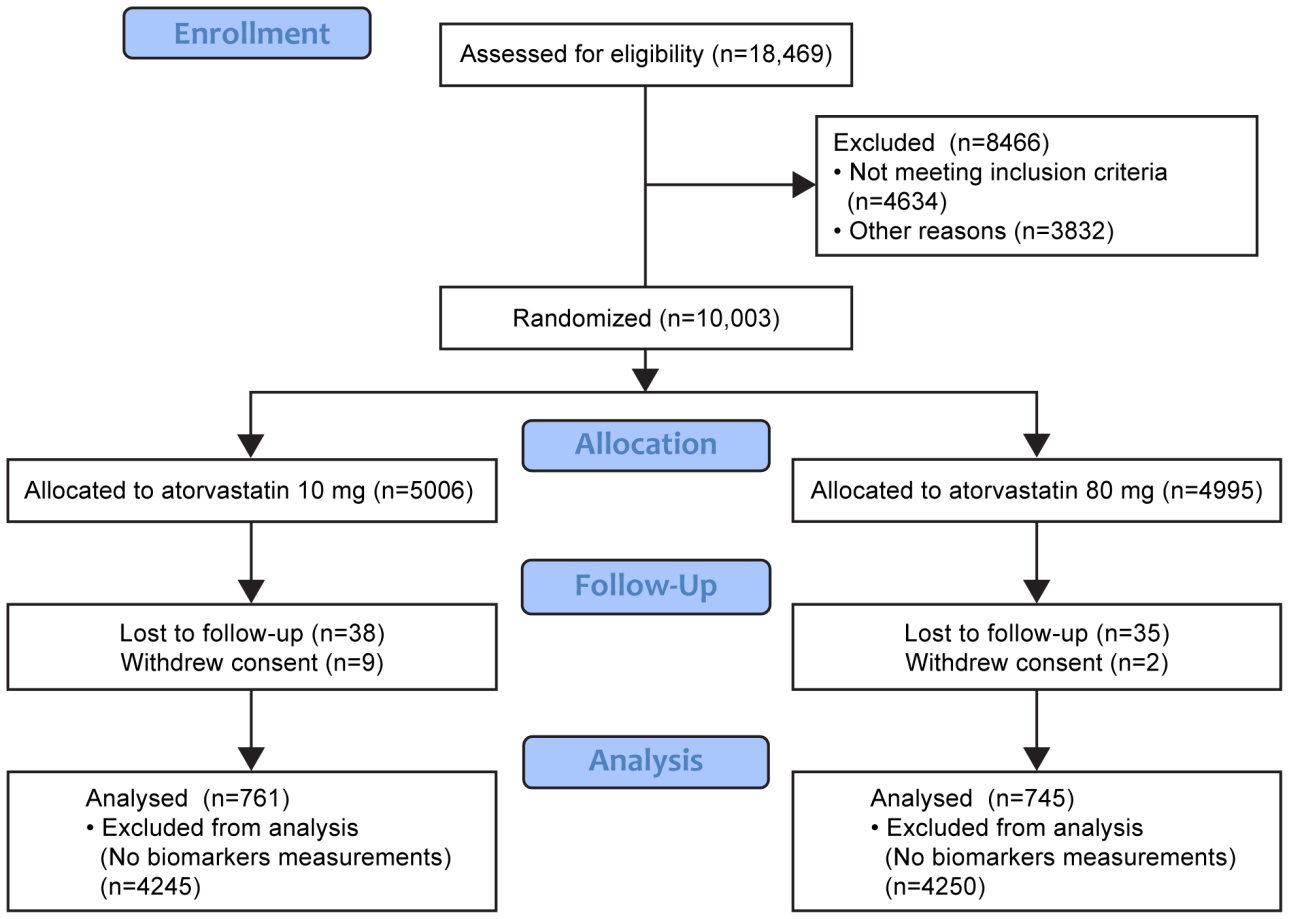
Flowchart describing the study cohort and the patients included in this study.

The study protocol and outcome measures for the TNT study have been published previously [12]. The supporting CONSORT checklist for this trial is available as S1 Checklist, S1 Protocol. Patients were recruited between July 1998 and December 1999. In brief, patients with clinically manifest CHD commenced 8 weeks of open-label treatment with atorvastatin 10 mg/day. After this run-in period, 10,001 patients with low-density lipoprotein (LDL) cholesterol levels <3.4 mmol/L (<130 mg/dL) were randomized in a double-blind design to therapy with either 10 mg or 80 mg of atorvastatin per day. Patients were followed for a median of 4.9 years. Only patients from whom informed consent was obtained for measuring non-lipid biomarkers (in addition to that originally collected for the primary study) were selected for this substudy. Biomarkers were measured in a random sample that included 1506 patients of whom 157 experienced a MCVE (Fig. 1). The primary endpoint was the time to the first occurrence of a major CV event (MCVE), defined as CHD death (n = 24), nonfatal, non-procedure-related myocardial infarction (n = 86), resuscitated cardiac arrest (n = 5), and fatal or nonfatal stroke (n = 42). Biomarker concentrations were measured in fasting plasma samples collected at the time of randomization (after the 8-week atorvastatin 10 mg run-in period) and again 1 year after randomization. The association between biomarkers and MCVEs were evaluated for biomarkers measured at baseline (157 events) and at 1 year after randomization (133 events). The number of samples ranged from 1491 to 1506 at randomization, and ranged from 1429 to 1469 at year 1. All patients gave written informed consent, and the study was approved by the local research ethics committee or institutional review board at each center. The CONSORT 2010 checklist of information to include when reporting a randomized trial was submitted at the same time of the manuscript.

### Biomarker analyses

The biomarkers analyzed in the present study and their biologic relevance are listed in Table 1. Biomarkers were chosen for analysis on the basis of having previously demonstrated potential utility in improving cardiovascular disease risk prediction in human clinical and population studies. Plasma levels of high molecular weight (HMW) adiponectin and total adiponectin were measured by an enzyme-linked immunosorbent assay (ELISA, Millipore, Inc.); the HMW/total adiponectin ratio was computed by dividing HMW adiponectin by total adiponectin levels. Myeloperoxidase (MPO), matrix metalloproteinase-9 (MMP-9), soluble intercellular adhesion molecule-1 (sICAM-1), and soluble vascular cell adhesion molecule-1 (sVCAM-1) were measured with the human CVD Panel 1 LINCOplex kit (Millipore, Inc.). High-sensitivity C-Reactive protein (hsCRP) levels were measured with the human CVD Panel 2 LINCOplex kit (Millipore, Inc.). Plasma levels of monocyte chemoattractant protein-1 (MCP-1) and soluble CD40 ligand (sCD40L) were measured with the human cytokine LINCOplex kit (Millipore, Inc.). Cystatin C and receptor for advanced glycation ends products (RAGE) levels were measured by ELISA (R&D Systems). Insulin levels were measured by a radioimmunoassay (Linco RIA, Millipore, Inc.). Lipoprotein-associated phospholipase A2 (Lp-PLA2) levels were measured with the PLAC test (diaDEXUS, Inc.). Lipoprotein (a) [Lp(a)] levels were measured by a latex immonoturbidimetric assay (Wako Chemical Inc.). Neopterin levels were measured by ELISA (Alpha Diagnostics, Inc.). NT-proBNP levels were measured by ELISA (Alpco Diagnostics, Inc.). The human osteopontin quantikine ELISA kit was used to measure osteopontin levels (R&D Systems, Inc.). All biomarkers were measured at Millipore BioPharma Services Laboratory, St. Charles, MO, USA. We have performed representative quality controls among random samples across the study. Inter-assay coefficients of variation ranged from 6.6% (Lp-PLA2) to 24.8% (sCD40L).

**Table 1:** Biomarkers studied in TNT.

Pathophysiologic Role	Biomarkers Analyzed
Systemic inflammation	High-sensitivity C-reactive protein (hsCRP)
Macrophage Recruitment/Activity	Monocyte chemotactic protein-1 (MCP-1); neopterin; soluble intercellular adhesion molecule-1 (sICAM-1); soluble vascular cell adhesion molecule-1 (sVCAM-1)
Oxidative stress	Myeloperoxidase (MPO); lipoprotein-associated phospholipase A2 (Lp-PLA2)
Tissue remodeling	Matrix metalloproteinase-9 (MMP-9); osteopontin
Platelet/Thrombosis	Soluble CD40 ligand (sCD40L); lipoprotein(a) [Lp(a)]
Insulin resistance	Insulin; adiponectin; high molecular weight (HMW) adiponectin; HMW/total adiponectin (ratio); soluble receptor for advanced glycation end-products (sRAGE)
Congestive heart Failure	N-terminal fragment of pro-B-type natriuretic peptide (NT-pro-BNP)
Kidney function	Cystatin C

### Statistical methods

Patient characteristics at randomization were provided by treatment group in the main study and in this substudy. Statistical comparison used a Chi-square test for categorical variables, and a Wilcoxon rank-sum test for continuous variables. Similarly, characteristics of substudy patients at time of randomization were compared between those who did and those who did not experience a CV event during the study follow-up. Changes in biomarkers were tested with a signed-rank test, and compared between treatment groups with a Wilcoxon rank-sum test. The association between on-treatment lipids and biomarker levels (at time of randomization and at 1 year) and primary endpoint was assessed in Cox proportional hazard analyses after adjustment for age, gender and treatment effect, using time to primary end point as the dependent variable for all patients and for patients within each treatment group. Independent variables included the log_2_ transformed biomarker level. Study treatment-by-baseline biomarker interactions were assessed separately in the same model to test if the effect of biomarkers differed between atorvastatin- and placebo-treated subjects.

## Results

### Patient population

Screening, enrolment and biomarker study population is presented in Fig. 1. Characteristics of patients in this substudy were similar to those in the total TNT population (Table 2). Characteristics of patients in the biomarker subgroup who experienced a MCVE and those who did not are also shown in Table 2 for the classic CHD-risk factors, and in Table 3 for the lipid and non-lipid biomarkers. At this time of randomization all participants had been taking atorvastatin at a dose of 10 mg per day for at least 8 weeks.

**Table 2:** Patients' characteristics at time of randomization.^a^

Characteristic	Main Study		Biomarker Substudy					
	By treatment		All patients (n = 1506)	By treatment		By event		
	Atv 10 mg (n = 5006)	Atv 80 mg (n = 4995)		Atv 10 mg (n = 761)	Atv 80 mg (n = 745)	With event (n = 157)	Without event (n = 1349)	*P*-value^b^
Age (y)	60.9 (8.8)	61.2 (8.8)	61.5 (8.7)	61.3 (8.5)	61.8 (9.0)	63.0 (8.4)	61.4 (8.8)	0.0226
Male (%)	80.8	81.2	79.9	80.0	80.0	84.7	79.2	0.1061
Risk factor (%)								
Current smoker	13.4	13.4	12.9	14.6	10.7	19.1	11.9	0.0248
Hypertension	54.4	53.9	57.2	58.6	55.8	70.1	55.7	0.0006
Diabetes	15.0	15.0	16.6	16.8	16.35	28.0	15.3	0.0001
Lipids (mg/dl)								
LDL cholesterol	98 (18)	98 (17)	98 (18)	98 (18)	97 (18)	98 (19)	97 (18)	0.8259
Total cholesterol	175 (24)	175 (24)	175 (24)	175 (23)	175 (25)	175 (23)	175 (24)	0.7358
Triglycerides	151 (72)	151 (70)	155 (71)	154 (68)	155 (74)	168 (86)	153 (69)	0.0963
HDL cholesterol	47 (11)	47 (11)	47 (11)	46 (11)	47 (11)	44 (10)	47 (11)	0.0001

Continuous variables are mean (standard deviation). Atv is atorvastatin, LDL is low-density lipoprotein, and HDL is high-density lipoprotein.

a At the time of randomization, all participants had been on 10 mg atorvastatin for at least 8 weeks.

b *P*-value for patients who experienced an event versus those who did not in the biomarker subgroup.

**Table 3:** Non-lipid biomarker levels at time of randomization.^a^

Non-Lipid Biomarker	Biomarker Substudy by Treatment		Biomarker Substudy by Event		
	Atv 10 mg (n = 761)	Atv 80 mg (n = 745)	With event (n = 157)	Without event (n = 1349)	*P*-value^b^
Adiponectin (ng/ml)	6575 (4791, 9228)	6587 (4798, 9388)	6559 (4680, 8911)	6588 (4799, 9331)	0.8158
HMW adiponectin (ng/ml)	1922 (1155, 3015)	1991 (1213, 3226)	2057 (1394, 3193)	1927 (1166, 3100)	0.1056
HMW/total adiponectin	0.290 (0.217, 0.361)	0.297 (0.233, 0.370)	0.307 (0.243, 0.400)	0.291 (0.223, 0.362)	0.0106
hsCRP (mg/l)	1.7 (0.8, 4.0)	1.7 (0.8, 3.7)	2.1 (1.0, 4.9)	1.7 (0.8, 3.8)	0.0380
Cystatin C (ng/ml)	781.8 (681.5, 924.4)	781.4 (670.7, 912.9)	841.9 (712.5, 1011.8)	774.4 (672.8, 910.8)	0.0003
Insulin (uU/ml)	13.0 (9.0, 18.0)	12.0 (9.0, 16.0)	13.5 (9.0, 18.0)	12.0 (9.0, 17.0)	0.1191
Lp-PLA2 (ng/ml)	326.0 (262.0, 389.0)	322.0 (262.0, 388.0)	329.0 (248.0, 393.0)	324.0 (264.0, 388.0)	0.9368
Lipoprotein(a) (mg/ml)	15.0 (5.0, 42.0)	14.0 (5.0, 37.0)	20.0 (5.0, 48.0)	14.0 (5.0, 38.5)	0.0538
MCP-1 (pg/ml)	98.0 (75.0, 129.0)	102.0 (75.0, 134.0)	103.0 (76.0, 135.0)	99.0 (75.0, 130.0)	0.2660
MMP-9 (pg/ml)	44,456 (29,669, 67,498)	43,379 (30,353, 66,107)	44,295 (29,537, 70,479)	43,945 (30,066, 66,456)	0.9259
MPO (pg/ml)	22,893 (10,783, 61,995)	20,478 (10,156, 50,182)	23,650 (11,004, 62,380)	21,246 (10,324, 54,022)	0.3939
Neopterin (ng/ml)	2.9 (2.3, 3.6)	2.9 (2.3, 3.6)	3.2 (2.4, 4.3)	2.9 (2.3, 3.5)	0.0006
Nt-pro-BNP (fmol/ml)	496.9 (397.9, 637.9)	523.0 (412.4, 659.9)	611.4 (453.8, 810.2)	501.4 (400.1, 637.4)	<0.0001
Osteopontin (ng/ml)	46.3 (32.8, 59.3)	47.0 (32.1, 59.7)	50.0 (37.0, 59.8)	46.3 (32.3, 59.4)	0.0602
sRAGE (pg/ml)	1339 (1010, 1797)	1319 (1022, 1791)	1450 (1066, 2006)	1318 (1009, 1768)	0.0063
sCD40L (pg/ml)	3930 (1878, 9513)	4069 (1881, 9539)	3865 (1905, 9190)	4013 (1870, 9615)	0.6184
sICAM-1 (ng/ml)	145.0 (110.0, 185.0)	142.0 (106.0, 185.0)	148.0 (110.0, 209.0)	143.0 (107.0, 183.0)	0.0775
sVCAM-1 (ng/ml)	1034 (858, 1272)	1066 (889, 1256)	1050 (813, 1262)	1050 (879, 1266)	0.4363

Values are median (range). Atv is atorvastatin, HMW is high molecular weight, hsCRP is high-sensitivity C-reactive protein, Lp-PLA2 is lipoprotein-associated phospholipase A2, MCP-1 is monocyte chemotactic protein-1, MMP-9 is matrix metalloproteinase-9, MPO is myeloperoxidase, Nt-pro-BNP is N-terminal fragment of pro-B-type natriuretic peptide, sRAGE is soluble receptor for advanced glycation end-products, sCD40L is soluble CD40 ligand, sICAM-1 is soluble intercellular adhesion molecule, and sVCAM-1 is soluble vascular cell adhesion molecule-1.

a At the time of randomization, all participants had been on 10 mg atorvastatin for at least 8 weeks.

b *P*-value for patients who experienced an event versus those who did not in the biomarker subgroup.

### Relationships of MCVEs to biomarker levels measured at time of randomization

Table 4 shows the relationship between standard lipids and non-lipid biomarkers and risk of MCVE after adjusting for age, gender and treatment arm (pooled group) using Cox proportional hazards. In this analysis of the combined 10 mg and 80 mg atorvastatin groups, the concentrations of traditional lipids high-density lipoprotein (HDL) cholesterol and triglycerides measured at randomization were predictive of MCVEs. LDL cholesterol levels at randomization were not associated with MCVEs. Plasma levels of hsCRP, insulin, neopterin, NT-proBNP, Lp(a), and sRAGE were predictive of recurrent MCVEs (*P*≤0.02 for each doubling of plasma concentration). However, plasma levels of adiponectin, cystatin C, Lp-PLA2, MCP-1, MMP-9, MPO, osteopontin, sCD40L, sICAM-1, and sVCAM-1 were not associated with the risk of recurrent MCVEs. Among the biomarkers that showed a positive association with MCVE in the combined group, after further adjustment for diabetes, hypertension, smoking, and BMI, the relationship between CRP (p = 0.22), insulin (p = 0.80) and MCVE were no longer significant, while the relationship between Lp(a) (p = 0.004), neopterin, (p = 0.0003), NT-proBNP (p<0.0001) and sRAGE (p = 0.0005) and MCVE remained statistically significant. For biomarkers that were predictive of recurrent MCVEs, we confirmed in a Supremum test that the proportional hazards assumption was not violated in the analysis models.

**Table 4:** Relationships of MCVEs to biomarker levels measured at time of randomization.^a^

Lipid or Non-Lipid Biomarker	All Patients			Atorvastatin 10 mg			Atorvastatin 80 mg		
	HR^b^	95% CI	*P*-value	HR^b^	95% CI	*P*-value	HR^b^	95% CI	*P*-value
Lipid biomarker									
LDL cholesterol	1.14	0.63, 2.07	0.6608	1.28	0.58, 2.82	0.5407	1.004	0.41, 2.49	0.9928
HDL cholesterol	0.34	0.19, 0.58	0.0001	0.41	0.20, 0.83	0.0130	0.25	0.10, 0.60	0.0019
Triglycerides	1.40	1.07, 1.81	0.0125	1.66	1.17, 2.35	0.0042	1.09	0.72, 1.64	0.6897
Non-lipid biomarker									
Adiponectin	1.04	0.86, 1.26	0.6615	1.11	0.86, 1.44	0.4280	0.94	0.69, 1.29	0.7057
HMW adiponectin	1.10	0.94, 1.29	0.2247	1.19	0.97, 1.46	0.0972	0.99	0.79, 1.25	0.9428
HMW/total adiponectin	1.10	0.94, 1.29	0.2144	1.12	0.94, 1.33	0.1941	1.06	0.76, 1.46	0.7479
hsCRP	1.10	1.01, 1.19	0.0219	1.04	0.94, 1.15	0.4754	1.21	1.06, 1.38	0.0045
Cystatin C	1.20	0.92, 1.56	0.1814	1.39	0.92, 2.09	0.1162	1.05	0.76, 1.46	0.7605
Insulin	1.25	1.04, 1.50	0.0154	1.28	1.01, 1.63	0.0444	1.22	0.92, 1.62	0.1673
Lp-PLA2	0.94	0.64, 1.38	0.7396	0.86	0.52, 1.43	0.5669	1.07	0.59, 1.95	0.8318
Lipoprotein(a)	1.13	1.03, 1.25	0.0133	1.16	1.02, 1.32	0.0247	1.10	0.94, 1.28	0.2504
MCP-1	1.14	0.94, 1.39	0.1874	1.04	0.80, 1.36	0.7514	1.31	0.95, 1.79	0.1002
MMP-9	1.01	0.86, 1.18	0.9269	1.10	0.91, 1.34	0.3282	0.84	0.62, 1.12	0.2268
MPO	1.02	0.93, 1.11	0.6709	1.08	0.97, 1.21	0.1466	0.92	0.79, 1.06	0.2336
Neopterin	1.56	1.25, 1.94	<0.0001	1.58	1.21, 2.05	0.0008	1.54	1.03, 2.31	0.0342
Nt-proBNP	2.03	1.53, 2.68	<0.0001	1.83	1.26, 2.65	0.0015	2.31	1.52, 3.50	<0.0001
Osteopontin	1.07	0.92, 1.24	0.3961	1.03	0.85, 1.24	0.8038	1.13	0.89, 1.43	0.3327
sRAGE	1.54	1.20, 1.98	0.0008	1.63	1.17, 2.26	0.0040	1.42	0.96, 2.08	0.0788
sCD40L	0.97	0.89, 1.06	0.4663	0.93	0.83, 1.04	0.2160	1.02	0.89, 1.16	0.7932
sICAM-1	1.24	0.98, 1.57	0.0733	1.18	0.88, 1.58	0.2719	1.35	0.91, 2.00	0.1322
sVCAM-1	0.87	0.67, 1.12	0.2662	0.99	0.70, 1.41	0.9746	0.58	0.34, 0.97	0.0370

HR is hazard ratio, CI is confidence interval, LDL is low-density lipoprotein, HDL is high-density lipoprotein, HMW is high molecular weight, hsCRP is high-sensitivity C-reactive protein, Lp-PLA2 is lipoprotein-associated phospholipase A2, MCP-1 is monocyte chemotactic protein-1, MMP-9 is matrix metalloproteinase-9, MPO is myeloperoxidase, Nt-pro-BNP is N-terminal fragment of pro-B-type natriuretic peptide, sRAGE is soluble receptor for advanced glycation end-products, sCD40L is soluble CD40 ligand, sICAM-1 is soluble intercellular adhesion molecule, and sVCAM-1 is soluble vascular cell adhesion molecule-1.

a At the time of randomization, all participants had been on 10 mg atorvastatin for at least 8 weeks.

b Hazard ratio associated with doubling the concentration and adjusting for age, gender, and treatment effect. Treatment interaction by individual biomarker is not significant for all biomarkers analyzed.

### Effect of treatment on biomarker levels

**Figure 2:**
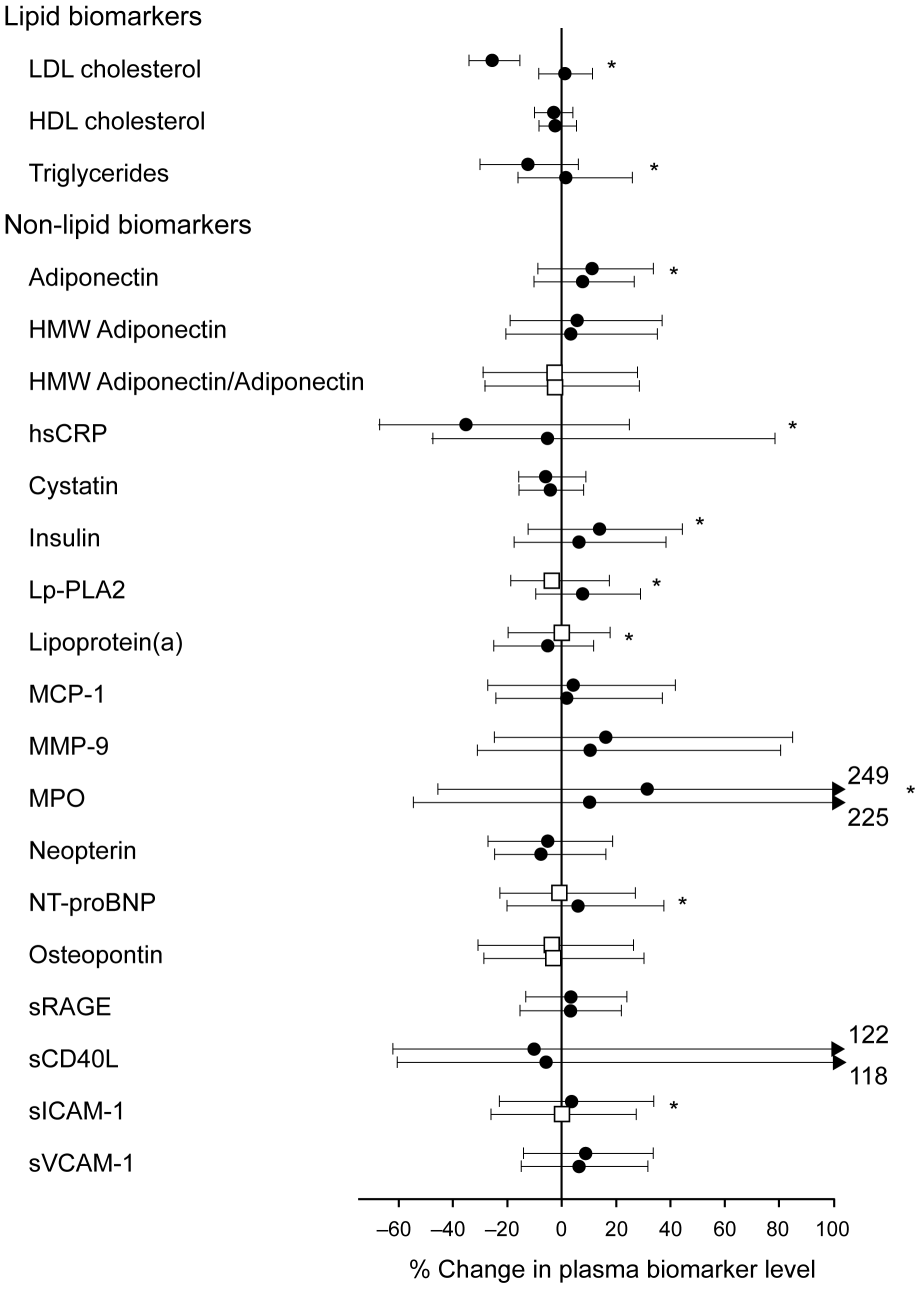
Median (interquartile range) percent change in lipid and non-lipid biomarkers levels after 1 year of treatment with atorvastatin 10 mg or 80 mg. The asterisks indicate significant differences in the percentage change between 10 mg and 80 mg atorvastatin groups (2-sided Wilcoxon rank-sum test). For each biomarker, the top bar represents changes with 80 mg atorvastatin and the bottom bar represents changes with 10 mg atorvastatin. Black circles represent significant changes from baseline to 1-year (*P*<0.05 from signed rank test) and white squares represent non-significant changes. LDL is low-density lipoprotein, HDL is high-density lipoprotein, HMW is high molecular weight, hsCRP is high-sensitivity C-Reactive Protein, Lp-PLA2 is Lipoprotein-associated Phospholipase A2, MCP-1 is Monocyte Chemotactic Protein-1, MMP-9 is Matrix Metalloproteinase-9, MPO is myeloperoxidase, Nt-pro-BNP is N-terminal fragment of pro-B-type natriuretic peptide, sRAGE is soluble receptor for advanced glycation end-products, sCD40L is soluble CD40 ligand, sICAM-1 is soluble intercellular adhesion molecule and sVCAM-1 is soluble vascular cell adhesion molecule-1.

Significant changes from baseline in the levels of all lipids and some non-lipid biomarkers were observed after 1 year in both the 10 and 80 mg atorvastatin groups (Fig. 2). There were some significant differences between the changes observed in the two treatment groups. For instance, atorvastatin 80 mg induced more pronounced reductions in plasma levels of hsCRP, Lp-PLA2, and NT-proBNP; and induced more significant increases in plasma levels of adiponectin, insulin, Lp(a), and MPO.

### Relationships between biomarker levels measured after 1 year of treatment and MCVEs

After 1 year of treatment with atorvastatin, levels of HDL cholesterol and triglycerides remained significant predictors of subsequent MCVEs in the combined groups (Table 5) after adjusting for age, gender and treatment arm (pooled group). As for non-lipid biomarkers, both Lp(a) and NT-proBNP were still associated with the risk of recurrent cardiovascular events. The association between hsCRP, insulin, neopterin, and sRAGE measured after 1 year were no longer associated with the risk of MCVEs. We also found a significant treatment arm by biomarker level interaction for the prediction of MCVEs for Lp(a). Among the biomarkers that showed a positive association with MCVE in the combined group, after further adjustment for diabetes, hypertension, smoking, and BMI, the relationship between Lp(a) (p = 0.007), and NT-proBNP (p = 0.004) and MCVE remained statistically significant.

**Table 5:** Relationships of MCVEs to biomarker levels measured after 1 year of treatment.^a^

Lipid or Non-Lipid Biomarker	All Patients			Atorvastatin 10 mg			Atorvastatin 80 mg		
	HR^b^	95% CI	*P*-value	HR^b^	95% CI	*P*-value	HR^b^	95% CI	*P*-value
Lipid biomarker									
LDL cholesterol	1.18	0.73, 1.91	0.4992	1.50	0.72, 3.11	0.2805	0.995	0.52, 1.91	0.9870
HDL cholesterol	0.38	0.22, 0.66	0.0007	0.39	0.19, 0.82	0.0131	0.37	0.16, 0.89	0.0202
Triglycerides	1.47	1.16, 1.86	0.0014	1.57	1.16, 2.15	0.0041	1.33	0.92, 1.93	0.1338
Non-lipid biomarker									
Adiponectin	1.16	0.88, 1.52	0.2899	1.29	0.90, 1.85	0.1721	1.01	0.68, 1.51	0.9613
HMW adiponectin	1.17	0.97, 1.41	0.1026	1.19	0.93, 1.51	0.1704	1.14	0.85, 1.52	0.3772
HMW/total adiponectin	1.20	0.93, 1.55	0.1542	1.17	0.82, 1.66	0.3888	1.23	0.87, 1.76	0.2418
hsCRP	1.02	0.91, 1.13	0.7803	1.00	0.87, 1.16	0.9777	1.03	0.88, 1.21	0.7211
Cystatin C	1.27	0.94, 1.72	0.1259	1.30	0.87, 1.95	0.2034	1.21	0.76, 1.93	0.4218
Insulin	1.21	0.96, 1.52	0.1090	1.32	0.97, 1.79	0.0798	1.09	0.76, 1.55	0.6446
Lp-PLA2	0.89	0.56, 1.41	0.6209	0.92	0.49, 1.71	0.7828	0.88	0.44, 1.75	0.7056
Lipoprotein(a)	1.17	1.04, 1.33	0.0106^c^	1.34	1.12, 1.60	0.0013	1.01	0.85, 1.20	0.8964
MCP-1	1.19	0.94, 1.50	0.1574	1.20	0.91, 1.60	0.2018	1.14	0.76, 1.73	0.5255
MMP-9	1.08	0.90, 1.30	0.4194	1.11	0.88, 1.41	0.3676	1.02	0.76, 1.38	0.8918
MPO	1.05	0.94, 1.17	0.3888	1.09	0.95, 1.24	0.2361	0.99	0.84, 1.18	0.9273
Neopterin	1.18	0.86, 1.63	0.3112	1.15	0.76, 1.74	0.5148	1.21	0.72, 2.02	0.4693
Nt-proBNP	1.70	1.20, 2.41	0.0028	1.40	0.89, 2.18	0.1435	2.21	1.29, 3.77	0.0037
Osteopontin	0.995	0.84, 1.18	0.9498	0.91	0.74, 1.11	0.3364	1.18	0.86, 1.60	0.3065
sRAGE	1.17	0.86, 1.58	0.3137	1.23	0.83, 1.82	0.3054	1.09	0.68, 1.74	0.7240
sCD40L	0.97	0.88, 1.06	0.4786	0.98	0.86, 1.11	0.6912	0.95	0.82, 1.11	0.5335
sICAM-1	0.996	0.74, 1.33	0.9803	1.01	0.68, 1.51	0.9609	0.979	0.64, 1.50	0.9243
sVCAM-1	0.79	0.56, 1.12	0.1848	0.79	0.47, 1.35	0.3884	0.78	0.48, 1.26	0.3090

HR is hazard ratio, CI is confidence interval, LDL is low-density lipoprotein, HDL is high-density lipoprotein, HMW is high molecular weight, hsCRP is high-sensitivity C-reactive protein, Lp-PLA2 is lipoprotein-associated phospholipase A2, MCP-1 is monocyte chemotactic protein-1, MMP-9 is matrix metalloproteinase-9, MPO is myeloperoxidase, Nt-pro-BNP is N-terminal fragment of pro-B-type natriuretic peptide, sRAGE is soluble receptor for advanced glycation end-products, sCD40L is soluble CD40 ligand, sICAM-1 is soluble intercellular adhesion molecule, and sVCAM-1 is soluble vascular cell adhesion molecule-1.

a Individuals who experienced a MCVE within 1 year of follow-up (n = 34) were not included in the present analyses.

b Hazard ratio associated with doubling the concentration and adjusting for age, gender, and treatment effect.

c *P* = 0.05 for treatment by biomarker interaction.

## Discussion

This substudy of the TNT trial was designed to investigate the ability of a number of lipid and non-lipid biomarkers to predict MVCEs in stable, statin-treated CHD patients. Our results suggest that plasma levels of Lp(a), neopterin, NT-proBNP, and sRAGE are significantly associated with the risk of recurrent MCVEs. The relationship between NT-proBNP and Lp(a) with cardiovascular risk was also observed 1 year following randomization. In contrast, plasma levels of LDL cholesterol, adiponectin, cystatin C, hsCRP, insulin, Lp-PLA2, MCP-1, MMP-9, myeloperoxidase, osteopontin, sCD40 ligand, sICAM-1, and sVCAM-1 were not associated with the risk of recurrent MCVEs.

Several studies have shown that statins reduce plasma levels of markers associated with systemic inflammation, oxidative stress, tissue remodeling, and/or insulin resistance [4], [5], [13]. In our study, the uptitration to atorvastatin 80 mg was associated with a 35.2% decrease in hsCRP levels, while patients who remained on the 10 mg dose had a decrease in plasma hsCRP levels of 5.2%. This finding is very similar to observations in the Reversal of Atherosclerosis with Aggressive Lipid Lowering (REVERSAL) trial in which, 80 mg atorvastatin provided a 36.4% decrease in hsCRP levels compared with 5.2% for patients treated with 40 mg pravastatin [14]. This finding is also in line with other trials performed in patients with CHD, such as the Comparative Atorvastatin Pleiotropic (CAP) effects study and the Pravastatin or Atorvastatin Evaluation and Infection Therapy–Thrombolysis in Myocardial Infarction 22 (PROVE IT–TIMI 22) study, in which, a dose-response effect of statin on hsCRP levels was observed (80 mg atorvastatin versus 40 mg pravastatin in PROVE IT-TIMI 22 and 80 mg atorvastatin versus 10 mg atorvastatin in CAP) [4], [15]. As for the predictive value of hsCRP, our results are similar to those of PROVE IT-TIMI 22, Aggrastat-to-Zocor (A to Z), and the Justification for the Use of Statin in Primary Prevention: an Intervention Trial Evaluating Rosuvastatin (JUPITER) trials in which, plasma levels of CRP did predict CVD outcomes in statin-treated patients with acute coronary syndrome and in primary prevention [4], [16], [17]. It should be noted however that hsCRP levels were positively associated with MCVE risk after adjusting for age, sex and treatment arm, but not after further adjustment of diabetes, BMI, smoking and hypertension.

The strongest effect was observed with NT-proBNP for which a doubling of plasma concentration was associated with a doubling of cardiovascular risk. The considerable impact of plasma NT-proBNP levels on cardiovascular disease risk has been observed in a meta-analysis of the Emerging Risk Factor Collaboration published in 2009 [18]. Interestingly, this meta-analysis included the results of previously published statin trials such as the Heart Protection Study (HPS) [19], the Long-Term Intervention with Pravastatin in Ischaemic Disease (LIPID) [20] and PROVE IT-TIMI 22 [21], which have all shown that plasma NT-proBNP levels strongly predicted the risk of cardiovascular events.

Our results suggest that one of the biomarkers that is most influenced by atorvastatin treatment is MPO, in which, plasma levels increased by 10.4 and 31.6%, respectively for the 10 and 80 mg atorvastatin groups. Such an increase in MPO levels upon statin therapy has already been observed by Meuwese et al. [22] in a sample of patients with heterozygous familial hypercholesterolemia treated with either atorvastatin 80 mg or simvastatin 40 mg. Also, consistent with previously published reports [23], increasing the dose of atorvastatin to 80 mg was associated with a small, but significant rise in plasma insulin levels.

Of all the lipid and non-lipid biomarkers studied, only Lp(a) showed a modest interaction with the atorvastatin dose in predicting outcomes after 1 year of atorvastatin therapy as all other biomarkers did not interact with statin dose in prediction outcomes after one year. Interestingly, genetic variations at the *LPA* locus were recently identified by Deshmukh [24] et al. as the second most important loci influencing LDL cholesterol reductions (after *APOE*) upon atorvastatin therapy. The HPS investigators [25] have also reported a significant impact of the same genetic variant in *LPA* associated with LDL response following simvastatin therapy. The positive association between plasma Lp(a) levels and the risk of events observed in the present study is supported by a significant amount of reports suggesting a role for both Lp(a) levels and *LPA* genotyped in predicting cardiovascular risk [26], [27]. Finally, two less studied biomarkers did show a positive association with the risk of cardiovascular events in our study: neopterin and sRAGE. Neopterin is a marker of monocyte activation that has been shown to predict cardiovascular events in PROVE IT-TIMI 22 [28]. sRAGE has been shown to be associated with cardiovascular risk in atorvastatin-treated patients with type 2 diabetes of the Collaborative Atorvastatin Diabetes Study (CARDS) [29]. Although increasing the dose of atorvastatin had little or no effect on these biomarkers, their potential role in cardiovascular risk stratification in patients treated with statins requires further investigation.

### Study limitations

Several aspects of our study design may have had an impact on our results and conclusions. For instance, although all the biomarkers that we have highlighted as significant predictors of cardiovascular risk have been flagged in previous statin trials, our conclusions are based on relatively few incident cardiovascular events. This likely explains the absence of a relationship between LDL cholesterol levels and cardiovascular events given that LDL cholesterol levels did predict MCVEs in the entire study population [10]. It should also be mentioned that at baseline, all participants had completed a run-in phase of 8 weeks on atorvastatin 10 mg, and therefore had lower LDL cholesterol levels to begin with. The TNT trial did not include a placebo group, as all participants received active treatments. It was thus, not possible to make comparisons with untreated patients. In this study, levels of lipid and non-lipid biomarkers were measured at randomization, when all subjects had already been on 10 mg atorvastatin treatment for at least 8 weeks. It was therefore not possible to investigate the relationship between biomarkers level off-statin and cardiovascular risk. We have studied the relationship between 18 biomarkers and risk of events at two time-points. The high number of statistical tests performed may increase the odds of reporting false positives. Had we applied a Bonferroni correction (α = 0.05/18 biomarkers), the threshold value for a significant p-value should have been 0.003, which would have ruled out the significant association between baseline levels of hsCRP, insulin and Lp(a) and CV events as well as the association between 1-year levels of Lp(a) and CV events. A minority of assay were also characterized by slightly higher interassay coefficients, as mentioned in the Methods section. A survival bias also cannot be excluded when investigating the association between 1-year biomarkers levels and MCVE risk.

In conclusion, our results suggest that on top of traditional lipid parameters, several emerging cardiovascular disease risk factors such as NT-pro-BNP, Lp(a), neopterin, and sRAGE are indeed associated with the risk of cardiovascular events. Whether or not therapies aiming at reducing the plasma levels of these biomarkers could be beneficial in terms of cardiovascular risk reduction warrants further investigation.

## Supporting Information

S1 Checklist CONSORT checklist.
(DOC)

S1 Protocol (PDF)
