# Supplementary material for: Prediction of Cardiovascular Events in Statin-Treated Stable Coronary Patients of the Treating to New Targets Randomized Controlled Trial by Lipid and Non-Lipid Biomarkers
Source: PLoS One. 2014 Dec 22;9(12):e114519. doi: 10.1371/journal.pone.0114519 (PMC4273994; doi:10.1371/journal.pone.0114519)
Supplement: S1 Protocol — (PDF) [file pone.0114519.s002.pdf]

PROTOCOL SIGNATURE FORM

Final Approval

Clinical* Signature: [redacted] — 13-Jun-03 Date

[redacted]r, Dr PH
Executive Director, Clinical Development

Biostatistics & Reporting (B & R) Signature: [redacted] — 17-Jun-03 Date

[redacted]
Associate Director, Statistics

Protocol ID: 981-117/258-102

Title: THE EFFECT OF LDL-CHOLESTEROL LOWERING BEYOND CURRENTLY RECOMMENDED MINIMUM TARGETS ON CORONARY HEART DISEASE (CHD) RECURRENCE IN PATIENTS WITH PRE-EXISTING CHD

Version Date: 13 June 2003

- ☐ Initial Protocol
- ☑ **Global Amendment**
- ☐ Country Amendment
- ☐ Center Amendment

I affirm that the protocol has been appropriately developed and reviewed and is ready for approval.

Author Signature: [redacted] — 13 June 2003 Date

[redacted]
Associate Director

**** Global Clinical Leader (GCL), Early Therapeutic Area Leader (ETAL), Clinical Technology, etc***

Upon completion, file to Trial Master File

<letter/sentence/paragraph deleted> **Pfizer**

CLINICAL PROTOCOL PHASE IV

<letter/sentence/paragraph deleted>
PFIZER PROTOCOL #***981-117/***258-102

TITLE: THE EFFECT OF LDL-CHOLESTEROL LOWERING BEYOND CURRENTLY RECOMMENDED MINIMUM TARGETS ON CORONARY HEART DISEASE (CHD) RECURRENCE IN PATIENTS WITH PRE-EXISTING CHD

AMENDMENT DATE:	October 6, 1998	(Amendment 1)
	January 15, 1999	(Amendment 2)
	June 4, 1999	(Amendment 3)
	June 13, 2003	***(Amendment 4)***

CONFIDENTIAL

<u>June 13, 2003 – Amendment 4</u>

PFIZER PROTOCOL #981-117/*258-102* SYNOPSIS

TITLE THE EFFECT OF LDL-CHOLESTEROL LOWERING BEYOND CURRENTLY RECOMMENDED MINIMUM TARGETS ON CORONARY HEART DISEASE (CHD) RECURRENCE IN PATIENTS WITH PRE-EXISTING CHD

INDICATION Coronary heart disease (CHD)

<u>June 13, 2003 – Amendment 4</u>
OBJECTIVES To assess the ***cardiovascular*** event reduction efficacy and safety of low-density lipoprotein cholesterol (LDL-C) lowering to achieve LDL-C targets beyond currently recommended minimums when compared to only achieving the minimum targets.

PATIENT POPULATION Patients with pre-existing CHD (prior myocardial infarction, angina or coronary revascularization) who have an LDL-C 130-250 mg/dl (3.4-6.5 mmol/l) and triglyceride level ≤ 600 mg/dl (6.8 mmol/l).

<u>June 13, 2003 – Amendment 4</u>
SUMMARY OF STUDY This is a double-blind, randomized, parallel-group study of the ***occurrence*** of ***cardiovascular events*** in patients whose LDL-C will be lowered on average to approximately 100 mg/dl (2.6 mmol/l) compared with those whose LDL-C is approximately 25% lower. Patients will have clinically evident CHD. The study has 3 periods: (1) a screening visit to determine a patient's initial eligibility to participate in the study, (2) an open-label atorvastatin 10 mg run-in period whose primary purpose is to achieve an LDL-C <130 mg/dl (3.4 mmol/l) and determine baseline study parameters, and (3) a randomized double-blind treatment period (atorvastatin 10 vs. 80 mg once daily) expected to require an average ***5.5*** years duration ***<letter, sentence, paragraph deleted>*** until the target number of ***events*** is achieved, ***<letter, sentence, paragraph deleted>.***

TREATMENT Open-label atorvastatin 10 mg once daily for 8 weeks during the run-in period. Double-blind randomized treatment to achieve target LDL-C levels using either atorvastatin 10 mg once daily or atorvastatin 80 mg once daily during the double-blind period.

<u>June 13, 2003 – Amendment 4</u>
PRIMARY EFFICACY PARAMETERS The occurrence of the primary clinical endpoint of major ***cardiovascular*** events (defined as ***<letter, sentence, paragraph deleted>*** CHD death,***<letter, sentence, paragraph deleted>*** non-fatal myocardial infarction, ***resuscitated cardiac arrest, or stroke [fatal or non-fatal]).***

SECONDARY EFFICACY PARAMETERS The occurrence of the following clinical events: ***major coronary event (CHD death, non-fatal myocardial infarction or resuscitated cardiac arrest);*** any ***coronary*** event (***major coronary event*** or CABG, PTCA, other revascularization procedure, ***procedure-related myocardial infarction***, or documented angina); ***cerebrovascular event (fatal or non-fatal stroke, TIA)***; peripheral vascular disease; hospitalization with primary diagnosis of CHF; any cardiovascular event (any of the above); and all-cause mortality.

SAFETY PARAMETERS Safety will be assessed by electrocardiograms, physical examinations, and by monitoring clinical laboratory parameters, vital signs, and the frequency and intensity of adverse events.

ECONOMIC EVALUATIONS All hospital admissions, length of stay and revascularization procedures will be collected. Data from this trial will be combined with market-specific data on costs to complete net cost analyses and cost-effectiveness analyses.

STATISTICAL RATIONALE AND ANALYSIS A statistical rationale for the number of patients in the study is provided in the protocol.

TOTAL PLANNED SAMPLE SIZE A minimum of 35 patients at each of 150-250 centers for 8,600 randomized patients.

ANTICIPATED START / COMPLETION DATES 2Q 1998/ 2Q 2005

STUDY COUNTRY LOCATION North America, Europe, South Africa, and Australia

June 13, 2003 – Amendment 4
DESCRIPTION OF MEDICATIONS

Pfizer Drug Code	Generic Name	Strength and Dosage Form	Therapeutic Classification
981/258	atorvastatin	40-mg tablets	lipid regulator
981/258	atorvastatin	10-mg tablets	lipid regulator

PROTOCOL FOR ***<letter/sentence/paragraph deleted>*** PFIZER ***<letter/sentence/paragraphdeleted>***

TITLE: The Effect Of LDL-Cholesterol Lowering Beyond Currently Recommended Minimum Targets On Coronary Heart Disease (CHD) Recurrence In Patients With Pre-Existing CHD

<letter/sentence/paragraph deleted>

<letter/sentence/paragraph deleted>

Pfizer Clinical/Medical Colleagues:

Name: [redacted].[redacted], Dr PH
Title: ***Executive*** Director, Metabolic Diseases
Department: ***Pfizer Global Research and Development***

[redacted]
Signature

13 Jun 03
Date

Name: [redacted] [redacted]
Title: ***Mecical Director***
Department: ***Pfizer Global Pharmaceuticals***

[redacted]
Signature

6/23/03
Date

Name: [redacted] [redacted]
Title: ***Senior Director Biometrics***
Department: ***Pfizer Global Pharmaceuticals***

[redacted]
Signature

6/23/03
Date

The above signed confirms herewith to have read and understood this trial protocol and attached appendices, furthermore, to accomplish this study according to the protocol and the Good Clinical Practice guidelines, as well as local regulations, and to accept respective revisions conducted by authorized personnel ***<letter/sentence/paragraph deleted>*****of Pfizer and by competent authorities.**

PROTOCOL FOR ***<letter/sentence/paragraph deleted>*** PFIZER ***<letter/sentence/paragraph deleted>*** CLINICAL TRIAL

TITLE: The Effect Of LDL-Cholesterol Lowering Beyond Currently Recommended Minimum Targets On Coronary Heart Disease (CHD) Recurrence In Patients With Pre-Existing CHD

Principal Investigator:<br>Name:<br>Address:	______________________<br>Signature	________<br>Date
Co-Principal Investigator:<br>Name:<br>Address:	______________________<br>Signature	________<br>Date

The above signed confirms herewith to have read and understood this trial protocol and attached appendices, furthermore, to accomplish this study according to the protocol and the Good Clinical Practice guidelines, as well as local regulations, and to accept respective revisions conducted by authorized personnel *<letter/sentence/paragraph deleted>* **of Pfizer and by competent authorities.**

June 13, 2003 – Amendment 4

PROTOCOL FOR <***letter, sentence, paragraph deleted***> PFIZER <***letter, sentence, paragraph deleted***>CLINICAL TRIAL

TITLE: The Effect Of LDL-Cholesterol Lowering Beyond Currently Recommended Minimum Targets On Coronary Heart Disease (CHD) Recurrence In Patients With Pre-Existing CHD

INSTITUTIONAL REVIEW BOARD/ETHICS COMMITTEE:

INSTITUTIONAL REVIEW BOARD/ETHICS COMMITTEE ADDRESS:

COORDINATING INVESTIGATOR:

SUBINVESTIGATOR(S):

June 13, 2003 – Amendment 4

LABORATORY(IES): [redacted]

TIME PERIOD AND NUMBER OF PATIENTS

A.	Anticipated Starting Date of Study:	2Q1998
	Anticipated Starting Date for Site:	
B.	Anticipated Complete Date:	4Q2004
C.	Anticipated Number of Patients for Site:	*a minimum of 35*
D.	Anticipated Number of Sites:	250
E.	Anticipated Number of Patients for Study:	8,600

TABLE OF CONTENTS

Page

June 13, 2003 – Amendment 4

SYNOPSIS ii

1. INTRODUCTION 1

2. STUDY OBJECTIVE 4

3. SUMMARY OF STUDY 4

4. ETHICAL AND LEGAL CONSIDERATIONS 4

5. STUDY POPULATION 5
5.1. Source and Number of Patients 5
5.2. Patient-Selection Criteria 5
5.2.1. Inclusion Criteria 5
5.2.2. Exclusion Criteria 6
5.3. Prohibited Medications or Precautions 7
5.4. Allowable Concurrent Medications 8

6. STUDY DESIGN AND METHODOLOGY 8
6.1. Study Design 8
6.2. Study Schedule and Guidelines for Visits 9
6.2.1. Screening Visit (Visit 1) 9
6.2.2. Atorvastatin 10 mg Run-In Period 10
6.2.3. Rescreening and Retesting Prior to Randomization 11
6.2.4 Double-Blind Active Treatment Period 12
6.3. Efficacy Assessments 12
6.3.1. Primary Efficacy Parameters 13
6.3.2. Secondary Efficacy Parameters 13
6.3.3. End Point Committee 13
6.3.4. Clinical End Point Documentation 14
6.4. Safety Assessments 14
6.4.1. Laboratory Evaluation 14
6.4.1.1. Clinically Important Laboratory Abnormalities 15

TABLE OF CONTENTS

6.4.2. Adverse Event Reporting 16
6.4.2.1. Serious Adverse Events 17
6.4.2.2. Adverse Event Follow-up 18
6.4.2.3 Rechallenge Procedure 18
6.4.3. Other Safety Monitoring 19
6.4.4. Data and Safety Monitoring Board (DSMB) 19
6.4.5. Steering Committee 20
6.5. Economic Evaluation 20
6.6. Patient Follow-up/Discontinuation of Study Drug 20
6.7. Study Completion 21

7. STUDY MEDICATION 22
7.1. Formulation 22
7.2. Medication Dispensing 22
7.3. Dosage Regimen 22

8. DATA COLLECTION 22

9. DATA ANALYSIS AND STATISTICAL CONSIDERATIONS 23
9.1. Sample Size Considerations 23
9.2. Analysis Populations 24
9.3. Efficacy Paramaters 24
9.3.1. Primary Efficacy: Time to the Occurrence of Major Cardiovascular Events. 24
9.3.2. Secondary Efficacy: Definitions 25
9.3.2.1 Other Cardiovascular Morbidity and Mortality 25
9.3.2.2 All Cause Mortality 25
9.4. Methods of Analysis 25
9.4.1. Primary Efficacy Analysis 25
9.4.2. Secondary Efficacy 25
9.4.3 Covariate and Subgroup Analyses 26
9.5. Safety Parameters 26

TABLE OF CONTENTS

(Page 3 of 3)

9.6. Interim Analyses 26

9.6.1. Timing, Content 26

9.6.2. Early Stopping 26

9.6.3. Distribution of the Interim Analysis 27

10. ECONOMIC ANALYSES 27

11. DNA Databank 27

11.1 Introduction 27

11.2 Procedure 28

11.3 Process for Anonymity of Blood Samples 28

12. REFERENCES 28

13. LIST OF APPENDICIES 32

A. Timetable of Visits and Procedures 33

B. Commonly Prescribed Prohibited Medications 43

C. Study Safety Procedures and Clinical Laboratory Determinations 46

D. Administrative Procedures and Clinical Laboratory Determinations .... D-i

E. Other Administrative and Regulatory Procedures .... E-i

F. Declaration of Helsinki .... F-i

G. Amendments - October 6, 1998 .... G-1

H. Amendments - January 15, 1999 .... H-1

I. Amendments – June 4, 1999 .... I-1

J. Amendments – June 13, 2003 .... J-1

1. INTRODUCTION

The causal relationship between high serum cholesterol and the development of atherosclerosis is resolute.[1] Experimental models of atherosclerosis and observational studies in population groups show elevated serum total cholesterol and low-density lipoprotein cholesterol (LDL-C) to convey an increased risk of atherosclerotic disease development including coronary heart disease (CHD).[2,3] Decreasing CHD mortality rates are typically observed with lower cholesterol levels throughout the distribution of a population, with the steepness of the relationship lessening at the lower end of the cholesterol distribution.[4,5] In rural China, decreasing CHD mortality was observed down to total cholesterol levels of ~150 mg/dl (3.8 mmol/l).[6] Moreover, clinical trials reported to date with HMG-CoA reductase inhibitors have consistently shown a reduction in morbidity and mortality from CHD and other atherosclerotic diseases and associated events in patients with established CHD (secondary prevention), as well as in hyperlipidemic patients without clinical evidence of CHD (primary prevention).[7-10] As secondary prevention is the subject of this protocol further discussion will be limited to it.

As a result of the cumulated evidence, treatment guidelines for high blood cholesterol have emerged from several authoritative bodies. Two representative examples are the National Cholesterol Education Program-Adult Treatment Panel (NCEP-ATP) and the European Atherosclerosis Society (EAS) guidelines.

According to the NCEP-ATP II recommendations, drug treatment for secondary prevention is unequivocally recommended for patients whose LDL-C is greater than 130 mg/dl (3.4 mmol/l) and is left to clinical judgement for those whose levels are in the range of 100-129 mg/dl (2.6-3.4 mmol/l).[11] The target level of reduction is to ≤ 100 mg/dl (≤ 2.6 mmol/l). However, for patients who respond to therapy with LDL-C levels that are reduced to the 100-129 mg/dl range, clinical judgement of the risks and benefits are again recommended when considering elevations in dose or adding a second drug therapy in order to reach goal levels. The basis for the NCEP-ATP II target level recommendations were the cumulated epidemiological data reported to date as well as the emerging evidence from the initial coronary angiograghic trials showing a reduction in disease progression when these levels were achieved. The EAS guidelines for secondary prevention[12-13] provide for target LDL levels in the range of 115-135 mg/dl (3.0-3.5 mmol/l) with some authorities recommending a range of 95-115 mg/dl (2.5-3.0 mmol/l). In addition there is an emphasis on reducing total risk as much as possible rather than trying to achieve an "arbitrary and often impossible objective for an individual risk factor".[14]

Following the initial publication of the major authoritative guidelines, additional clinical endpoint and coronary angiographic trials have been reported. The first such study was the Scandinavian Simvastatin Survival Study (4S); a double-blind, placebo-controlled study in 4,444 patients with angina pectoris or prior myocardial infarction and who were frankly hypercholesterolemic (baseline LDL-C=188 mg/dl or 4.9 mmol/l).[15] Following an average 5.4 years of follow-up, the active treatment group (simvastatin 27 mg/day on average) had a reduction in LDL-C of 35% (to 122 mg/dl or 3.2 mmol/l) that was associated with a 30% decline in total mortality (primary endpoint) and a 26-34% reduction in other secondary coronary and cardiovascular endpoints.[7] Additional analyses tended to show similar reductions in risk of major coronary events for subgroups of patients classified by age, gender, smoking status, presence or absence of diabetes and

concurrent treatment with either aspirin, beta-blockers or calcium-channel blockers.[16-17] Results by baseline LDL-C also showed a consistent reduction in risk among the quartiles of this high cholesterol cohort; the lowest such quartile had an average LDL-C of ≤ 4.39 mmol/l (170 mg/dl).[18]

Costs of treatment to reduce cardiovascular risk factors may be offset by savings incurred by a reduction in hospital and ambulatory cost associated with myocardial infarction, coronary revascularization and long-term care for patients with existing heart disease. Two types of pharmacoeconomic analyses were undertaken in conjunction with the 4S trial: net cost analysis and cost-effectiveness analysis.[19] These cost analyses incorporated prospectively collected resource utilization data on cardiovascular hospitalizations and revascularization procedures. Decreases in resource utilization during simvastatin therapy (vs. Placebo, all $p<0.0001$) were shown for the number of hospitalizations for acute cardiovascular disease and CHD events, the number of coronary revascularization procedures and days spent in the hospital for the above events. Net cost analysis showed that 88% of the costs associated with simvastatin treatment could be saved by reductions in hospitalization and procedures over 5.4 years [20]. The cost-effectiveness ratios obtained showed that treatment with simvastatin is cost-effective in the markets studied [21,22]. The results of the economic portion of this current study will focus on the reduction of healthcare resource utilization associated with lowering LDL-C beyond the targets shown in the 4S study.

Observations from the 4S study were extended by the Cholesterol and Recurrent Events (CARE) Study.[23] This study in 4,159 post-myocardial infarction (3-20 months) patients with more normal cholesterol levels (LDL-C 115-174 mg/dl or 3.0-4.5 mmol/l) was also double-blind and placebo-controlled. Patients whose lipid levels were responsive to HMG-CoA-reductase inhibitor therapy were treated with 40 mg of pravastatin for a median of 5 years. A 32% reduction in LDL-C (to 98 mg/dl or 2.5 mmol/l) provided a 24% reduction in CHD death/non-fatal myocardial infarction. Similar reductions were seen in other coronary endpoints while total mortality, which was not designed as a primary endpoint, dropped by a non-significant 9%. Subgroup analyses showed a similarly robust reduction in coronary events among various patient groups as was seen with 4S with one notable exception; analyses by baseline LDL-C. Although those analyses included only some 400 patients in each treatment group, and had wide confidence limits, they showed no tendency for improvement in major coronary events for patients whose entry LDL-C was <125 mg/dl (3.2 mmol/l).[8] Results from the 9,014 patient Long-Term Intervention With Pravastatin in Ischemic Disease (LIPID) Study which utlized a similar population and treatment regimen to CARE, showed a 29% reduction in CHD. There was no statistically significant heterogeneity of the treatment effect among prespecified subgroups, including baseline LDL-C, with point estimates ranging from a 16% reduction to a 30% reduction in CHD for baseline LDL subgroups of <135 mg/dl and ≥ 174 mg/dl, respectively.[24]

While the observations from the CARE study has raised a question over the benefit of a further reduction in LDL-C in patients with relatively normal levels, the post-CABG study with lovastatin showed further improvement in angiographic findings and revascularization procedures when a baseline LDL-C of 155 mg/dl (4.0 mmol/l) was aggressively reduced to 93 mg/dl (2.4 mmol/l) in comparison to moderate reduction (LDL-C of 136 mg/dl or 3.5 mmol/l)[25]. Moreover, a recent analysis of 11 angiographic studies by Thompson et al [26] showed that it is the percent change in LDL-C and not the

on-trial LDL-C that predicts angiographic improvement, with observations from those trials ranging from on-treatment levels of 2.2 to 6.3 mmol/l (85-240 mg/dl). It should be noted that the only negative trial in these 11 was the HARP trial which enrolled subjects with the lowest baseline LDL-C, suggesting a lower threshold beyond which no further improvement could be expected. However, this is contrary to the recently published LCAS trial[27] showing angiographic improvement in patients with an entry LDL-C that was lower than HARP. If the HARP results are assumed to be anomalous, the implication is that the current emphasis of guidelines on target levels may not be correct in that the level of LDL-C after an atherosclerotic event is likely to be too high and that it needs to be lowered accordingly.[28]

In summary, current treatment guidelines call for aggressive treatment of cholesterol levels in patients with clinically evident atherosclerotic disease. While they are clearly supported by the clinical evidence at hand, recent angiographic findings also suggest that benefit may accrue to patients independent of their baseline LDL-C. In addition, there is both a paucity of clinical event data at low LDL-C target levels and conflicting evidence of the benefit afforded below the minimum targets set by expert groups. This study will therefore focus on providing evidence of the additional reduction in CHD found with achieving an average LDL-C level well below the currently accepted minium target level when compared to a level just meeting the currently accepted targets. The results of the study should provide supporting evidence to further optimize the evolving treatment strategies for the targeted high risk CHD population.

Atorvastatin is a new HMG-CoA reductase inhibitor that is available for clinical use at 10-80 mg/day (see reference (29) for review). Atorvastatin has an absolute bioavailability of 12%. It is highly protein bound (≥98%) and undergoes extensive first-pass metabolism in the liver as seen with other lipophilic statins. Metabolism of the parent compound though P450 3A4 produces pharmacologically active ortho- and para-hydroxylated metabolites. The $t_{1/2}$ of atorvastatin is approximately 12 hours and extends to 20-30 hours when including active metabolites. Elimination is predominantly hepatic with hepatic impairment resulting in significantly increased AUC values. Renal impairment has no effect on pharmacokinetic parameters.

In short-term dose-response studies, atorvastatin has been shown to provide mean reductions of approximately 40-60% in LDL-C and 15-40% in triglycerides (TG). The 10 mg starting dose of atorvastatin has been shown to provide superior LDL-C and TG lowering than other statins.[30-32] After 52 weeks of treatment at 80 mg/day, mean reductions in LDL-C and TG were 52% and 33%, respectively, in 189 severely hypercholesterolemic patients; these reductions were greater than those observed with 40 mg of simvastatin in combination with 20 grams of colestipol.[33] The superior reductions in LDL-C and TG seen with atorvastatin are hypothesized to be a result of a more constant inhibition of HMG CoA reductase, greater decrease in intracellular cholesterol levels and decreased production and secretion of VLDL particles.[29] This explanation is supported by the approximate 20% reduction in LDL-C observed with atorvastatin in receptor-negative homozygous FH patients.

The safety profile and toleration of atorvastatin appears to be similar to other statins. Discontinuation of therapy due to drug-related adverse events was <2% in clinical trials. The predominant clinical adverse effect appears to be of low frequency and gastrointestinal in nature. Raised serum transaminase levels occurred in 0.7% of patients

in clinical trials and, like other statins, appeared to be dose-related ranging from 0.2% at 10 mg to 2.3% at 80 mg. No confirmed cases of myopathy (confirmed CPK elevations >10 times the upper limit of normal with muscle symptoms) were seen in 2,502 patients treated in clinical trials.[29]

As with other statins, atorvastatin is contraindicated in patients with active hepatic disease or unexplained elevations in serum transaminases. Concomitant use with cyclosporin, nicotinic acid, fibric acid derivatives, erythromycin or azole antifungals is likely to increase the risk of musculoskeletal adverse events and should be avoided. Atorvastatin, when coadministered with digoxin, has been shown to increase digoxin levels by approximately 20%. No clinically important pharmacodynamic or pharmacokinetic interaction has been seen with atorvastatin and concurrent antacids, antipyrine, colestipol, cimetidine, or warfarin administration [29].

2. STUDY OBJECTIVE

June 13, 2003 – Amendment 4

The primary objective of this study is to determine the degree of additional reduction in ***cardiovascular*** risk that will ***accrue*** to patients by lowering their LDL-C beyond the currently accepted minimum target level for patients with pre-existing CHD. Subsidiary objectives include the safety profile of this treatment strategy, its cost-effectiveness, effect on other atherosclerotic-related events and procedures and total mortality.

3. SUMMARY OF STUDY

June 13, 2003 – Amendment 4

This is a double-blind, randomized, parallel-group study of the recurrence of ***cardiovascular events*** in patients whose LDL-C will be lowered on average to approximately 100 mg/dl (2.6 mmol/l) compared with those whose LDL-C is approximately 25% lower. Patients will have clinically evident CHD. The study has 3 periods: (1) a screening visit to determine a patient's initial eligibility to participate in the study, (2) an open-label atorvastatin 10 mg run-in period whose primary purpose is to achieve an LDL-C <130 mg/dl (3.4 mmol/l) and determine baseline study parameters, and (3) a randomized double-blind treatment period (atorvastatin 10 vs. 80 mg once daily) expected to require an average ***5.5*** years duration ***<letter, sentence, paragraph deleted>*** until the target number of ***events*** is achieved ***<letter, sentence, paragraph deleted>.***

4. ETHICAL AND LEGAL CONSIDERATIONS[a]

June 13, 2003 – Amendment 4

This study will be conducted in accordance with the Declaration of Helsinki and according to the guidelines in the attached appendices and in compliance with all applicable laws and regulations of the locale and country where the study is conducted.

This study will not be initiated at any site until the protocol and a copy of the informed consent document have been reviewed and approved by a duly constituted institutional review board (IRB) or ethics committee (EC) and meets local institutional requirements. Each patient and/or their legal guardian (or caregiver) shall read, understand, and sign an informed consent form.

---

[a] See Appendix E, Other Administrative and Regulatory Procedures (Sections 1.1, IRB/EC Reviews and Approval of Study; 1.2, Patient Informed Consent; 1.8, Confidentiality of Patient Information; and 2.1, Information Required for Regulatory Review) and Appendix F, The Declaration of Helsinki (and draft informed consent statement)

It is the responsibility of the investigator that the patient is made aware, and consent is given, that personal information may be scrutinized during audit by competent authorities and properly authorized persons but that personal information will be treated as strictly confidential and not be publicly available.

All revisions and/or amendments to the protocol must be approved in advance in writing by the investigator, the Pfizer ***<letter, sentence, paragraph deleted>*** clinical monitor(s) or their designee, and the appropriate Ethical Review Committee. These will be filed with the Food and Drug Administration in the United States by the Regulatory Affairs Department of ***<letter, sentence, paragraph deleted> Pfizer*** or its designee. The investigator is responsible for the retention of the patient log and patient records.

5. STUDY POPULATION

5.1. Source and Number of Patients

Source: Approximately 150-250 medical care facilities will participate. These centers will consist of providers of medical care treating patients with primary hyperlipidemia with coexistent coronary, cerebral, or peripheral vascular disease. Each center will be responsible for overseeing a total randomization of at least 35 patients into 2 treatment arms: drug therapy to achieve an LDL-C goal of approximately 100 mg/dl (2.6 mmol/l) using atorvastatin 10 mg once daily or, an LDL-C goal approximately 25% below that using atorvastatin 80 mg once daily.

Number of patients: approximately 8,600 total patients randomized

5.2. Patient-Selection Criteria

5.2.1. Inclusion Criteria

<u>At Screening</u>:

1. Men or women. Women of child-bearing potential must be using adequate measures of contraception (as determined by the investigator) to avoid pregnancy and should be highly unlikely to conceive during the study period. Women of child-bearing potential must have a negative pregnancy test.

2. Age 35-75 years.

3. Have clinically evident CHD defined as at least one of the following clinical manifestations of coronary atherosclerosis.
   a) prior myocardial infarction 1-60 months prior to screening;
   b) prior or present angina with objective evidence of atherosclerotic CHD. As a surrogate for unstable /severe angina, patients qualifying based on this criterion alone must have also been hospitalized with a discharge diagnosis of angina. The hospitalization must have been for management of angina and not for diagnostic work alone. The hospitalization must have occurred 1-60 months prior to screening. Objective evidence of atherosclerotic CHD requires documentation of one or more of the following:

- A reversible defect on nuclear stress testing. The stress must be exercise, adenosine, dobutamine or dipyridamole. The imaging agent must be thallium, sestamibi, or tetrafosmin. Fixed defects or defects reported as "possible artifact" or "possible ischemia" do not meet this criterion.
- A reversible wall motion abnormality on echocardiographic stress testing. The stress must be exercise, adenosine, dobutamine or dipyridamole. Fixed wall motion abnormalities, or abnormalities considered to be borderline, or possibly not due to ischemia, do not meet this criterion.
- The demonstration by coronary angiography of a 50% or greater diameter stenosis in at least one of the major vessels (left main, LAD, circumflex, or dominant RCA). Stenoses in non-dominant RCA, or diagonal, marginal or posterolateral branches do not meet this criterion.

c) prior coronary revascularization procedure as follows:
CABG: any prior occurrence
PTCA or other procedure: 1-60 months prior to screening

<u>At Visit 2</u>:

4. an LDL-C and TG profile as follows:
   a) LDL-C 130-250 mg/dl (3.4-6.5 mmol/l) and,
   b) TG ≤600 mg/dl (6.8 mmol/l)

<u>At Visit 3 and 4</u>:

5. an LDL-C and TG profile as follows:
   a) LDL-C < 130 mg/dl (< 3.4 mmol/l) based on the average from Visits 3 and 4,
   b) TG ≤ 600 mg/dl (6.8 mmol/l) at both Visits 3 and 4

5.2.2. Exclusion Criteria

1. A contraindication to HMG Co-A reductase therapy as follows (any one or more):
   a) known hypersensitivity to any HMG Co-A reductase inhibitor
   b) active liver disease or hepatic dysfunction with AST or ALT ≥1.5 times the upper limit of normal (ULN) as determined at any time between screening and randomization
   c) women who are breast feeding or are pregnant

2. Evidence of secondary hyperlipidemia (any one or more):
   a) nephrotic syndrome
   b) uncontrolled diabetes mellitus Type 1 or 2 defined by an HbA_{1C} >10% at screening (by central laboratory)
   c) uncontrolled hypothyroidism defined by a TSH> 1.5 times the ULN at screening (by central laboratory)

3. Evidence of other uncontrolled or concurrent conditions or medications that may obscure the efficacy or safety comparisons:
   a) uncontrolled hypertension (investigator defined) at the screening visit
   b) a myocardial infarction, coronary angioplasty, coronary artery bypass graft, other revascularization procedure, or severe/unstable angina or other hospitalization for an atherosclerotic condition within one month of the screening visit

c) any planned surgical procedure for the treatment of atherosclerosis
d) an ejection fraction (if known) of <30%
e) hemodynamically important valvular disease
f) GI disease limiting drug absorption or, partial ileal bypass
g) any non-skin malignancy, malignant melanoma, or other survival limiting disease (including class IIIb/IV congestive heart failure, life-threatening arrhythmias and candidates for organ transplant). Patients with a history of the above cancers are not eligible if any treatment (medical, surgical, radiological) and/or recurrence was clinically evident in the 10 years prior to screening.
h) a CPK > 6 times the upper limit of normal (at visit 1) that is unexplained
i) other significant abnormalities that the investigator feels may compromise the patient's safety or successful participation in the study
j) patients taking any of the prohibited medications specified in Section 5.3 (below). Patients with concurrent conditions that require frequent use of prohibited medications should be excluded.

4. Potential for noncompliance and other administrative issues
   a) abuse of alcohol, other substance abuse or poor mental function
   b) compliance rate <80% during the atorvastatin 10 mg run-in period as assessed by tablet count
   c) participation in another clinical trial within 30 days of the screening visit

5.3. Prohibited Medications or Precautions

June 13, 2003 – Amendment 4

The following medications are not be taken at any time during this study:

- Lipid-regulating drugs not specified as study treatment in the protocol: probucol, fibrates and derivatives, bile-acid sequestering resins, other HMG-CoA reductase inhibitors, niacin (>500 mg/day), and supplemental fish oils (prescription only). HMG-CoA reductase inhibitors may only be taken as described in the protocol. ***Following the completion of year 1, patients will be allowed to utilize certain lipid-regulating drugs ("add-on therapy") if the patient's LDL levels are found to be higher than protocol range (see section 6.4.1). "Add-on" therapy will be agreed upon by investigator and Pfizer medical personnel or designee.***

- Patients taking a lipid-regulating drug(s) must have the medication discontinued at or prior to the screening visit to be eligible for screening, except in the case of probucol where medication must have been discontinued for at least 12 months.

The following medications should not be administered concurrently with study drug.

- Any immunosuppressive agent (e.g., cyclosporin).

- Other drugs known to be associated with an increased risk of rhabdomyolysis in combination with HMG-CoA reductase inhibitors [including: erythromycin, clarithromycin, any chronic systemic antifungal use, mibefradil dihydrochloride, nefazodone, fluvoxamine and other potent inhibitors of cytochrome P450-3A4]. Coadministration of atorvastatin (10 mg) and azithromycin (500 mg QD) has been shown not to alter plasma concentrations of atorvastatin.

Should these medications be necessary during the course of the trial, consideration should be given to temporarily withholding study drug and restarting it only after time has elapsed to allow for sufficient clearance of the medication. In addition, temporarily withholding study drug should be considered in any patient with a risk factor predisposing to the development of renal failure secondary to rhabdomyolysis, including severe acute infection; hypotension; major surgery; trauma; severe metabolic, endocrine, or electrolyte disorders; and uncontrolled seizures.

A list of the commonly prescribed medications discussed above may be found in Appendix B.

As co-administration of atorvastatin (80 mg) and digoxin increased steady-state plasma digoxin concentrations by approximately 20%, patients taking digoxin should be monitored appropriately.

While atorvastatin had no clinically significant effect on prothrombin time when administered to patients receiving chronic warfarin therapy, prothrombin time should be monitored as clinically appropriate.

5.4 Allowable Concurrent Medications

The dosage and regimen of any chronic, permitted concurrent medications (e.g., calcium channel blockers, b-blockers, hormone replacement therapy, hormone contraceptives, thiazides, loop diuretics, H-2 antagonists, aspirin, phenytoin) should be stabilized (for at least 30 days) before the open-label run-in phase begins (Visit 2) and kept constant (as is medically appropriate) throughout the study. Any medications prescribed chronically or intermittently during the study must be reported on the concurrent medication Case Report Form (CRF). The occasional (< 3 times per week) use of antacids is permitted.

6. STUDY DESIGN AND METHODOLOGY

June 13, 2003 – Amendment 4

6.1. Study Design

This is a double-blind, randomized, parallel-group study of the recurrence of ***cardiovascular events*** in patients whose LDL-C will be lowered on average to approximately 100 mg/dl (2.6 mmol/l) compared with those whose LDL-C is approximately 25% lower. Patients will have clinically evident CHD. Following dietary therapy, all patients meeting initial eligibility criteria will begin an open-label atorvastatin 10 mg (once daily) run-in period. Patients whose LDL-C is < 130 mg/dl (< 3.4 mmol/l) at the end of the run-in period (average of Visits 3 and 4) and meet all other inclusion/exclusion criteria will be randomized to double-blind therapy consisting of either atorvastatin 10 mg/day or 80 mg/day. Treatment and follow-up of patients will be continued until ***<letter, sentence, paragraph deleted>*** the number of ***major coronary***

events (CHD death, non-fatal myocardial infarction, or resuscitated cardiac arrest) reaches 750 ***(approximately 5.5 years average follow-up).*** Early stopping rules may be applied based on interim analyses.

6.2. Study Schedule and Guidelines For Visits

A study timetable and the specific procedures to be performed at each visit are shown in Appendix A. However, a patient may be seen at any time for reasons of safety. All laboratory procedures such as clinical laboratory measurements and lipoprotein determinations will be performed by a central laboratory.

The timing of clinic visits should be consistent throughout the study and the study medication should be taken at approximately the same time each day. When laboratory determinations are required for a scheduled study visit, blood draws must occur after a minimum 12-hour fast (water allowed). If this is not possible, the visit must be rescheduled in a timeframe to allow for continuous dosing of study drug (~10-20 days dependent on study period and visit interval). Patients should have been generally compliant with study medication in the two week period prior to a clinic visit. If a patient has not been compliant or has been temporarily discontinued from medication, the clinic visit is to be rescheduled to allow for a two week period on study medication.

6.2.1. Screening Visit (Visit 1)

This single visit allows for an initial assessment of those patients the investigator feels may eventually qualify for randomization into the study. At screening an informed consent will be signed, demographic characteristics assessed, vital signs measured, all concomitant medications documented, and a medical history recorded. The investigator should screen for potential causes of secondary hyperlipidemia. These should be identified and corrected, with laboratory verification, prior to Visit 2. TSH and HbA_{1c} measurements (for known or suspected diabetics) are obtained at Visit 1 for this purpose. Patients who appear to meet the demographic and medical history criteria should stop any prohibited medications at this visit (see section 5.3). Patients will have blood drawn for fasting lipid determinations and a standard clinical profile. LDL-C will be derived from the lipid profile (See Appendix A4 for methodology) in order to help the investigator project whether the patient will meet the inclusion criteria to enter the open-label run-in phase after dietary stabilization.

The CHD entry criteria must be documented (prior to Visit 2) through either the investigational site's medical records, or copies of such records from other institutions, or a letter from a referring physician that specifically states the diagnosis and date of the most recent occurrence of the qualifying event.

The investigator or designee will perform a general review of the patient's current dietary intake at the screening visit. The patient should receive diet information and/or other aids normally used by the site to obtain compliance with an NCEP Step I, Step II, or other equivalent diet.

The timing of Visit 2 is variable as follows. All patients previously on lipid-altering drug(s) must have these medications discontinued at the screening visit and must have at

least a 4 week washout prior to Visit 2. If the investigator is familiar with the status of the patient and is confident that the patient is already following a rigorous diet (NCEP Step I or Step II diet or equivalent) and that the patient's lipid levels are stabilized, Visit 2 (Week -8) should be scheduled either 1 week (no previous lipid therapy) or 4 weeks (on prior lipid therapy) following the initial screening visit. If the investigator is not confident that the patient is following a rigorous diet, or if this is a new patient, additional time may be needed to ensure that lipids are stabilized before randomization. In this later case, the patient should be instructed to initiate the NCEP Step I, Step II, or equivalent diet and up to 8 weeks should be allowed between the screening visit and Visit 2 (Week -8). In all cases, the screening visit must be completed no more than 8 weeks prior to entry into the open-label atorvastatin 10 mg run-in period.

If the patient has none of the screening exclusion criteria (Section 5.2.2) and has lipid levels at screening that are likely to result in entry into the active treatment phase (i.e., generally no higher than 20% above the upper limit or lower than 20% below the lower limit of the Visit 2 requirements), the patient may be entered into the open-label run-in period.

6.2.2 Atorvastatin 10 mg Run-In Period

The purpose of this period is to further assess a patient's qualification for randomization based on response to atorvastatin 10 mg once daily (open-label), determine initial study medication compliance, and establish baseline values for a number of study parameters.

This period begins with Visit 2 (Week -8) and ends with randomization at Visit 5 (Week 0). Procedures for each visit are as noted in Appendix A, and Visit 2 is scheduled as noted in the prior section. Patients will be queried regarding the occurrence of adverse events and any changes or additions to concomitant medications at all open-label run-in visits.

At Visit 2, vital signs will be assessed, dietary counseling will continue and lipids will be measured. In addition, the patient will be dispensed open-label atorvastatin (10 mg) to be taken once daily. If the Visit 2 LDL-C is 130-250 mg/dl (3.4-6.5 mmol/l) and TG is ≤ 600 mg/dl (6.8 mmol/l) and the patient continues to have no exclusion criteria, they may be scheduled for Visit 3 (Week -4) which should occur approximately four weeks after Visit 2.

At Visit 3 (Week -4) and Visit 4 (Week -2), lipid determinations will again be made, dietary counseling will continue, and patients will continue to use open-label atorvastatin (10 mg) once daily.

Patients are eligible to return for Visit 5 if their average LDL-C (Visits 3 and 4) is <130 mg/dl (<3.4 mmol/l), TG ≤600 mg/dl (<6.8 mmol/l) at both Visits 3 and 4, and patients have been tolerant of, and compliant with, study medication at Visits 3 and 4. Visit 5 (Week 0, randomization) should be scheduled for two weeks following Visit 4.

NOTE: Patients who, after the screening visit but before randomization, experience a cardiovascular event (e.g., non-fatal MI) or develop an exclusionary condition (e.g., systemic malignancy) must be discontinued and re-evaluated for eligibility according to the timing and criteria of the screening visit *(see section 6.2.3 below).*

A summary of the pre-randomization period (Visits 1-4) along with their timing and key criteria for patient continuation is found below.

Visit	Timing	Diet	Drug	Key Criteria for Patient Continuation
1	Variable (9-16 weeks prior to randomization)	Yes	Washout	• Informed consent and eligible demographics/ medical history

Visit	Timing	Diet	Drug	Key Criteria for Patient Continuation
2	Week -8	Yes	Open-label 10 mg atorvastatin begins	• LDL-C 130-250 mg/dl (3.4-6.5 mmol/l)<br>• TG ≤ 600 mg/dl (<6.8 mmol/l)
3	Week -4	Yes	Continue as in V2	
4	Week -2	Yes	Continue as in V2	• Average LDL-C <130 mg/dl (<3.4 mmol/l) from Visits 3 and 4<br>• TG ≤ 600 mg/dl (<6.8 mmol/l) at both Visits 3 and 4)<br>• Tolerant and compliant with study medication

June 13, 2003 – Amendment 4

6.2.3 Rescreening and Retesting Prior to Randomization

Patients may be rescreened once for the study if they experience a condition prior to randomization that makes them ineligible to continue in the study. The condition will need to be treated/stablized (e.g., uncontrolled diabetes, hypothyroidism, experiencing a potential endpoint) prior to consideration for rescreening. Rescreening of a patient must have prior approval of the ***<letter, sentence, paragraph deleted>*** Pfizer medical monitor or their designee.

Patients may be retested during the pre-randomization period only once for laboratory values that make them ineligible for the study (e.g. exclusionary levels of CPK and ALT/AST). Retesting will not be allowed for lipid eligibility at Visits 2, 3, or 4. Patients failing to meet eligibility based on retesting must be discontinued and are not eligible for rescreening. Retesting of a patient must have prior approval of the ***<letter, sentence, paragraph deleted>*** Pfizer medical monitor or their designee.

6.2.4 Double-Blind Active Treatment Period

This period begins at Visit 5 (Week 0). Procedures performed at this visit include an assessment of vital signs, an ECG and physical exam, repeat clinical chemistry/fasting lipid determinations and continued dietary counseling. The ECG and physical exam may be performed between Visits 4 and 5 once a patient's lipid levels qualify them for randomization.

The open-label atorvastatin 10 mg therapy will stop at Visit 5. Qualified patients will be randomized to 1 of 2 blinded treatment groups, either 80 mg atorvastatin or 10 mg atorvastatin. The patient should take the study medication once daily. Neither the investigator nor the study sponsor will be informed of the lipid values of randomized patients (except as noted in section 6.4.1) in order to maintain the blind of the study.

Visits will be at designated intervals during the active treatment period. Patients will be regularly counseled on following the NCEP Step I, Step II (see Appendix A.3) or equivalent diet in order to maintain body weight, and lipid levels. Other procedures such as lipid profiles, clinical laboratory measurements, physical examinations, and electrocardiograms will be performed according to the timetable in Appendix A. Patients will be assessed for the occurrence of adverse events and the change or addition of concomitant therapies at all visits in the double-blind treatment period. Medical resource utilization regarding all hospitalizations and revascularization procedures will be collected.

During the first year of the double-blind period, four follow-up visits are scheduled at weeks 12 and months 6, 9 and 12.

After completion of the first year of active treatment, patients are to have scheduled study visits every six months. At each visit, vital signs, clinical end points, adverse event, and concurrent medication information will be collected. On alternating visits (i.e., annually) in addition to the above, physical examinations and electrocardiograms will be performed and laboratory specimens will be collected.

While on average a patient will be on active treatment for 5 years, the actual length of time for an individual patient will vary based on the study recruitment rate, primary event rate and when the patient was recruited relative to the entire study cohort. Therefore, it is estimated that actual active treatment for a patient could range from approximately 3 to 7 years.

6.3. Efficacy Assessments

Clinical end points defined as primary and secondary efficacy parameters will be assessed beginning with the first day of double-blind treatment. Although excluded from the efficacy assessments, clinical end points occurring between screening and double-blind treatment will also be identified. Lipid and lipoprotein changes from baseline defined as efficacy parameters will be assessed beginning with the first measurements recorded after initiation of the double-blind treatment period. Evaluation of the primary and secondary efficacy parameters is described below. A complete description of these end points will be detailed in a separate End Point Committee Manual Of Operations.

June 13, 2003 – Amendment 4

6.3.1. Primary Efficacy Parameters

The primary efficacy clinical end point is the occurrence of a major ***cardiovascular*** event, defined as either:

-CHD death, ***<letter, sentence, paragraph deleted>***
-non-fatal myocardial infarction
-Resuscitated cardiac arrest or
-stroke (fatal or non- fatal)

June 13, 2003 – Amendment 4

6.3.2. Secondary Efficacy Parameters

The secondary efficacy clinical end points are the occurrence of the following:

-major coronary event (CHD death, non-fatal myocardial infarction or resuscitated cardiac arrest)
-any coronary event (***major coronary event*** or, CABG, PTCA, other revascularization procedure, ***procedure-related myocardial infarction*** or documented angina)
-cerebrovascular event (fatal or non-fatal stroke, TIA)
-peripheral vascular disease (PVD)
-hospitalization with primary diagnosis of CHF
-any cardiovascular event (any of the above)
-all cause mortality

In order to validly assess the primary and secondary endpoints and the safety profile of the treatment groups, the vital status of each randomized patient must be obtained at the completion of the study.

In addition, the assessment will include as secondary end points the changes from Visit 2 and baseline in the various lipid and lipoprotein parameters included in the study (Appendix A.4) as well as the relationship between the occurrence of the primary and secondary clinical end points defined above with baseline, relative and absolute changes from baseline in these lipid and lipoprotein parameters.

Subgroup analyses will also be performed to investigate variation in the treatment effect of the primary and secondary efficacy end points by the following: age, gender, smoking status, presence or absence of diabetes, ***baseline*** TG (< 200, ≥ 200 mg/dl), ***baseline LDL, baseline HDL,*** CHF, other prevalent pre-existing condition categories of the study cohort, and prevalent concomitant medication use (***such as*** hormone replacement therapy, aspirin, beta-blockers, ***<letter, sentence, paragraph deleted>*** calcium channel blockers, ***etc.).***

6.3.3. End Point Committee

An end point committee will review (blinded) all primary end points and all secondary end points to confirm that the data supports the end point designation. The committee members who perform individual end point review will not be investigators or sub-investigators in the trial. Differences in individual review designations will be

adjudicated by conference calls or regular meetings. A separate Manual Of Operations will fully describe the methods to be used by the End Point Committee.

June 13, 2003 – Amendment 4
6.3.4. Clinical End Point Documentation

The identification of potential clinical end points will be made by the study site and communicated to ***<letter, sentence, paragraph deleted>*** Pfizer (or their designee) immediately (within 24 hours). Obtaining the documentation required to support end point adjudication by the end point committee will be the responsibility of the study site. End point documentation will include, but is not limited to, hospital discharge summaries, operative reports, clinic notes, ECGs, diagnostic enzymes, results of other diagnostic tests, autopsy reports and death certificate information. Specific end point documentation will be submitted as requested by ***<letter, sentence, paragraph deleted>*** Pfizer (or their designee).

6.4. Safety Assessments [b]

June 13, 2003 – Amendment 4
6.4.1. Laboratory Evaluation

A full clinical laboratory determination will be performed by a central laboratory at screening (Visit 1) and at Visits 5, 9, and annually thereafter as shown in Appendix A. An abbreviated clinical laboratory determination of key safety[b] parameters will be performed at Visits 4 and 6.

A clinically significant laboratory abnormality, as defined in Section 6.4.1.1., occurring during the study will be reported as an adverse event and followed until the abnormality has resolved or a satisfactory explanation has been obtained. (See Appendix C for a listing of the clinical laboratory determinations to be performed and Appendix D for reporting of significant laboratory abnormalities.)

Following randomization, lipid and lipoprotein determinations (including those at Visit 5) will not be reported to study sites. The central lab will monitor LDL values, starting at Visit 9 and annually thereafter, and triglyceride values, starting at Visit 6 and annually thereafter and if necessary action will be taken according to the following plan:

LDL Action Plan. If the LDL level at a scheduled annual visit (visits 9, 11, 13, 15, 17...) is either ≥130 mg/dl (3.4 mmol/l) or <50 mg/dl (<1.3 mmol/l), the central laboratory will notify the study site that the level is "outside protocol limits" (the specific LDL value will remain blinded). The site will have the patient return for a repeat fasting lipid panel and TSH determination within 3-4 weeks. During the period prior to retest, the site will be contacted by the ***<letter, sentence, paragraph deleted>*** Pfizer medical monitor or their designee to reinforce compliance with study medication and non-pharmacological hygienic measures. Actions based on retest result levels are as follows:

- 25-130 mg/dl (~0.7-3.4 mmol/l). LDL levels and treatment group will continue to be blinded. Study medication should continue and no further action is required for this patient group.

---

[b] See Appendix C, Detailed Study Safety Procedures and Clinical Laboratory Determinations

- <25 mg/dl (<0.7mmol/l). LDL levels will continue to be blinded ***<letter, sentence, paragraph deleted>***. It is recommended that study medication continue however, in discussion with the ***<letter, sentence, paragraph deleted>*** Pfizer medical monitor or their designee, various alternatives for continued treatment will be discussed and the site will document a treatment plan. The preferred treatment action for this patient group is to remain on study medication.

- 131-160 mg/dl (~3.4-4.1 mmol/l). LDL levels and treatment group will continue to be blinded. Study medication should be continued. Retests are to be done at the next scheduled visit and sites will be requested to continue to reinforce compliance with study medication and non-pharmacological hygienic measures. If at semi-annual retest, the LDL continues in the 131-160 range, repeat determination will be managed as described below in the >160 mg/dl group.

- >160 mg/dl (>4.1 mmol/l). LDL levels and treatment group will continue to be blinded. Study medication should be continued. In discussion with the ***<letter, sentence, paragraph deleted>*** Pfizer medical monitor or their designee, various alternatives for add-on therapy ***or use of open-label atorvastatin*** will be discussed and the site will be required to document a follow-up and treatment plan.

Triglyceride Action Plan. If the triglyceride level at any post-randomization scheduled visit is ***≥500*** mg/dl (***<letter, sentence, paragraph deleted>***mmol/l), the central laboratory will notify the study site that the level is in the critical range and the value will be reported. The site will be requested to have the patient return for a repeat determination within 2 weeks. During the period prior to retest, the site will be contacted by the ***<letter, sentence, paragraph deleted>*** Pfizer medical monitor or their designee to reinforce compliance with study medication, low carbohydrate diet and restriction of alcohol intake. Actions based on retest result levels are as follows:

- *<500* mg/dl (*5.6* mmol/l). Triglyceride levels and treatment group will be blinded and study medication should be continued. No further action is required for this patient group.

- ***≥500*** mg/dl (*5.6* mmol/l). Triglyceride levels ***will be unblinded while out of range***. In discussion with the ***<letter, sentence, paragraph deleted>*** Pfizer medical monitor or their designee, various alternatives for continued treatment will be discussed including add-on therapy. The site will be required to document a follow-up and treatment plan.

June 13, 2003 – Amendment 4

6.4.1.1. Clinically Important Laboratory Abnormalities

The following laboratory abnormalities are arbitrarily designated as clinically important and therefore are to be reported as adverse events.

- CPK > 10 times the ULN at 2 consecutive measurements 4 to 10 days apart accompanied by muscle tenderness or weakness (patients should be advised to report promptly unexplained muscle pain, tenderness, or weakness, especially if accompanied by malaise or fever).

- ALT/AST > 3 times the ULN at 2 consecutive measurements 4 to 10 days apart.

If either of the above abnormalities occur, the ***<letter, sentence, paragraph deleted>*** Pfizer study manager or clinical monitor (or their designees) should be contacted immediately.

If at any time during the study the patient's ALT or AST levels increase to >3 × ULN range, the patient should be scheduled for a repeat laboratory measurement within 1 week (±3 days). If the repeat value is still >3 × ULN range, the patient should have their double-blind medication discontinued but should continue their participation in the study for the identification of clinical end points (see Section 6.6).

If at any time during the study, a patient either:

- reports unexplained muscle pain, tenderness, or weakness (especially if accompanied by malaise or fever), or

- their CPK level increases to >10 x the ULN,

study medication should be temporarily discontinued and CPK measurements performed by the central laboratory within 1 week (±3 days). If the repeat CPK levels are > 10 x the ULN with muscle pain, tenderness or weakness, the patient should have their double-blind medication discontinued but continue their participation in the study for identification of clinical end points (see Section 6.6).

Following normalization of a clinically important laboratory abnormality considered causally related to atorvastatin, the patient may be rechallenged with study medication. Rechallenges must be discussed with, and approved by, the ***<letter, sentence, paragraph deleted>*** Pfizer clinical monitor (or their designee) and appropriate IRB/EC. These patients will maintain their original treatment group assignment during rechallenge. A rechallenge is defined as reinstitution of study drug following discontinuation for a medically significant adverse event considered to be caused by atorvastatin. (See Section 6.4.2.3 for further details)

6.4.2. Adverse Event Reporting [c]

Each patient will be observed and queried in a nonspecific fashion at each visit after screening (Visit 1) during the study for any new or continuing symptoms since the previous visit. All serious and nonserious adverse events must be recorded on the

[c] See Appendix D, Administrative Procedures For The Reporting Of Adverse Events

appropriate CRF including the onset, duration, severity, relationship to study drug and ultimate management as detailed in Appendix D.

Rechallenges for medically significant adverse events that are considered to be caused by atorvastatin should follow the procedures outlined in Section 6.4.2.3.

Clinical end points and associated symptoms as defined in Section 6.3, are considered efficacy parameters and are not to be reported as adverse events.
Procedural-related complications that do not meet the definition of a clinical end point as defined in Sections 6.3 will be considered an adverse event. For example, pneumonia during the hospital stay for a CABG will be considered an adverse event, while a myocardial infarction resulting from a CABG will be considered a clinical end point.

June 13, 2003 – Amendment 4

6.4.2.1. Serious Adverse Events[(d)]

Except as noted previously for clinical end points, a serious adverse event is any experience that suggests a significant hazard, contraindication, side effect, or precaution. A serious adverse event includes an experience that:

- ***Results in death;***
- ***Is life-threating;***
- ***Results in inpatient hospitalization or prolongation of existing hospitalization***
- ***Results in a persistent or significant disability/incapacity; or***
- ***Results in congenital anomaly/birth defect***

Hospitalizations for diagnostic procedures performed for underlying coronary disease that are not in association with acute medical events shall not be reported as SAEs provided: 1) The procedure was ordered in routine management of the underlying disease state for which the patient is being treated in the study. 2) The patient was not hospitalized for a worsening of the underlying disease state. 3) The patient was not hospitalized for an acute event which subsequently led to a procedure.

Overdoses with an associated adverse event are to be reported as serious adverse events. Overdoses are defined as follows:

- Pre-Randomization: greater than 8 tablets of open label drug taken in a 24 hour period.
- Post Randomization: greater than 8 tablets from bottle A, or greater than 2 tablets from bottle B, taken in a 24 hour period.

Appendix D provides serious adverse event definitions as well as administrative and reporting information.

IN CASE OF SERIOUS OR LIFE-THREATENING ADVERSE EVENTS, OR IN THE EVENT OF DEATH, THE *<letter, sentence, paragraph deleted>* PFIZER STUDY MANAGER OR CLINICAL MONITOR (or their designees) SHOULD BE CONTACTED IMMEDIATELY.

[(d)] See Appendix D, Administrative Procedures for the Reporting of Adverse Events (Section 4.1, Immediately Reportable Adverse Events)

If any serious adverse event occurs, study treatment should be interrupted or discontinued at the investigator's discretion except as specified by the protocol. If in an acute medical emergency the ***<letter, sentence, paragraph deleted>*** Pfizer monitor (or their designees) cannot be contacted, the randomization code may be broken only if this is required for proper treatment of the patient.[e] Notify the ***<letter, sentence, paragraph deleted>*** Pfizer Clinical Monitor (or their designees) of emergency code breaks as soon as possible.

June 13, 2003 – Amendment 4

6.4.2.2. Adverse Event Follow-up

Adverse events reported during the screening, open-label run-in, double-blind treatment periods and up to ***30*** days after the cessation of treatment ***or final follow-up visit required by the protocol (whichever comes later)*** should be recorded and followed until the adverse event has subsided and abnormal findings have returned to normal or stabilized.

6.4.2.3 Rechallenge Procedure

If after normalization of a study medication related adverse event or significant lab abnormality, rechallenge with study medication is to follow the procedure described below. The rechallenge must be approved by the appropriate sponsor medical personnel or their designees. The rechallange process as well as the risks and benefits of rechallenge should be discussed with the patient. If the physician and patient agree, the patient will be asked to sign the appropriate, IRB/EC approved (and sponsor approved if applicable) consent form addendum for the rechallenge. One of two options for restarting study medication is to followed:

1. Patient will be re-started at full dose of study medication (1-A and 2-B tablets). For laboratory abnormalities, patients will have follow-up labs at 6,12,18, and 24 weeks post-start or at a frequency agreed to between the sponsor medical team and investigator. For non-laboratory abnormality related rechallenges, follow-up visits should be as agreed between the investigator and sponsor medical team or their designee(s). If there is a recurrence of the adverse event/lab abnormality, continued study medication dosing and follow-up should be determined (by the investigator) in conjunction with the sponsor medical team or their designee(s). Patients should resume the normal study visit schedule following the above specified monitoring period.
2. Patient will re-start study medication taking open-label 10 mg atorvastatin. For laboratory abnormality related rechallenges, follow-up labs will be done at 6 weeks post-restart. If all parameters are at satisfactory levels, the patient will stop open-label 10 mg atorvastatin and begin taking 1 tablet from bottle A and 1 tablet from bottle B. Follow-up labs will again be performed at 6 weeks following increasing the dose. If all laboratory parameters are still at satisfactory levels, the patient will be instructed to increase the dose to 1 tablet from bottle A and 2 tablets from bottle B. Repeat labs will once again be performed 6 and 12 weeks after increasing the dose. For non-laboratory abnormality related rechallenges, follow-up visits should be as agreed between the investigator and the sponsor medical team or their designee(s). The patient will resume a normal study visit schedule after the above

---

[e] See Appendix E, Other Administrative and Regulatory Procedures (Section 1.7, Emergency Information)

specified monitoring period is completed. If repeat labs show elevations, or if there is recurrence of the clinical adverse event, continued study medication dosing and follow-up should be determined (by investigator) in conjunction with the sponsor medical team or their designee(s).

Deviations from the procedure outlined above will only be allowed after discussion and approval with the sponsor medical team or their designee(s) and, if necessary, approval of the site's IRB/EC. The Individual patient rechallenge plan will be documented (by the site) in the patient's medical record.

6.4.3. Other Safety Monitoring

In addition to the recording of adverse events, the following safety assessments will be performed throughout the study in order to document and fully describe any deleterious findings:

- Physical examinations;
- Laboratory evaluations (serum chemistry/hematology), including urinalysis; and,
- Electrocardiograms.

The schedule for these safety assessments to be performed at various clinic visits during the study is provided in Appendix A. Detailed descriptions of these safety assessments are described in Appendix C.

A special safety assessment will include the comparison of noncardiovascular mortality among the treatment groups.

6.4.4. Data and Safety Monitoring Board (DSMB) UNBLINDED

An independent safety data monitoring committee, not otherwise involved in the conduct of the trial, will be the primary data and safety advisory group for the Sponsors. The DSMB will periodically review study results, evaluate the treatments for excess adverse effects, determine whether the basic trial assumptions remain valid, judge whether the overall integrity and conduct of the trial remain acceptable, and make recommendations to the Steering Committee. The Steering Committee has the responsibility to accept, reject, or modify DSMB recommendations.

The DSMB will convene prior to initiation of the trial. Thereafter, the frequency of scheduled meetings depends on patient enrollment and safety event rates. However, a scheduled meeting will convene in a single location at a minimum of once yearly.

Summary notes will be prepared for each meeting of the DSMB, distributed in a timely manner after each meeting, and reviewed and approved at the subsequent meeting. With the expectation that the Steering Committee and its chairman are blinded as to study treatments and with the expectation that the summary notes will contain some data by treatment groups (even with treatments designated by code), the summary notes will not be forwarded to the Steering Committee chairman or the Sponsor. At the end of the study and after treatment is unblinded, a copy of the summary notes will be forwarded to the Sponsors and the Steering Committee.

Meetings will consist of open and closed portions. During the initial open portion of a meeting, the Sponsor (two designees) and Steering Committee chairman may be invited to make brief presentations and be available for questions from the DSMB members. A separate charter will document specific policies and procedures of the DSMB.

6.4.5. Steering Committee

A Steering Committee will be convened that will be responsible for providing overall guidance to the sponsors on the study protocol, the scientific merit of proposed substudies, the execution of the trial, the review of recommendations from the DSMB and the oversight of study publications. A separate charter will document specific policies and procedures of the Steering Committee.

6.5. Economic Evaluation

Following completion of the data collection, health care resources will be estimated in 3 categories to conduct the cost analyses. First, the data collected on health care outcomes (hospitalizations and revascularization procedures) will be summarized. Second, the resources needed to provide lipid lowering treatment (including drugs, physician visits and laboratory monitoring) will be estimated from this trial, the literature, and product labeling. Third, resources needed to provide care for adverse events associated with treatment will be estimated from this trial and the literature.

Market-specific costs for the resources described above will be applied to determine the cost of care (including adverse events) and the costs of care of all cardiovascular outcomes.

Several economic analyses will be performed. These include a description of healthcare resource utilization, net cost analysis (the costs of providing lipid-regulating therapy minus the cost of care for healthcare outcomes) and cost-effectiveness analysis (cost per year of life saved).

June 13, 2003 – Amendment 4

6.6 Patient Follow-up/Discontinuation of Study Drug

Every effort within the bounds of safety and patient choice should be made to have patients complete the study while remaining on study drug. Regardless of their study drug status, all randomized patients should be followed for clinical end points until study completion. Patients are not to be withdrawn from the study drug or followup if they experience a non-fatal clinical end point.

Once randomized, patients should not be discontinued from study drug for noncompliance; they should, however, be counseled on the importance of regularly taking their medication. In general, patients will be encouraged to complete the study and its follow-up for clinical end points, although they may withdraw at any time without prejudice.

Consideration should be given to temporarily withholding or discontinuing the study

drug in any patient with a risk factor or concomitant medication predisposing to the development of renal failure secondary to rhabdomyolysis, including severe acute infection, hypotension, major surgery, trauma, severe metabolic, endocrine, or electrolyte disorders, and uncontrolled seizures.

If a patient becomes pregnant during the trial, study drug should be discontinued and the patient apprised of the potential hazard to the fetus.

Patients prematurely discontinued from study drug during the double-blind phase should have all end-of-study procedures performed (see annual visits beginning at month 24) and may then return to non-protocol lipid therapy. If the final lipid measurements (lipid profile) cannot be performed within 3 days of the cessation of therapy, these measurements should not be performed at all. In this case, the previously determined lipid values will be carried forward for the purpose of analysis.

For patients who withdraw from the double-blind period early, an effort will be made to collect full follow-up information on primary and secondary clinical end points until such time that the study is completed. Methods of follow-up for these patients should include clinic visits when possible according to the timetable (Appendix A) but telephone or mail follow-up should be attempted as well when visits are not possible.

If necessary, an independent search firm will be contracted to locate patients who are lost-to-follow-up at the end of the study to determine if the patient is alive or deceased.

In order to validly assess the safety and efficacy of the two treatment groups, complete follow-up of all randomized patients is critical. At a minimum, the vital status of every randomized patient must be determined upon study completion. Within local laws and regulations, all sources of data (direct patient follow-up, telephone contact, mail questionnaires, and public sources of information will be utilized in order to ascertain the vital status of each randomized patient.

Clearly indicate on the appropriate CRF the reason for withdrawal from the study.

6.7. Study Completion[f]

<u>June 13, 2003 – Amendment 4</u>

Unless the study is stopped early as described in Section 9.6.2, patients will complete their participation in the study when the number of ***major coronary events (CHD death, non-fatal myocardial infarction, or resuscitated cardiac arrest)*** is achieved (n = 750) ***<letter/sentence/paragraph deleted>***, at which time all patients will have a final annual visit ***(if an annual visit has not been completed in the past 3 months). Subjects not requiring a final clinic visit will be contacted for a final study follow-up/non-clinic visit.*** Clearly indicate on the appropriate CRF the completion date for each patient.

---

[f] See Appendix E, Other Administrative and Regulatory Procedures (Section 3.3, Study Termination)

7. STUDY MEDICATION[g]

7.1 Formulation

June 13, 2003 – Amendment 4

Pfizer Pharmacy Operations or their designee will provide medication assembled for each patient based on a randomization code prepared by the Biometrics Department (or their designee).

Each blinded medication container will have a portion of the label that is to separated from the bottle and attached to the appropriate CRF when the medication is dispensed. Open-label run-in medication will be provided as follows:

	All Patients
Run-in bottle:	atorvastatin 10 mg

Double-blind medication will be provided in a double-dummy format as follows:

	Atorvastatin 10 mg Group	Atorvastatin 80 mg Group
bottle A:	atorvastatin 10 mg	placebo for atorvastatin 10 mg
bottle B:	placebo for atorvastatin 40 mg	atorvastatin 40 mg tablets

7.2. Medication Dispensing

Study medication is to be dispensed by the investigator or the designated hospital pharmacy according to a detailed set of dispensing instructions supplied with the initial shipment of study medication. One bottle of medication must be dispensed at Visit 2 for the atorvastatin 10 mg run-in period and at least two bottles at each visit during the double-blind treatment period. The patient should be instructed to return medication bottles at the next visit and not combine medication from different bottles at any time.

7.3. Dosage Regimen

During the open-label run-in atorvastatin period patients will be taking 10 mg once daily and during the double-blind treatment period patients will be taking either 80 mg atorvastatin or 10 mg of atorvastatin once daily. Patients should take 1 tablet daily during the run-in period. During the double-blind treatment period, patients should take 1 tablet from bottle A and 2 tablets from bottle B once daily. Dosing can be at anytime of the day, with or without food, but should be consistent throughout the study.

June 13, 2003 – Amendment 4

8. DATA COLLECTION

CRFs for all patients will be supplied by ***<letter, sentence, paragraph deleted>*** Pfizer, or their designees. These are to be completed as instructed.[h] Original source documents,

---

[g] See Appendix E, Other Administrative and Regulatory Procedures (Sections 1.7.2, Medication Code Card and 2.4, Investigational Drug Supply Accountability)

[h] See Appendix E, Other Administrative and Regulatory Procedures (Section 2.2, Guidelines for Recording Data in the Case Report Forms)

CRFs, and other study documents must be maintained at the site as specified.[i] Regular monitoring will be performed by ***<letter, sentence, paragraph deleted>*** Pfizer, or their designees.[j] CRFs will be collected at regular intervals.

9. DATA ANALYSIS AND STATISTICAL CONSIDERATIONS

June 13, 2003 – Amendment 4

9.1. Sample Size Considerations

Assuming a Visit 2 distribution of LDL-C as observed in NHANES III for persons with a history of coronary disease and in the protocol range of 130-250 mg/dl (3.4-6.5 mmol/l), estimated baseline and on-trial levels are as follows:

	Atorvastatin 10 mg	Atorvastatin 80 mg
Mean Visit 2 LDL-C	163 mg/dl	163 mg/dl
Atorvastatin 10 mg Run-In	-35% ± 12%	-35% ± 12%
Mean Visits 3 and 4 LDL-C	106 mg/dl	106 mg/dl
Estimated % > 130 mg/dl (3.4 mmol/l) @ Visits 3/4 (excluded at Visit 5)	16%	16%
Post-randomization Reduction in LDL-C	-	-55% from Visit 2
On-Trial LDL-C	101 mg/dl	73 mg/dl
Difference	-28 mg/dl or 0.7 mmol/l (total and LDL-C)	

Using various reported analyses of the relationship between total cholesterol and CHD occurrence [34-37], the above differences are estimated to translate to a 20-31% reduction in CHD. A 22% estimate (discounting any effect of triglycerides) is assumed. Finally, a 1.9% annual incidence is assumed in the atorvastatin 10 mg group or approximately 9% over a 5 year treatment duration. This is based on a 28% reduction in the placebo group primary endpoint reported in the CARE trial [8].

<Letter, sentence, paragraph deleted>

---

[i] See Appendix E, Other Administrative and Regulatory Procedures (Section 2.1.4, Retention of Study Records)

[j] See Appendix E, Other Administrative and Regulatory Procedures (Sections 1.4, Monitoring the Study and 2.3, Review of Case Report Forms)

Assuming a 5 year cumulative ***major coronary*** event rate in the atorvastatin 80 mg group of 7.02%, a 5 year cumulative ***major coronary*** event rate in the atorvastatin 10 mg group of 9%, and no dropouts, drop-ins, or noncompliance, a sample size of 6,928 subjects would have 91% power for a one-sided test at alpha of 0.05 and 85% power for a two-sided test at alpha of 0.05. In order to compensate for an estimated 10% dropout/drop-in/noncomplier rate, the study will have a target enrollment of approximately 8,600 subjects [$6,928/(1-0.1)^2 = 8,553$] ***in order to accumulate the target number of 750 major coronary events in an average follow-up time estimated at 5.5 years*** (Lachin, JM, *Controlled Clinical Trials* 2, 93-113 (1981)).

The sample size estimation shown above was based on assumptions regarding incidence rates for major coronary events (the primary endpoint excluding stroke). It is anticipated that approximately 950 primary endpoints (an additional 200 stroke events) will accrue over the total expected duration of the trial, thereby providing 91% power for a one-sided test at alpha of 0.05 and 85% power for a two-sided test at alpha of 0.05 to detect a 17% reduction in the 5 year cumulative primary endpoint rate for atorvastatin 80 mg relative to atorvastatin 10 mg.

<letter, sentence, paragraph deleted> The power calculations were performed using n-Query Advisor, Version 2.0, by Janet D. Elashoff.

The steering committee will examine blinded incidence, dropout rate, and other key assumption information when approximately 2 years of completed follow-up data is available and decide whether the proposed target number of events (described in Section 6.1) is appropriate. The sponsor will be approached if a change is proposed.

June 13, 2003 – Amendment 4

9.2. Analysis Populations

The primary efficacy ***and safety*** population will consist of all randomized subjects who ***were dispensed*** at least one dose of blinded study drug ***<letter, sentence, paragraph deleted>*** irrespective of any ***<letter, sentence, paragraph deleted>*** deviations from the protocol or premature discontinuation.

<Letter, sentence, paragraph deleted>.

June 13, 2003 – Amendment 4

9.3. Efficacy Parameters

June 13, 2003 – Amendment 4

9.3.1. Primary Efficacy: Time to the Occurrence of Major Cardiovascular Events

<Letter, sentence, paragraph deleted>The time to occurrence of a major cardiovascular event (CHD death, non-fatal myocardial infarction, resuscitated cardiac arrest, fatal or non-fatal stroke) is defined as the period of time elapsing between the first day of blinded study drug dispensed and the first day of documented occurrence of the major event.

June 13, 2003 – Amendment 4

<Letter, sentence, paragraph deleted from 9.3.2 thru 9.5.4.2>

9.3.2. Secondary Efficacy: Definitions

9.3.2.1 Other Cardiovascular Morbidity and Mortality

The time to occurrence of the following events will be calculated as the period of time elapsing between the first day of blinded study drug dispensed and the first day of documented occurrence of:

a. ***Major coronary event (CHD death, non-fatal myocardial infarction, or resuscitated cardiac arrest).***
b. ***Any coronary event (major coronary event or, CABG, PTCA, other revascularization procedure, procedure-related myocardial infarction or documented angina)***
c. ***cerebrovascular event (fatal or non-fatal stroke, TIA)***
d. ***peripheral vascular disease (PVD)***
e. ***hospitalization with primary diagnosis of CHF***
f. ***any cardiovascular event (any of the above)***

9.3.2.2 All Cause Mortality

The time to occurrence of all cause mortality will be calculated as the period of time elapsing between the first day of blinded study drug dispensed and the day of documented occurrence of death.

9.4. Methods of Analysis

All hypothesis testing will be two-sided at a significance level of 0.05 unless otherwise indicated.

Subjects who discontinue study medication will continue to be followed to assess the time to occurrence of each endpoint. Subjects who do not experience endpoints during the trial will be considered censored at the time of the data cut-off, death or last contact, whichever is earliest.

9.4.1. Primary Efficacy Analysis

The null hypothesis that there is no difference in the time to occurrence of a major cardiovascular event between the two treatment groups will be examined using a log rank test. The product limit estimates for the time to occurrence will be calculated and displayed graphically as "survival" curves.

9.4.2. Secondary Efficacy Analysis

A log rank test will be used to examine the differences in time to occurrence between the two treatment groups for the other cardiovascular morbidity and mortality variables and all cause mortality.

Risk ratios and 95% confidence limits will be estimated for the primary and secondary efficacy endpoints using Cox regression.

9.4.3. Covariate and Subgroup Analyses

Cox regression models will be used to examine the treatment effect adjusted for various covariates, and to examine the treatment by covariate interaction effects. Covariates to be examined include age, gender smoking status, presence or absence of diabetes, baseline triglycerides, baseline HDL, baseline LDL, CHF, other prevalent pre-existing condition categories of the study cohort, and prevalent concomitant medication use (such as hormone replacement therapy, aspirin, beta-blockers, calcium channel blockers, etc.). In addition, the above analyses will be used to examine subgroups of interest as specified above, such as women and revascularized patients.

9.5. Safety Parameters

Adverse events will be coded and grouped by body system. The incidence of adverse events reported during the study will be tabulated according to the number of affected patients as well as the number of events. The intensity and relationship to study drug of adverse events will also be summarized by body system and treatment groups.

The incidence of shifts from normal at baseline to abnormal at study completion will be summarized both overall and by direction for all safety laboratories by treatment group.

In addition, changes in serum lipid levels after 1, 2,3,4 and 5 years of treatment will be calculated for each treatment group both as percent change from baseline (the average of the values at Visit 4 and Visit 5) and as percent change from Visit 2. Serum lipids include total cholesterol, LDL, HDL, triglycerides, apolipoproteins A1 and B, and non-HDL. Further analysis to determine the relationship between the occurance of endpoints and these lipid parameters will be investigated.

9.6. Interim Analyses

June 13, 2003 – Amendment 4

9.6.1. Timing, Content

Interim analyses to examine ***primary*** efficacy ***<letter, sentence, paragraph deleted>*** will ***begin after a median of 3 years of follow-up. The timing and number of interim analyses will be at the discretion of the DSMB.***

The interim analyses will include analyses of recruitment, subject characteristics, the primary efficacy and safety variables, all-cause mortality, and adverse events.

June 13, 2003 – Amendment 4

9.6.2. Early stopping

The ***log-rank*** statistic for the hypothesis test of the null hypothesis of no difference in the probability of occurrence of major ***cardiovascular*** events between atorvastatin 10 and 80 mg groups will be compared to the critical boundary values ***based on a two-sided Peto type monitoring boundary with a critical value (Z) of*** $\pm$ ***3.0.*** If the group sequential boundary is crossed, then the DSMB will consider a recommendation to stop the trial.

However, actual stopping of the trial is a complex decision involving a number of factors besides the simple comparison of one statistic with one critical value. The DSMB will take all the data together and analyze the data in concert including performing additional analyses before making a recommendation that the trial be stopped. A partial list of factors besides the magnitude of an apparent treatment effect that should be taken into account include the clinical significance of an apparent effect, whether an apparent effect could be explained by baseline imbalance or concomitant therapy, whether occurrence of major coronary events is consistent with other signals in the data, whether results are consistent within subgroups and centers, the likelihood of a reversal in the current trend, and the needs of the medical research community and future patients for a definitive answer to the research questions.

Interim monitoring for harm will be based on all-cause mortality using a log-rank test with an O'Brien-Fleming boundary (two-sided $\alpha = .05$) truncated to ± 3.0. If the group sequential boundary is crossed, then the DSMB will consider a recommendation to stop the trial. The DSMB will take all the data together and analyze the data in concert including performing additional analyses before making a recommendation that the trial be stopped for harm.

In summary, a recommendation to modify or terminate the trial would not be based totally on the crossing of a group-sequential boundary.

<Letter, sentence, paragraph deleted>

9.6.3. Distribution of the interim analysis

A limited number of copies of the report containing the interim analyses will be produced. They will be numbered, and they will be collected after each meeting. Only those individuals on the DSMB will have access to the results.

10. ECONOMIC ANALYSES

Descriptive summaries of health care outcomes will be reported. These summaries include but are not limited to: rate of CV hospitalizations and revascularization procedures, total number of hospital days, time to first hospitalization and time to first revascularization procedure.

11. DNA Databank

11.1 INTRODUCTION

New developments in molecular biology have directed research of human disease towards its genetic basis. The understanding of the molecular origin of a disorder and the insight into the way genetic information is expressed or modulated by the environment will have a major impact on treatment and prevention of disease. It is estimated that the genetic predisposition for atherosclerotic disease involves more than 200 genes. The exact role in the atherosclerotic process is only known for a small number of genes. These genes play an important role in the aetiology of atherosclerotic disorders such as coronary heart disease (CHD), as has been shown in a large number of studies. The use of large, well documented

patient cohorts has enabled the identification of new genetic risk factors for CHD. The aim of the DNA databank is to establish the relationship between a number of genetic markers, known to influence cardiovascular risk in the general population, and the clinical phenotype and response to lipid lowering therapy in patients with pre-existing cardiovascular disease.

June 13, 2003 – Amendment 4
11.2. PROCEDURE
At Visit 5 or the first possible visit at which blood is collected post-randomization, 12 mls of whole blood will be collected into plastic tubes containing EDTA from randomized subjects who consent to undergo additional genetic testing. Refusal to participate in the additional testing will not effect the subject's participation in the main study. After collection and labeling of the sample, the whole blood sample with the other biochemical samples collected during this visit will be mailed to:

[redacted] or [redacted]
ATTN: [redacted]
[redacted]

11.3. PROCESS FOR ANONYMITY OF BLOOD SAMPLES

Prior to transfer to the genetics laboratory that will perform the DNA analyses, the blood sample will be relabeled with an anonymous bar code label which contains no patient identifying information. A sample registration form which contains an identical bar code will be used to record the patient entry/screening number, patient randomization number, and date of collection. The registration form is mailed separately to an administrator and held secured until the patient data is transferred to the disease database as described below.

Demographic and disease-related characteristics of subjects in this study who agree to participate in genetic testing will be extracted from the database and moved to a separate Disease Database. This process will not alter the protocol-specific database in any way. For data copied into the Disease Database, the subject identifying information will be removed and the demographic, disease information, and their lipid profile will only be linked to samples collected. Results of such tests will be kept confidential and will not be available to investigators or subjects. Samples and disease information will be used to help define disease characteristics that are associated with specific genetic and biochemical markers. When the study is completed, the secure administrator will populate the Disease Database with the genotype data, biochemical data, disease information, but no patient identifiers will be transferred. The genetic sample registration form will then be destroyed.

12. REFERENCES

1. Goldman L. Cholesterol Reduction. In, Prevention of Myocardial Infarction. JE Manson, PM Ridker, JM Gaziano, CH Hennekens, eds. New York: Oxford U. Press, 1996.

2. Breslow JL, Plump A, Dammerman M. New Mouse Models of Lipoprotein Disorders and Atherosclerosis. In, Atherosclerosis and Coronary Heart Disease. V Fuster, R Ross, E Topel, eds. New York: Lippincott-Raven, 1996.

3. Verschuren WM, Jacobs D, Bloemberg BP, et al. Serum total cholesterol and long-term coronary heart disease in different countries: twenty-five-year follow-up of the Seven Countries Study. JAMA 1995; 274:131-136.

4. Stamler J, Wentworth D, Neaton JD. Is the relationship between serum cholesterol and risk of premature death from coronary heart disease continuous or graded. Findings in 356,222 primary screenees of the Multiple Risk Factor Intervention Trial (MRFIT). JAMA 1986;256:2823-28.

5. Gordon T, Kannel WB, Castelli WP, Dawber TR. Lipoproteins, cardiovascular disease and death: the Framingham study. Arch Intern Med 1981;141:1128-1131.

6. Chen Z, Peto R, Collins R, MacMahon S, Lu J, Li W. Serum cholesterol concentration and coronary heart disease in a population with low cholesterol concentrations. BMJ 1991;303:276-282.

7. Scandinavian Simvastatin Survival Study Group. Randomized trial of cholesterol lowering in 4444 patients with coronary heart disease: the Scandinavian Simvastatin Survival Study Group. Lancet 1994;344:1383-1389.

8. Sachs FM, Pfeffer MA, Maye LA, et al. for the Cholesterol and Recurrent Events Trial Investigators. The effect of pravastatin on coronary events after myocardial infarction in patients with average cholesterol levels. N Engl J Med 1996;335:1001-1009.

9. Shepherd J, Cobbe SM, Ford I, et al. Prevention of coronary heart disease with pravastatin in men with hypercholesterolemia. N Engl J Med 1995;333:1301-1307.

10. Gotto AM, Jr. AFCAPS/TexCAPS Trial. Preliminary results presented at the 70th Scientific Sessions of the American Heart Association. Orlando, Florida; November 1997.

11. Expert Panel on Detection, Evaluation and Treatment of High Blood Cholesterol In Adults. Summary of the second report of the National Cholesterol Education Program (NCEP) Expert Panel on detection, evaluation and treatment of high blood cholesterol in adults (Adult Treatment Panel II). JAMA 1993;269:3015-3023.

12. Lewis B, Assmann G, Tikkanen M, et al. Prevention of coronary heart disease: scientific background and new clinical guidelines. Recommendations of the European Atherosclerosis Society prepared by the International Task Force for Prevention of Coronary Heart Disease. Nutr Metab Cardiocasc Dis 1992;2:113-156.

13. Pyorala K, DeBacker G, Graham I, et al. on behalf of the Task Force. Prevention of coronary heart disease in clinical practice. Recommendations of the Task Force of the European Society of Cardiology, European Atherosclerosis Society and European Society of Hypertension. Eur Heart J 1994;15:1300-1331.

14. International Task Force For The Prevention of Coronary Heart Disease. Guidelines of the European Atherosclerosis Society. A desktop guide to the management of risk factors for coronary heart disease. London: Current Medical Literature Ltd. (date unknown).

15. The Scandinavian Simvastatin Survival Study Group: design and baseline results of the Scandinavian Simvastatin Survival Study of patients with stable angina and/or previous myocardial infarction. Am J Cardiol 1993;71:393-400.

16. Kjekshus J, Pedersen TR for the Scandanavian Simvastatin Survival Study Group. Reducing the risk of coronary events: evidence from the Scandinavian Simvastatin Survival Study (4S). Am J Cardiol 1995;76:64c-68c.

17. Pyorala K, Pedersen TR, Kjekshus J, Faergeman O, Olsson AG, Thorgiersson G for the Scandanavian Simvastatin Survival Study (4S) Group. Cholesterol lowering with simvastatin improves prognosis of diabetic patients with coronary heart disease. Diabetes Care 1997;20:614-620.

18. Scandanavian Simvastatain Survival Study Group. Baseline serum cholesterol and treatment effect in the Scandanavian Simvastatin Survival Study (4S). Lancet 1995;345:1274-1275.

19. Goa K, Barradell L and McTavish D. Simvastatin. A reappraisal of its cost effectiveness in dyslipidemia and coronary heart disease. PharmacoEconomics 1997;11(1):89-110.

20. Pedersen T, Kjekshus J, Berg K, et al. Cholesterol lowering and the use of healthcare resources: Results of the Scandinavian Simvastatin Survival Study. Circulation 1996; 93(10):1796-1802.

21. Jonsson B, Johannesson M, Kjekshus J, et al. Cost-effectiveness of cholesterol lowering. Results from the Scandinavian Simvastatin Survival Study (4S). Eur Heart J 1996;17:1001-1007.

22. Johannesson M, Jonsson B, et al. Cost-effectiveness of simvastatin treatment to lower cholesterol levels in patients with coronary heart disease. N Engl J Med 1997;336(5):332-336.

23 Sachs FM, Pfeffer MA, Moye L, Brown LE, Hamm P, Cole TG, Hawkins CM, Braunwald E for the CARE Investigators. Rationale and design for a secondary prevention trial of lowering normal plasma cholesterol levels after acute myocardial infarction: The Cholesterol and Recurrent Events trial (CARE). Am J Cardiol 1991;68:1436-1446.

24. The Long-Term Intervention with Pravastatin in Ischemic Disease (LIPID) Study Group. Prevention of cardiovascular events and death with pravastatin in patients with coronary heart disease and a broad range of initial cholesterol levels. N Engl J Med 1998; 339:1349-1357.

25. The Post Coronary Artery Bypass Graft Trial Investigators. The effect of aggressive lowering of low-density lipoprotein cholesterol levels and low-dose anticoagulation on obstructive changes in saphenous-vein coronary-artery bypass grafts. N Engl J Med 1997;336:153-162.

26. Thompson GR, Hollyer J, Waters DD. Percentage rather than plasma level of LDL-cholesterol determines therapeutic response in coronary heart disease. Curr Opin Lipidol 1995;6:386-388.

27. Herd JA, Ballantyne CM, Farmer JA, et al. Effects of fluvastatin on coronary atherosclerosis in patients with mild to moderate cholesterol elevations (Lipoprotein and Coronary Atherosclerosis Study [LCAS]). Am J Cardiol 1997;80:278-286.

28. Roberts WC. Lipid-lowering therapy after an atherosclerotic event. Am J Cardiol 1989;64:693-695.

29. Lea AP and McTavish D. Atorvastatin. A review of its pharmacology and therapeutic potential in the management of hyperlipidemias. Drugs 1997;53:828-847.

30. Davidson M, McKenney J, Stein E, et al. Comparison of one-year efficacy and safety of atorvastatin versus lovastatin in primary hypercholesterolemia. Am J Cardiol 1997;79:1475-1481.

31. Bertolini S, Bon GB, Campbell LM, et al. Efficacy and safety of atorvastatin compared to pravastatin in patients with hypercholesterolemia. Atherosclerosis 1997;130:191-197.

32. Dart A, Jerums G, Nicholson G, et al. A multicenter, double-blind, one-year study comparing safety and efficacy of atorvastatin versus simvastatin in patients with hypercholesterolemia. Am J Cardiol 1997;80:39-44.

33. Heinonen T and Black D. Atorvastatin monotherapy and combination therapy in the treatment of severe hypercholesterolemia [abstract]. Atherosclerosis 1995;115(suppl): s20.

34. Law MR, Wald NJ, Thompson SG. By how much and how quickly does reduction in serum cholesterol concentration lower risk of ischemic heart disease. BMJ 1994;308:367-372.

35. Holme I. An analysis of randomized trials evaluating the effect of cholesterol reduction on total mortality and coronary heart disease incidence. Circulation 1990;82:1916-1924.

36. Gould AL, Rossouw JE, Santanello NC, Heyse JF, Furberg CD. Cholesterol reduction yields clinical benefit. A new look at old data. Circulation 1995;91:2274-2282.

37. Assman G, Schulte H, von Eckardstein A . Hypertriglyceridemia and elevated lipoprotein (a) are risk factors for major coronary events in middle-aged men. Am J Cardiol 1996;77:1179-1184.

13. LIST OF APPENDICES

**Appendix**	**Title**
A	Schedule and Description of Study Procedures
B	Commonly Prescribed Prohibited Medications
C	Study Safety Procedures and Clinical Laboratory Determinations
D	Administrative Procedures for the Reporting of Adverse Events
E	Other Administrative and Regulatory Procedures
F	Declaration of Helsinki
G	Amendments - October 6, 1998
H	Amendments – January 15, 1999
I	Amendments – June 4, 1999
J	***Amendments – June 13, 2003***

APPENDIX A
TIMETABLE OF VISITS AND PROCEDURES

APPENDIX A. 1
TIMETABLE OF CLINIC VISITS AND PROCEDURES

Study Period	Screening	Atorvastatin 10 mg			Double-Blind Treatment						
**Visit #**	**1**	**2**	**3**	**4**	**5**	**6**	**7**	**8**	**9**	**10,12,14,16,18[i]**	**11,13,15,17,19[i]**
study week		wk -8	wk -4	wk -2	wk 0	wk 12					
study month							mo 6	mo 9	mo 12	mos. 18, 30, 42,54, 66	mos. 24, 36, 48, 60, 72
PROCEDURE											
Informed Consent	X										
Demographics/ Med. History	X										
Vital Signs[a]	X	X	X	X	X	X	X	X	X	X	X
Clinical Labs[b,c,k]	X				X				X		X
Lipid Labs[d,k]	X	X	X	X	X[e]	X			X[e]		X
Safety Labs[f,k]				X		X					
Dietary Counseling	X----	--throughout trial--									----------X
Physical Exam				X[g]----	----X[g]				X		X
ECG				X[g]----	----X[g]				X		X
Dispense Drug		X[h]			X	X	X	X	X	X	X
Concom. Therapy	X	X	X	X	X	X	X	X	X	X	X
Adverse Events		X	X	X	X	X	X	X	X	X	X
End Points[j]		X	X	X	X	X	X	X	X	X	X

[a] height (screen only), weight, blood pressure
[b] serum chemistry and hematology by central laboratory and uninalysis at local site (see Appendix C.4.)
[c] includes TSH, pregnancy test (women of child-bearing potential), and HbA1C at screening; pregnancy testing (women of child-bearing potential) and the HbA1C annually (known/suspected diabetics only) beginning at Visit 9; No Pregnancy Test or HbA1C will be performed at Visit 5. Additional plasma will be stored at Visits 5, 9, and annually thereafter.
[d] includes total cholesterol, triglycerides, LDL-C, HDL-C
[e] includes apolipoprotein A1, B
[f] includes transaminases (ALT, AST) and CPK if clinically indicated
[g] can be done between Visit 4 or 5 once a patient's lipid levels qualify them for randomization
[h] open label atorvastatin 10 mg
[i] the semi-annual visit schedule beginning with Visits 10 and 11 will extend beyond month 72 for patients beyond this time point if the trial has not yet been stopped
[j]. includes resource utilization for economic analyses
k. one 12 mls whole blood will be collected (at Visit 5 or the first available post-randomization visit) for the DNA databank

APPENDIX A.2
STUDY PROCEDURES BY VISIT

1. SCREENING VISIT

Perform the following procedures at the single screening visit:

Visit 1
- Obtain signed informed consent
- Obtain Medical History
- Begin to assess the patient's eligibility for the study considering the protocol inclusion/exclusion criteria such as presence of CHD, history of previous myocardial infarction, age, etc.
- Draw blood samples for a complete clinical laboratory determination (including HbA_{1c}), lipid profile, and hematology
- Vital signs
- Urinalysis
- Begin dietary counseling
- Concurrent medication
- Discontinue prior dyslipidemic medication (if applicable)
- Pregnancy test for women of child-bearing potential

2. ATORVASTATIN 10 MG PERIOD (OPEN)

Perform the following procedures during the visits of the open-label run-in period:

Visit 2-3
- Draw blood sample for lipid profile
- Vital signs
- Dietary counseling
- Dispense study medication (atorvastatin 10 mg run-in at Visit 2 only)
- Record potential end points
- Record adverse events
- Record changes in and additions to concurrent medications

Visit 4
- Perform physical examination (or at visit 5)
- ECG (or at visit 5)
- Draw blood sample for safety laboratory and lipid profile
- Vital signs
- Continue dietary counseling
- Record adverse events
- Record potential end points
- Record changes in and additions to concurrent medications

3. DOUBLE-BLIND TREATMENT PERIOD

For patients who meet all of the inclusion criteria at the end of the prior period and who have none of the exclusion criteria and who will be randomized to double-blind treatment, perform the following procedures during the visits of the double-blind treatment period.

Visit 5

- Assign patient number
- Draw blood , DNA databank (Note: this sample will be collected at this visit or the first visit after Visit 5 where blood samples are drawn) samples for a complete clinical laboratory, lipid profile, special lipids, hematology and plasma storage
- Vital signs
- Urinalysis
- Physical Examination (if not done at Visit 4)
- Perform baseline ECG (if not done at Visit 4)
- Continue dietary counseling
- Dispense study medication
- Record adverse events
- Record potential end points
- Record changes in and additions to concurrent medications

Visits 6,7,8

- Draw blood samples for a safety laboratory (Visit 6 only) and lipid profile (Visit 6 only)
- Vital signs
- Continue dietary counseling
- Dispense study medication
- Record adverse events
- Record potential end points
- Record changes in and additions to concurrent medications

Visits 9,11,13,15,17,19 (and at 12 month intervals thereafter as required) and Study Completion

- Physical examination
- Draw blood samples for full clinical laboratory, lipid profile (special lipids at Visit 9 only), hematology, HbA1C (known/suspected diabetics), and plasma storage
- Vital signs
- Urinalysis
- ECG
- Continue dietary counseling
- Dispense study medication (not required at final visit)
- Record adverse events
- Record potential end points
- Record changes in and additions to concurrent medications
- Pregnancy test for women of child-bearing potential

Visits 10,12,14,16,18 (and at 12 month intervals thereafter as required)

- Vital signs
- Continue dietary counseling
- Dispense study medication (not required at final visit)
- Record adverse events
- Record potential end points
- Record changes in and additions to concurrent medications

APPENDIX A.3
NIH NATIONAL CHOLESTEROL EDUCATION PROGRAM DIETS

NIH NATIONAL CHOLESTEROL EDUCATION PROGRAM STEP I DIET

FAT: Less than 30% of total calories

Saturated Fats: Less than 10% of total calories

CARBOHYDRATES: 50% to 60% of total calories

PROTEIN: 10% to 20% of total calories

CHOLESTEROL: Less than 300 mg/day

TOTAL CALORIES: To achieve and maintain desirable weight

NIH NATIONAL CHOLESTEROL EDUCATION PROGRAM STEP II DIET

FAT: Less than 30% of total calories

Saturated Fats: Less than 7% of total calories
Polyunsaturated Fats: Up to 10% of total calories
Monounsaturated Fats: 10% to 15% of total calories

CARBOHYDRATES: 50% to 60% of total calories

PROTEIN: 10% to 20% of total calories

CHOLESTEROL: Less than 200 mg/day

TOTAL CALORIES: To achieve and maintain desirable weight

APPENDIX A.4
LIPID AND LIPOPROTEIN MEASUREMENTS

1. LIPID PROFILE (SERUM DETERMINATION)

Patient posture and tourniquet use should be standardized at a site. If no standardization exists at a site, the recommended procedure is as follows: patients should be in a sitting position (no longer than 5 minutes) and a tourniquet may be used (no longer than 2 minutes) but must be released prior to blood draw.

June 13, 2003 – Amendment 4

Total cholesterol
LDL-C[a] [c]
HDL-C
VLDL-C[b]
TG

[a] At all designated visits, LDL-C will be estimated by the Friedewald formula unless the TG value at that visit is >400 mg/dL (>4.5 mmol/l) at which point the LDL-C will be measured directly.

[b] VLDL-C will be calculated as total cholesterol - [HDL-C] - [LDL-C] whenever LDL-C is measured directly or will be estimated as TG/5 when the Friedewald formula is used.

June 13, 2003 – Amendment 4

c ***At all annual visits where LDL-C < 50 mg/dl (1.3 mmol/l), a direct LDL measurement will be completed. All LDL results will remain blinded.***

2. SPECIAL LIPIDS (SERUM DETERMINATION)

Apo AI
Apo B

Appendix A-5 - Objective Criteria for Atherosclerotic CHD <Appendix deleted>

APPENDIX B
COMMONLY PRESCRIBED PROHIBITED MEDICATIONS

APPENDIX B

PROHIBITED CONCOMITANT MEDICATIONS

Erythromycin

Generic Name	Market Name*	Form	Manufacturer
Erythromycin Base	E-Mycin	Tablet (Base-Enteric Coated)	Boots Laboratories
	Ery-Tab	Tablet (Base-Enteric Coated)	Abbott
	ERYC	Capsules (Base-Enteric Coated pellets)	Parke-Davis
	Erythromycin	Filmtab (Base)	Abbott
	Erythromycin Delayed Release	Capsules (Base-Enteric Coated pellets)	Abbott
	PCE Dispertab	Oral Suspension	Abbott
Erythromycin Ethyl Succinate, Acetyl Sulfisoxazole	Pediazole	Oral Suspension	Ross
Clarithromycin	Biaxin	Tablet, granules	Abbot

Immunosuppressive Agents

Generic Name	Market Name*	Form/Source	Manufacturer
Lymphocyte Immune Globulin	Atgom	Sterile Injection/Horse	Upjohn
Rho(D) Immune Globulin	HypRho-D<br>Rho-D	Sterile Injection/Human	Miles-Cutter
	MICRhoGAM<br>Rohm	Sterile Injection/Human	Ortho Diagnostics
Azathiopane Sodium	Imuran	Sterile Solution<br>Tablets	Burroughs<br>Wellcome
Muromonab-CD3	Ortoclone OKT3	Sterile Injection	Ortho Biotech
Cyclosporine	Sandimmune	Capsule<br>Oral Solution<br>IV Amps for injection	Sandoz Pharmaceuticals

Lipid Regulating Drugs

Generic Name	Market Name*	Form/Source	Manufacturer
Niacin/Nicotinic Acid	Niacor	Tablet	Upsher-Smith
	Nicobid	Capsule (Time-Release)	Rhone-Poulenc Rorer Pharmaceuticals
	Nicolar	Tablet	Rhone-Poulenc Rorer Pharmaceuticals
	Slo-Niacin	Tablet	Rhone-Poulenc Rorer Pharmaceuticals
Probucol	Lorelco	Tablet	Marion Merrell Dow
Clofibrate	Atromid-5	Capsule	Wyeth-Ayerst
Cholestyramine	Cholybar	Bar	Parke-Davis
	Questran	Powder Packet/Can	Bristol Laboratories
	Questran (Light)		
Colestipol Hydrochloride	Colestid	Packet	Upjohn
Gemfibrozil	Lopid	Capsules	Parke-Davis
Fluvastatin	Lescol	Tablet	Sandoz
Lovastatin	Mevacor	Tablet	Merck, Sharp, Dohme
Pravastatin Sodium	Pravachol	Tablet	Squibb
Simvastatin	Zocor	Tablet	Merk, Sharp, Dohme
Cerivastatin	Baychol	Tablet	Bayer

The following are also prohibited:

- any chronic systemic anti-fungal agent use
- mibefradil dihydrochloride (Posicor®)
- fish oils (prescription only)
- nefazodone
- fluvoxamine
- concurrent use of other potent cytochrome P450-3A4 inhibitors

* Market Names represent examples of some brand names but is not intended to be a complete listing of all the names that these products are marketed under.

APPENDIX C
STUDY SAFETY PROCEDURES AND CLINICAL LABORATORY DETERMINATIONS

APPENDIX C.1
MEDICAL HISTORY AND PHYSICAL EXAMINATIONS

1. MEDICAL HISTORY

The following elements of medical history will be recorded:

- Age;
- Significant medical history;
- Prior dyslipidemic medications (discontinued at or prior to screening);
- Any adverse reactions to medications.

2. PHYSICAL EXAMINATION

Physical examination will include the following:

- Weight, height, skin lipid abnormalities and general appearance;
- Cardiovascular assessment including blood pressures, rhythm, and presence of other cardiac abnormalities (i.e., gallops, murmurs, cardiomegaly) and bruits;
- Respiratory examination to document the presence of auscultatory abnormalities;
- Abdominal examination to document the presence of abnormalities;
- Neurologic examination to record the presence of abnormalities in mental status, motor, and sensory function;
- Eye examination including eye movements, pupillary response, nystagmus, fundoscopic examination; and,
- Any additional assessments necessary to establish baseline status (e.g., peripheral pulses)or evaluate symptoms or adverse experiences.

APPENDIX C.2
ELECTROCARDIOGRAM

A 12-lead electrocardiogram will be recorded as scheduled with the patient recumbent and resting. The electrocardiogram should be performed and submitted to [redacted]e [redacted]:s [redacted]) using the procedure specified for routine ECGs.

If a patient experiences a myocardial infarction after randomization, a new baseline ECG should be performed between 6 weeks and 3 months following the myocardial infarction. It may be done at a regularly scheduled visit if the visit is to occur within that time frame. If not, a new baseline ECG should be performed at an unscheduled study visit. The ECG should be performed and submitted to CCD using the procedure specified for routine ECGs.

APPENDIX C.3
ROUTINE SAFETY PROFILE

At each clinic visit the following minimum safety data will be collected: blood pressure, and weight.

APPENDIX C.4
CLINICAL LABORATORY PARAMETERS

1. FULL CLINICAL LABORATORY (central laboratory)

Hematology
- RBC
- Hemoglobin
- Hematocrit
- WBC
- Platelet Count
- Differential
- (if WBC is abnormal)
  - Neutrophils
  - Lymphocytes
  - Monocytes
  - Eosinophils
  - Basophils

Chemistry
- SGOT (AST)
- SGPT (ALT)
- Alkaline Phosphatase
- LDH
- CPK (Troponin-T if CPK elevated above 2 times the upper reference limit)
- Blood Urea Nitrogen
- Creatinine
- Uric Acid
- Total Protein
- Albumin
- Total Bilirubin
- Sodium
- Potassium
- Glucose (HbA_{1c} if glucose >126 mg/dl)
- Calcium
- HbA_{1c}
- TSH (at Visit 1 and as described on the LDL action plan)

2. URINALYSIS

Determinations by dipstick* (at the site)
- Leukocytes
- Nitrite
- Urobilinogen
- Protein
- pH
- Blood
- Specific Gravity

Ketone
Bilirubin
Glucose

* If leukocytes, nitrite, protein, or blood are positive, a urine sample must be sent to the central laboratory for microscopic evaluation.

3. SAFETY LABORATORY DETERMINATIONS

SGOT (AST)
SGPT (ALT)

New Appendices D, E, F are now attached via hard-copy and are not paginated electronically within this document.

APPENDIX D
Administrative Procedures for the Reporting of Adverse Events

APPENDIX D

Administrative Procedures for the Reporting of Adverse Events

June 13, 2003 – Amendment 4

INTRODUCTION

The administrative procedures for reporting adverse events described in this appendix are to be followed during the conduct of this protocol.

If you have any questions concerning adverse event reporting, please contact the ***Pfizer Inc and its affiliates*** colleague or representative who is monitoring your site or a ***Pfizer Inc and its affiliates*** Clinical/Medical Colleague whose name, address, and telephone number appears on the cover sheet to this protocol.

TABLE OF CONTENTS

Page

1. STANDARD SAFETY SECTION FOR INCLUSION IN PFIZER-SPONSORED CLINICAL TRIAL PROTOCOLS D4

1.1. Adverse Events D4
1.2. Serious Adverse Events D5
1.3. Abnormal Laboratory Test Results D7
1.4. Abnormal Physical Examination Findings D8
1.5. Discontinuations D9

2. DEFINITIONS D-9
2.1. Attributes of Adverse Events D-10
2.1.1. Treatment-Emergent Signs and Symptoms (TESS) D-10
2.1.2. Serious Adverse Events D-10
2.1.3. Intensity D-10
2.1.4. Relationship to Study Drug—Physician's Assessment D-11
2.1.5. Clinical Outcome D-12

3. CAPTURING ADVERSE EVENTS D-12
3.1. Pre-Existing Condition D-12
3.2. Lack of Efficacy D-12
3.3. <letter/sentence/paragraph deleted> Hospitalization or Surgery/Procedure . D-13
3.4. Death D-13
3.5. General Physical Examination Findings D-13

4. REPORTING TO THE SPONSOR. D-13
4.1. Immediately Reportable Adverse Events D-13
4.2. Other Adverse Events D-14
4.3. Follow-up Period D-14

1. Standard safety section for inclusion in pfizer-sponsored clinical trial protocols

1.1. Adverse Events

All observed or volunteered adverse events, regardless of treatment group or suspected causal relationship to study drug, will be recorded on the adverse event page(s) of the case report form.

Events involving adverse drug reactions, illnesses with onset during the study, or exacerbations of pre-existing illnesses should be recorded. Exacerbation of pre-existing illness, including the disease under study, is defined as a manifestation (sign or symptom) of the illness that indicates a significant increase in the severity of the illness as compared to the severity noted at the start of the trial. It may include worsening or increase in severity of signs or symptoms of the illness, increase in frequency of signs and symptoms of an intermittent illness, or the appearance of a new manifestation/complication. Exacerbation of a pre-existing illness should be considered when a patient/subject requires new or additional concomitant drug or non-drug therapy for the treatment of that illness during the trial. Lack of or insufficient clinical response, benefit, efficacy, therapeutic effect, or pharmacologic action, should not be recorded as an adverse event. The investigator must make the distinction between exacerbation of pre-existing illness and lack of therapeutic efficacy.

In addition, clinically significant changes in physical examination findings and abnormal objective test findings (eg, laboratory, x-ray, ECG) should also be recorded as adverse events. The criteria for determining whether an abnormal objective test finding should be reported as an adverse event are as follows:

1) ***Test result is associated with accompanying symptoms; and/or***
2) ***Test result requires additional diagnostic testing or medical/surgical intervention; and/or***
3) ***Test result leads to a change in study dosing or discontinuation from the study, significant additional concomitant drug treatment or other therapy; and/or***
4) ***Test result leads to any of the outcomes included in the definition of a serious adverse event; and/or***
5) ***Test result is considered to be an adverse event by the investigator or sponsor.***

Merely repeating an abnormal test, in the absence of any of the above conditions, does not meet condition #2 above for reporting as an adverse event.

Any abnormal test result that is determined to be an error does not require reporting as an adverse event, even if it did meet one of the above conditions except for condition #4.

For all adverse events, the investigator must pursue and obtain information adequate both to determine the outcome of the adverse event and to assess whether it meets the

criteria for classification as a serious adverse event requiring immediate notification to Pfizer or its designated representative (see Section 1.2). For all adverse events, sufficient information should be obtained by the investigator to determine the causality of the adverse event (ie, study drug or other illness). The investigator is required to assess causality and indicate that assessment on the CRF. Follow-up of the adverse event, after the date of therapy discontinuation, is required if the adverse event or its sequelae persist. Follow-up is required until the event or its sequelae resolve or stabilize at a level acceptable to the investigator and the Pfizer clinical monitor or his/her designated representative.

1.2. Serious Adverse Events

All serious adverse events (as defined below), regardless of treatment group or suspected relationship to study drug, must be reported immediately by telephone to the named individuals identified within the protocol text.

A serious adverse event is any adverse drug experience occurring at any dose that:

1) ***results in death;***
2) ***is life-threatening;***
3) ***results in inpatient hospitalization or prolongation of existing hospitalization;***
4) ***results in a persistent or significant disability/incapacity; or***
5) ***results in congenital anomaly/birth defect.***

Important medical events that may not result in death, be life-threatening, or require hospitalization may be considered serious adverse drug experiences when, based upon appropriate medical judgment, they may jeopardize the patient/subject and may require medical or surgical intervention to prevent one of the outcomes listed in this definition. Examples of such medical events include allergic bronchospasm requiring intensive treatment in an emergency room or at home, blood dyscrasias or convulsions that do not result in inpatient hospitalization, or the development of drug dependency or drug abuse.

Regardless of the above criteria, any additional adverse experiences which Pfizer personnel or an investigator considers serious should be immediately reported to Pfizer and included in the Corporate adverse events database system.

A life-threatening adverse event is any adverse drug experience that places the patient/subject at immediate risk of death from the reaction as it occurred; ie, it does not include a reaction that, had it occurred in a more severe form, might have caused death.

Initial hospitalization is defined as any inpatient admission (even if less than 24 hours). For chronic or long-term inpatients, inpatient admission also includes transfer within the hospital to an acute/intensive care inpatient unit (eg, from the psychiatric wing to a medical floor, from a medical floor to the coronary care unit, from the neurological floor to the tuberculosis unit).

1) *Inpatient admission in the absence of a precipitating, treatment-emergent, clinical adverse event may meet criteria for "seriousness" but is not an adverse experience and thus is not subject to immediate reporting to Pfizer. For example:*

   i. *Admission for treatment of a pre-existing condition not associated with the development of a new adverse event or with a worsening of the pre-existing condition (eg, for work-up of persistent pretreatment lab abnormality)*
   ii. *Social admission (eg, subject has no place to sleep)*
   iii. *Administrative admission (eg, for yearly physical exam)*
   iv. *Protocol-specified admission during a clinical trial (eg, for a procedure required by the study protocol)*
   v. *Optional admission not associated with a precipitating clinical adverse event (eg, for elective cosmetic surgery)*

   However, if a hospitalization for an unknown event occurs, it should be considered as a serious adverse event.

2) *Inpatient admission does not include the following:*

   i. *Emergency Room/Accident and Emergency/Casualty Department visits*
   ii. *Outpatient/same-day/ambulatory procedures*
   iii. *Observation/short-stay units*
   iv. *Rehabilitation facilities*
   v. *Hospice facilities*
   vi. *Respite care (eg, caregiver relief)*
   vii. *Skilled nursing facilities*
   viii. *Nursing homes*
   ix. *Custodial care facilities*
   x. *Clinical research/Phase I units*

Prolongation of hospitalization is defined as any extension of an inpatient hospitalization beyond the stay anticipated/required in relation to the original reason for the initial admission, as determined by the investigator or treating physician. For protocol-specified hospitalizations in clinical trials, prolongation is defined as any extension beyond the length of stay described in the protocol. Prolongation in the absence of a precipitating, treatment-emergent, clinical adverse event (ie, not associated with the development of a new adverse event or worsening of a pre-existing condition) may meet criteria for "seriousness" but is not an adverse experience and thus is not subject to immediate reporting to Pfizer.

Preplanned treatments or surgical procedures should be noted in the baseline documentation for the entire protocol and/or for the individual patient/subject.

Disability is a substantial disruption of a person's ability to conduct normal life functions.

Any serious adverse event or death must be reported immediately independent of the circumstances or suspected cause if it occurs or comes to the attention of the

*investigator at any time during the study through the last follow-up visit required by the protocol or 30 days after the last administration of study drug, whichever comes later. Any serious adverse event occurring at any other time after completion of the study must be promptly reported if a causal relationship to study drug is suspected. The only exception to these reporting requirements are serious adverse events that occur during a prerandomization/
washout run-in period, during which either placebo alone is administered, or no active study drug or no protocol-specified background drug is administered.*

For all serious adverse events, the investigator is obligated to pursue and provide information as requested by the Pfizer clinical monitor or designated representative in addition to that on the case report form. In general, this will include a description of the adverse event in sufficient detail to allow for a complete medical assessment of the case and independent determination of possible causality. Information on other possible causes of the event, including concomitant medications and illnesses must be provided. The investigator's assessment of causality must also be provided. If causality is unknown and the investigator does not know whether or not study drug caused the event, then it should be attributed to study drug. If the investigator's causality assessment is "unknown but not related to study drug," this should be clearly documented on study records. In the case of a subject death, a summary of available autopsy findings must be submitted as soon as possible to Pfizer or its designated representative. The investigator should ensure that information reported immediately by telephone or other means and information entered in the case report form are accurate and consistent.

1.3. Abnormal Laboratory Test Results

The results of all laboratory tests required by the protocol will be recorded in the subject's case report form. All clinically important abnormal laboratory tests occurring during the study will be repeated at appropriate intervals until they return either to baseline or to a level deemed acceptable by the investigator and the Pfizer clinical monitor (or his/her designated representative), or until a diagnosis that explains them is made. The criteria for determining whether an abnormal objective test finding should be reported as an adverse event are as follows:

1) *Test result is associated with accompanying symptoms, and/or*
2) *Test result requires additional diagnostic testing or medical/surgical intervention, and/or*
3) *Test result leads to a change in study dosing or discontinuation from the study, significant additional concomitant drug treatment or other therapy, and/or*
4) *Test result leads to any of the outcomes included in the definition of a serious adverse event, and/or*
5) *Test result is considered to be an adverse event by the investigator or sponsor.*

Merely repeating an abnormal test, in the absence of any of the above conditions, does not meet condition #2 above for reporting as an adverse event.

Any abnormal test result that is determined to be an error does not require reporting as an adverse event, even if it did meet one of the above conditions except for condition #4.

1.4. Abnormal Physical Examination Findings
Clinically significant changes, in the judgment of the investigator, in physical examination findings (abnormalities) will be recorded as adverse events.

1.5. Discontinuations
The reason for a subject discontinuing from the study will be recorded in the case report form. A discontinuation occurs when an enrolled subject ceases participation in the study, regardless of the circumstances, prior to completion of the protocol. The investigator must determine the primary reason for discontinuation. Withdrawal due to adverse event should be distinguished from withdrawal due to insufficient response according to the definition of adverse event noted earlier. A discontinuation must be reported immediately to the Pfizer clinical monitor or his/her designated representative if it is due to a serious adverse event. The final evaluation required by the protocol will be performed at the time of study discontinuation. The investigator will record the reason for study discontinuation, provide or arrange for appropriate follow-up (if required) for such subjects, and document the course of the subject's condition.

June 13, 2003 – Amendment 4
2. DEFINITIONS
Pre-Existing Condition

A pre-existing condition is one that is present at the start of study treatment.

Baseline

Defined by the ***protocol as applicable to the study.***

<Letter/Sentence/Paragraph Deleted>

Related Adverse Event

An AE where there is a reasonable possibility that the experience may have been caused by drug (Unknown is also considered related).

<Letter/Sentence/Paragraph Deleted>

<Letter/Sentence/Paragraph Deleted>

Unexpected Adverse Event

Any adverse event, the specificity or severity of which is not consistent with the current Investigator's Brochure. For example, under this definition, hepatic necrosis would be unexpected (by virtue of greater severity) if the Investigator's Brochure only referred to elevated hepatic enzymes or hepatitis.

Similarly, cerebral thromboembolism and cerebral vasculitis would be unexpected (by virtue of greater specificity) if the Investigator's Brochure only listed cerebral vascular accidents. "Unexpected," as used in this definition, refers to an AE that has not been previously observed (eg, included in the Investigator's Brochure) rather than from the perspective of such experience not being anticipated from the pharmacological properties of the pharmaceutical product.

<Letter/Sentence/Paragraph Deleted>

Treatment-Emergent Signs and Symptoms (TESS)

Any AE that was not evident during baseline as defined by the study protocol, or that increases in intensity or frequency, or changes in character during treatment.

Treatment-Emergent Signs and Symptoms (TESS)

Any AE that was not evident during baseline as defined by the study protocol, or that increases in intensity or frequency, or changes in character during treatment.

Posttreatment Adverse Event

Any AE that occurs after study treatment is discontinued. Posttreatment follow-up and posttreatment adverse events of interest to the study will be defined per protocol.

Lack of Efficacy

A worsening of the disease being studied or lack of desired effect of the study drug (not reported as an AE if defined as an efficacy parameter in the protocol).

2.1. Attributes of Adverse Events

2.1.1. Treatment-Emergent Signs and Symptoms (TESS)

Any condition/diagnosis that meets the definition of a TESS event is captured as such on the AE CRF.

June 13, 2003 – Amendment 4
2.1.2. Serious Adverse Events

All SAEs, as defined in Section *1.2*, are immediately reportable to ***Pfizer Inc. and its affiliates. <Letter/Sentence/Paragraph Deleted>***

If there is an exception to the SAE definition, it is described in the protocol.

2.1.3. Intensity

The following criteria are used to assess the intensity of each AE:

- *Mild*: The subject/patient is aware of the sign or symptom, but finds it easily tolerated.
- *Moderate*: The subject/patient has discomfort enough to cause interference with or change in usual activities.
- *Severe:* The subject/patient is incapacitated and unable to work or participate in many or all usual activities.

Note: For oncology studies, in lieu of these criteria, standardized coding criteria may be used as defined in the protocol.

June 13, 2003 – Amendment 4

2.1.4. Relationship to Study Drug—Physician's Assessment

There are 6 categories for the physician's assessment of the causal relationship between study drug and an AE:

Physician's Assessment	*Causal Relationship Reported to Regulatory Agencies*
Definitely	*Yes*
Probably	*Yes*
Possibly	*Yes*
Unlikely	*No*
Definitely Not	*No*
Unknown[a]	*Yes (ie, interpreted as "possibly" related)*

[a] ***Insufficient information or indeterminable, too***

The following criteria are to be used to assess the causal relationship between an AE and study drug:

Definite:

> ***Follows a reasonable temporal sequence from administration of the drug or from drug levels established in body fluids or tissues; AE improves or disappears after stopping or reducing the dosage (dechallenge); AE reappears with rechallenge.***

or

> ***Is known to be associated with the drug or class of compounds and cannot be explained by other therapy or subject/patient's physical condition.***

Probable:

> ***Follows a reasonable temporal sequence from drug administration; AE improves or disappears after stopping or reducing the dosage (dechallenge) and rechallenge was not attempted; AE cannot be explained by other therapy or the subject/patient's physical condition.***

Possible (Reasonable Possibility):

Plausible temporal sequence; it is reasonable to suspect drug causation after considering other therapy or the subject/patient's physical condition.

Unlikely:

Does not meet above criteria and current knowledge indicates that a relationship is extremely unlikely.

Definitely Not:

Does not meet above criteria; AE is known to be associated with other therapy or the subject's/patient's physical condition.

Unknown:

Not possible to assign to any other category.

<u>June 13, 2003 – Amendment 4</u>
2.1.5. Clinical Outcome
The following categories are used to assess the clinical outcome of each AE:

- ***Unknown:***

  The outcome of the event is not known at the time of reporting. Attempt to obtain information for follow-up report.

- ***Patient Recovered***
- ***Patient has Not Yet Recovered:***
  The patient still has symptoms. This can be identified as "Recovering", "Recovered with Sequelae", or "Change in Intensity".
- ***Patient Died Due to Adverse Events or Patient Died due to Other Causes:***

  If the patient died, whether or not the death is attributed to the adverse event. The date and probable cause(s) of death should be reported.

3. CAPTURING ADVERSE EVENTS

3.1. Pre-Existing Condition

A pre-existing condition should be reported as an AE if the frequency, intensity, or the character of the condition worsens during study treatment.

3.2. Lack of Efficacy

Signs or symptoms defined in the protocol as lack of efficacy or collected as efficacy parameters should not be reported as AEs.

June 13, 2003 – Amendment 4

3.3. *<Letter/Sentence/Paragraph Deleted>* Clinical Laboratory Adverse Event

A clinical laboratory abnormality should be reported as an AE only if the conditions are met as defined in Section 2.

Any AE that results in hospitalization (ie, subject/patient admitted—not just an emergency room visit) should be reported as an SAE unless specifically instructed otherwise in the protocol. Any condition/diagnosis responsible for surgery/procedure should be reported as an AE if it meets the criteria for an AE. The surgery/procedure itself will be reported as a Clinical Outcome of the underlying event.

Events that prolong any hospitalization are reported as SAEs.

3.4. Death

The cause of death should be reported as an AE. Death should not be reported as an AE, but as a Clinical Outcome. The only exception is "Sudden Death" when the cause of death is unknown, which is reported as an AE with death as the Clinical Outcome.

June 13, 2003 – Amendment 4

3.5. General Physical Examination Findings

At screening, any clinically significant finding should be recorded on the General Medical History CRF. After the signing of the informed consent document and being formally accepted into the study, any new clinically significant finding that meets the definition of an AE must be documented as such. ***Illnesses or medical events discovered after signing Informed Consent during screening phase but prior to meeting all inclusion/ exclusion criteria should be considered a pre-existing condition, and therefore, not recorded as an AE.***

4. REPORTING TO THE SPONSOR

All AEs that occur at any time during the study including the posttreatment period as defined in the protocol, are to be reported in the subject's/patient's CRFs.

__June 13, 2003 – Amendment 4__

4.1. Immediately Reportable Adverse Events

If an AE meets the definition of Serious (see Section *1*), it is *immediately reportable*. The investigator should contact ***Pfizer Inc and its affiliates*** by telephone or fax ***and report event*** to the Clinical/Medical Colleague ***immediately***. In addition, the investigator must complete an AE CRF. If any SAE occurs, the investigator can withdraw the subject/patient from the study at the investigator's discretion while taking the appropriate follow-up action.

The Pfizer Inc and its affiliates Worldwide Adverse Event Monitoring System ***(AEM)*** is the database ***Pfizer Inc and its affiliates uses*** to collect SAE information from all studies. For each immediately reportable ***SAE, an AEM*** form must be filled out by the ***Clinical/Medical Colleague*** with the assistance of the investigator/site personnel, describing what is known about the event and its management.
<Letter/Sentence/Paragraph Deleted>

The ***AEM*** form is IN ADDITION TO, not instead of, the standard AE CRF. Any discrepancies between the completed standard AE CRF and the AEM form should be resolved at the time the event is being evaluated and must be resolved prior to submission of the AE CRF to ***Pfizer Inc and its affiliates***.

<Letter/Sentence/Paragraph Deleted>

__June 13, 2003 – Amendment 4__

4.2. Other Adverse Events

AEs that are not immediately reportable according to the definitions in this appendix will be recorded on the standard AE CRF. ***<Letter/Sentence/Paragraph Deleted>***

4.3. Follow-up Period

For SAEs, the subject/patient must remain under observation until the SAE has subsided or stabilized and all serious pathological values and findings have returned to normal or stabilized.

Follow-up information will not be collected for "not yet recovered" or continuing nonserious AEs unless time frames are specifically written in the protocol.

APPENDIX E

Other Administrative and Regulatory Procedures

APPENDIX E

Other Administrative and Regulatory Procedures

INTRODUCTION

This appendix provides information necessary to administer this study in compliance with global GCPs, government regulations, and the policy and procedures of Parke-Davis Pharmaceutical Research, Warner-Lambert Company.

If you have any questions concerning the conduct of the study, contact 1 of the Parke-Davis Clinical/Medical Colleagues whose name, address, and telephone number appears on the signature sheet to this protocol.

Your signature on this cover page of the protocol, any subsequent amendments and addenda, and the Clinical Study Agreement confirms that you:

- Have been given appropriate information on the study drug;
- Have read and understood the protocol and appendices;
- Agree to conduct the study in accordance with the provisions of the protocol and applicable regulations;
- Acknowledge Parke-Davis' ownership of the data and results obtained from the conduct of this protocol; and
- Agree to maintain the confidentiality of certain information (see Section 1.8.1).

TABLE OF CONTENTS

Page

1. ADMINISTRATIVE PROCEDURES E-1
1.1. Ethics and Informed Consent E-1
1.1.1. Declaration of Helsinki E-1
1.1.2. Institutional Review Board (IRB) or Ethics Committee (EC) Review and Approval of the Study E-1
1.1.3. Subject/Patient Informed Consent E-2
1.2. Clinical Evaluations Not Specified in the Protocol E-4
1.3. Monitoring the Study E-4
1.3.1. Prestudy Visit E-4
1.3.2. Investigator's Meeting E-5
1.3.3. Study Start-Up Visit E-5
1.3.4. Monitor Visits and Phone Contacts E-5
1.3.4.1. Source Data Verification E-6
1.3.5. End-of-Study Visit E-6
1.3.6. Communicating Deficiencies E-7
1.4. Medication Dispensing E-7
1.5. Randomization Code E-7
1.6. Emergency Information E-8
1.6.1. Subject/Patient Emergency Information Card E-8
1.6.2. Emergency Code Breaks (Blinded Studies Only) E-8
1.7. Confidentiality of Subject/Patient Information E-8
1.8. Study Summarization and Publication of Study Results E-8
1.8.1. Intellectual Property Rights E-9

2. DOCUMENTATION PROCEDURES E-10
2.1. Information Required by Parke-Davis for Regulatory Review E-10
2.1.1. Prior to Study Initiation E-10
2.1.1.1. Studies Conducted Under a US Investigational New Drug (IND) Application E-10
2.1.1.2. Studies Not Conducted Under a US Investigational New Drug (IND) Application E-10
2.1.2. During the Study E-11
2.1.3. End-of-Study E-11
2.1.4. Retention of Study Records E-11
2.2. Guidelines for Recording Data in the Case Report Forms E-12
2.3. Review of Case Report Forms E-13
2.4. Investigator's Responsibility for Clinical Drug Supply Accountability E-13
2.4.1. Receipt of Clinical Drug Supplies E-13

TABLE OF CONTENTS

Page

2.4.2. Storage E-13
2.4.3. Dispensing of Clinical Drug Supplies E-14
2.4.4. Return of Clinical Drug Supplies E-14

3. PROTOCOL AMENDMENTS AND ADDENDA E-14
3.1. Urgent Protocol Amendment E-15
3.2. Other Amendments/Addenda E-15
3.3. Study Termination E-16

1. ADMINISTRATIVE PROCEDURES

1.1. Ethics and Informed Consent

1.1.1. Declaration of Helsinki

This study will be conducted in accordance with the Declaration of Helsinki (see Appendix F if US study or Appendix F.1 if International study).

1.1.2. Institutional Review Board (IRB) or Ethics Committee (EC) Review and Approval of the Study

An IRB/EC, that is organized and operates according to GCP and applicable laws and regulations, should safeguard the rights, safety, and well-being of all trial subjects/patients. No subject/patient should be admitted to a trial before the IRB/EC issues its written approval/favorable opinion of the trial.

The investigator is responsible for:

- Promptly reporting to the IRB/EC all changes in the research activity, all unlabeled adverse events, and all unanticipated problems involving risks to human subject/patients or others;
- Not making any changes in the research without IRB/EC approval, except when absolutely necessary to eliminate apparent immediate hazards to human subject/patients;
- Submitting a progress report describing the status of the clinical investigation to the IRB/EC at appropriate intervals not exceeding 1 year; and
- Submitting a final report when required by the IRB/EC within 3 months following completion, termination, or discontinuation of the study. Copies of these reports will also be provided to Parke-Davis.

In general, all communications with the IRB/EC regarding the study of a Parke-Davis drug will be handled by the principal investigator (or coordinating investigator, if applicable) of the study. Parke-Davis personnel may directly contact the IRB/EC if necessary, but must not attempt to influence the IRB/EC in any way. A copy of all communications from the IRB/EC to the investigator regarding its review of and initial approval of the study and its reapprovals at intervals must be provided to the Parke-Davis Site Monitor by the investigator.

1.1.3. Subject/Patient Informed Consent

The investigator must fully explain the purpose of the study to the subject/patient or his/her guardian prior to entering the subject/patient into the study. The investigator is responsible for obtaining written informed consent from each subject/patient. For subjects/patients under the legal age of consent or unable to provide written consent, written informed consent must be obtained from his/her legal guardian, or legal representative. Also, the assent of a child to participate in the study must be obtained when appropriate, eg, in consideration of the child's age and maturity.

Parke-Davis requires that informed consent be obtained orally and on a written form prepared by the investigator and approved by the IRB/EC. Although a sample Informed Consent Document (ICD) may be provided to international investigators or a template to US investigators, the investigator is ultimately responsible for the content of the document.

The person signing the consent form will receive a copy of the signed form. The signed consent form will be filed at the site with the investigator's copies of the Case Report Forms (CRFs) for each subject/patient.

The consent form is as follows:

- A written document that includes the informed consent requirements listed below. This form may be read to the subject/patient or the legally authorized representative, who will have adequate opportunity to read it, ask questions, and receive requested information before signing.

Except as provided in δ 50.23, Exception From General Requirements (eg, emergency drug use situations), of the US Code of Federal Regulations, no investigator may involve a human being as a subject/patient in research covered by these regulations unless the investigator has obtained the legally effective informed consent of the subject/patient or his/her legally authorized representative. An investigator shall seek such consent only under circumstances that provide the prospective subject/patient or the representative sufficient opportunity to consider whether or not to participate and that minimize the possibility of coercion or undue influence. The information that is given to the subject/patient or the representative shall be in language understandable to the subject/patient or representative. No informed consent may include any exculpatory language through which the subject/patient or representative is made to waive any of the subject's/patient's legal rights, or releases, or appears to release the investigator, the sponsor, the institution, or its agents from liability for negligence.

All studies conducted worldwide must have an ICD that includes the following elements of informed consent listed below:

- That the trial involves research.
- The purpose of the trial.
- The trial treatment(s) and the probability for random assignment to each treatment.
- The trial procedures to be followed, including all invasive procedures.
- The subject's/patient's responsibilities.
- Those aspects of the trial that are experimental.
- The reasonably foreseeable risks or inconveniences to subject/patient and, when applicable, to an embryo, fetus, or nursing infant.
- The reasonably expected benefits. When there is no intended clinical benefit to the subject/patient, the subject/patient should be made aware of this.
- The alternative procedure(s) or course(s) of treatment that may be available to the subject/patient, and their important potential benefits and risks.
- The compensation and/or treatment available to the subject/patient in the event of trial-related injury.
- The anticipated prorated payment, if any, to the subject/patient for participating in the trial.
- The anticipated expenses, if any, to the subject/patient for participating in the trial.
- That the subject's/patient's participation in the trial is voluntary and that the subject/patient may refuse to participate or withdraw from the trial, at any time, without penalty or loss of benefits to which the subject/patient is otherwise entitled.
- That the monitor(s), the auditor(s), the IRB/EC, and the regulatory authority(ies) will be granted direct access to the subject's/patient's original medical records for verification of clinical trial procedures and/or data, without violating the confidentiality of the subject/patient, to the extent permitted by the applicable laws and regulations and that, by signing an informed consent form, the subject/patient or the subject's/patient's legally acceptable representative is authorizing such access.
- That records identifying the subject/patient will be kept confidential and, to the extent permitted by applicable laws and/or regulations, will not be made publicly available. If the results of the trial are published, the subject's/patient's identity will remain confidential.
- That the subject/patient or the subject's/patient's legally acceptable representative will be informed in a timely manner if information becomes available that may be relevant to the subject's/patient's willingness to continue participation in the trial.

- The person(s) to contact for further information regarding the trial and the rights of trial subjects/patients, and whom to contact in the event of trial-related injury.
- The foreseeable circumstances and/or reasons under which the subject's/patient's participation in the trial may be terminated.
- The expected duration of the subject's/patient's participation in the trial.
- The approximate number of subjects/patients involved in the trial.

These informed consent requirements are not intended to preempt any applicable Federal, State, local laws which require additional information to be disclosed for informed consent to be legally effective.

Nothing in these regulations is intended to limit the authority of a physician to provide emergency medical care to the extent the physician is permitted to do so under applicable Federal, State, or local law.

1.2. Clinical Evaluations Not Specified in the Protocol

Procedures not specified in the protocol can be conducted only if required for the successful management of a subject/patient or they will not affect the conduct or results of the study and each procedure should be approved by the Parke-Davis Clinical/Medical Colleague.

1.3. Monitoring the Study

Frequent contact between the principal investigator and Parke-Davis will be maintained by the Clinical/Medical Colleague and/or the Site Monitor, or comparable persons from a designated Contract Research Organization appointed by Parke-Davis, to assure that this study is conducted according to the protocol and that all forms are accurate and complete prior to being forwarded to Parke-Davis.

1.3.1. Prestudy Visit

Representatives from Parke-Davis, usually those who will monitor the site, will visit the investigator, site staff, and study facility prior to initiation of the clinical study to:

- Review the key elements of the available information on the investigational drug and the protocol;
- Determine the site's ability to conduct the study based on, for example, experience, adequacy of physical facilities;
- Availability of an adequate, suitable patient population; and

- IRB/EC involvement and accessibility for site monitor visits including source document verification against medical records.

If the prospective investigator has conducted a study for the Company within the last 12 months with the same investigational drug, a telephone interview may be used to replace an on-site visit at the discretion of the Study Manager. For clinical pharmacology studies, a telephone interview may also be used, if the prospective investigator has conducted the same study type (eg, special populations, renal, hepatic, safety tolerance, PK, interaction studies).

1.3.2. Investigator's Meeting

Principal investigators must not enroll patients into a study unless they have received training on all aspects of a clinical study. For multicenter studies, this training is typically provided at an Investigator's Meeting. A prestudy Investigator's meeting is a formal meeting between the sponsor and the investigators and their staff to review all aspects of a clinical study prior to study initiation. The main purposes of this meeting include:

- Reviewing the study protocol and procedures;
- Resolving any questions regarding the purpose(s) and conduct of the study;
- Instructing all participants in the administrative and regulatory (ie, GCP) procedures to be followed.

1.3.3. Study Start-Up Visit

This visit, which is conducted by the Site Monitor, typically occurs after the Investigator's Meeting and after written study materials and clinical drug supplies are at the site, and before subject/patient enrollment. The purpose of this visit is to ensure that both Parke-Davis and the investigator site have fulfilled all necessary obligations before treating subjects/patients in a clinical trial. During the visit, the Site Monitor reviews with the investigator and appropriate site staff the key elements of study documents and supplies and their study obligations and roles.

For some studies, the Drug or Study Manager may waive the Study Start-Up Visit if the goals have been accomplished via a Prestudy Visit and/or Investigator's Meeting or the site has participated in other similar studies for Parke-Davis within the past 12 months.

1.3.4. Monitor Visits and Phone Contacts

Routine monitoring visits, at appropriate intervals detailed in the monitoring guidelines, will be scheduled based on the design and complexity of the protocol and the timelines for obtaining study results to:

- Verify that the rights and well-being of human subjects/patients are protected (eg, signed informed consent document, IRB/EC approvals, etc);
- Ensure study conduct in accordance with the protocol (and any amendments/addenda), local regulatory requirements, Parke-Davis procedures, and GCPs;
- Verify the accuracy and timely completion of the recorded data (eg, CRFs) with respect to source documents (see Section 1.3.4.1 below);
- Resolve data queries;
- Collect data and biological samples in a timely manner;
- Maintain an accurate record of study progress;
- Account accurately for clinical drug supplies
- Ensure continued acceptability of facilities for study conduct;
- Ensure appropriate maintenance of required documents;
- Prepare the site for audits.

In addition, both Parke-Davis and the site are responsible for documenting substantive telephone conversations (eg, those about inclusion/exclusion criteria in the protocol, medication dosing procedures, adverse events, and emergency code breaks).

1.3.4.1. Source Data Verification

The purpose of Source Data Verification (SDV; also known as Source Document Verification) is to ensure as much as possible the accuracy, quality, and reality of the data recorded on CRFs via a comparison to source documents. Source documents are the original documents or records where raw/source data concerning a subject/patient have been first recorded, eg, medical charts, hospital records, clinical laboratory reports, X-rays, automated instrument tracings, signed and dated informed consent documents.

1.3.5. End-of-Study Visit

The final on-site monitoring visit is conducted when:

- The investigator completes the study or requests to discontinue participation in the study; or
- Parke-Davis decides to discontinue the study for all investigators in a trial (eg, due to adverse events or study enrollment being reached) or for an individual investigator (eg, due to poor patient enrollment).

Purposes of the End-of-Study Visit include:

- Reviewing and ensuring completeness of required documentation and documentation retention policies;
- Collecting completed CRFs;
- Ensuring that all adverse events have been identified, documented and reported to the Company;
- Ensuring final returning/disposition of any remaining clinical drug supplies;
- Ensuring that the investigator has notified the IRB/EC of study completion or discontinuation;
- Reviewing processes that occur after the End-of-Study Visit; and
- Finalizing payment issues.

1.3.6. Communicating Deficiencies

The Parke-Davis Site Monitor will inform the site of any deficiencies related to the conduct of the study noted during monitoring visits or audits conducted by Parke-Davis QA colleagues.

1.4. Medication Dispensing

Each medication container will be supplied with a tear-off label that is to be attached to the appropriate form (ie, Medication Dispensing Record [MDR]) when the medication is dispensed. A detailed set of dispensing instructions and Investigational Drug Release Authorization (IDRA), which is the process by which Parke-Davis sends study drugs to investigator sites, will be included with the drug shipment. MDRs should be kept current during the study and be available for inspections. Copies are to be forwarded to Parke-Davis.

During and at the end of the study, handle all containers, whether empty or not, as instructed by the protocol and Site Monitor.

1.5. Randomization Code

The Clinical Pharmaceutical Operations (CPO) Department of Parke-Davis (or biometrics department locally) generates the randomization code, and then CPO or other designated facility will provide medication assembled for each subject/patient based on a randomization code.

1.6. Emergency Information

1.6.1. Subject/Patient Emergency Information Card

At least 1 of the investigators at each site will be available to the subject/patient at all times during their participation in the study. The subject/patient will be provided an emergency information card that contains all necessary information to contact the investigator in the case of an emergency, along with a description of the study medications the subject/patient may have received and possible emergency precautions to be taken (if any). The subject/patient will be instructed to carry this card with him/her at all times during the study.

The requirement for the subject/patient Emergency Information Card will be waived if patients are to be hospitalized throughout the study, including the protocol-defined follow-up period.

1.6.2. Emergency Code Breaks (Blinded Studies Only)

The investigator will be provided with a mechanism for emergency determination of a subject's/patient's treatment regimen in the event that contact with the Parke-Davis Clinical/Medical colleague is not possible and this is required for proper treatment of the subject/patient. Depending on the study, this is either a medication code card for one or more patients or a personal identification number (PIN) for telephone use. The study medication given to an individual subject/patient can be ascertained without breaking the code for other subjects/patients in the study. The date and reason for breaking the code for must be documented in the Investigator Study File. Code cards will be kept on file by the principal investigator and, if applicable, at a designated secondary site. This material will be returned to the sponsor at the end of the study.

1.7. Confidentiality of Subject/Patient Information

All subjects/patient will be assigned a study subject/patient number. Subsequently, subjects/patients will be identified in the CRFs only by their initials and that number. Any information published as a result of the study will be such that it will not permit identification of any subject/patient. The information from this study will be available within Parke-Davis and may be shared with the regulatory authorities. It may also be the subject of an audit by a regulatory agency (eg, FDA) within the local government. The subject's/patient's identity will remain protected except as required for legal or regulatory inquiries.

1.8. Study Summarization and Publication of Study Results

Data analysis, statistical reporting, and research report preparation will be the responsibility of Parke-Davis or a duly authorized designee.

All information and data regarding the study drugs obtained in connection with the conduct of this study are considered confidential. Accordingly, Parke-Davis retains the right to review manuscripts, abstracts, and presentation material related to this protocol and its amendments/addenda prior to presentation or submission to a journal. Such articles will not be submitted for publication without the prior approval of Parke-Davis. This review will not restrict publication of facts or opinions formulated by the investigator.

The consent of Parke-Davis may be withheld, at least temporarily, either to protect the patentability of its compounds, to prevent real or perceived bias in ongoing or planned controlled clinical trials, to prevent an adverse impact on the completion of clinical programs necessary to submit a New Drug Application (NDA) for marketing to the FDA or a Marketing Authorization Application (MAA) to another international regulatory authority, or for any other legitimate business reason. This ensures consistency with corporate goals and protects legitimate proprietary interests.

Information regarding the operations and procedures of Parke-Davis obtained as a result of or in association with the conduct of this study must be kept confidential. At no time may information on the operations of Parke-Davis be disclosed directly or indirectly to representatives or agents of other pharmaceutical companies or commercial entities.

Parke-Davis is prepared to review expeditiously material proposed for presentation and/or publication. Please submit manuscripts, abstracts, presentations, or other publication drafts to the Parke-Davis Clinical/Medical Colleague. In the case of Clinical Research Department studies, the study manager would then forward these directly to:

Director, Clinical Communications Department
Parke-Davis Pharmaceutical Research
Division of Warner-Lambert Company
2800 Plymouth Road
Ann Arbor, MI 48105

Questions regarding assistance in preparation of materials should be directed to the Parke-Davis Clinical/Medical Colleague.

1.8.1. Intellectual Property Rights

By signing a Clinical Study Agreement (CSA), the investigator agrees to keep in confidence and use only for completion of the study:

- Information provided to him/her by or on the behalf of Parke-Davis; and
- Data, inventions, and discoveries, generated as a result of the study.

The investigator also agrees to return all copies of such information to Parke-Davis at the sponsor's request and that data, inventions, and discoveries generated during the course of

this study shall be the property of Parke-Davis and will sign any documents, if requested, to transfer such ownership. This obligation will not apply to any information or data that later becomes public knowledge.

2. DOCUMENTATION PROCEDURES

2.1. Information Required by Parke-Davis for Regulatory Review

This study will not start in any country until the requirements of Parke-Davis and for regulatory approval have been satisfied for that country. The following documents and information must be provided by the principal investigator to Parke-Davis:

2.1.1. Prior to Study Initiation

2.1.1.1. Studies Conducted Under a US Investigational New Drug (IND) Application

- Signed protocol (and amendments/addenda, if applicable);
- A completed, signed FDA Statement of Investigator (FDA Form 1572) for each principal investigator and co-principal investigator;
- Current curriculum vitae for the principal investigator, co-principal investigators, and subinvestigators listed on 1572s;
- Current and dated laboratory reference ranges for any laboratory listed on 1572s;
- Current and dated laboratory certifications for any laboratory listed on 1572s;
- IRB or EC approvals of the study protocol; amendments/addenda, when applicable; ICDs; and advertisements used to recruit patients, if applicable. IRB/EC approvals should include specific reference to document approved, the formal name of the IRB/EC issuing the approval, and the signature of the chairperson of the IRB/EC or designate. If the IRB/EC reviewed a specific outline or abridgment of the protocol prepared by the investigator instead of the complete protocol, a copy of the document actually reviewed should also be supplied to Parke-Davis.
- Signed CSA

2.1.1.2. Studies Not Conducted Under a US Investigational New Drug (IND) Application

- Signed protocol (and amendments/addenda, if applicable);
- IRB/EC approvals of the study protocol; amendments/addenda, when applicable; ICDs; and advertisements used to recruits study subjects/patients, if applicable (for requirements for IRB/EC approvals, see Section 2.1.1.1);

- Regulatory approval of protocol (and amendments/addenda), if dictated by local law;
- Current curriculum vitae for each principal investigator and co-principal investigator (if cannot be provided prior to study initiation, provide before end of study);
- Current laboratory reference ranges and certifications (if cannot be provided prior to study initiation, provide before end of study); and
- Signed Clinical Study Agreement

2.1.2. During the Study

- Revisions or updates to any documents listed in Section 2.1.1;
- Completed CRFs for each subject/patient entered into the study;
- Reports of all serious adverse events and pregnancies and any reports to the IRB/EC describing serious adverse events or deaths either caused by or during use of the investigational drug; and
- Investigator's annual progress report to the IRB/EC, and a copy of the annual IRB/EC reapproval of the study upon which the report is based, if dictated by local law.

2.1.3. End-of-Study

- Further revisions or updates to any documents listed in Section 2.1.1;
- Completed subject/patient CRFs not yet retrieved;
- Investigator's final report of the study, if applicable;
- Investigator's written notification to IRB/EC regarding study completion or discontinuation, if dictated by local law;
- Signed Statement of Clinical Study Discontinuance;
- Signed Investigator Authorization of Data Clarification Letter.

2.1.4. Retention of Study Records

In order to comply with GCP requirements, the investigator must maintain at the site for at least 15 years after the study ends the Master Patient Log that identifies all subjects/patients entered into the study such that each subject/patient can be identified by audit.

The investigator must maintain all study documents and adequate records pertaining to subject/patient files and other source data (eg, hospital files, consultation records, laboratory reports, electrocardiogram tracings, etc) for a minimum of 2 years after the IND is withdrawn or until 2 years after the NDA is either approved or withdrawn for the proposed indication of a clinical protocol. Other countries (eg, European Union, Nordic)

may require longer records retention for study sites within their countries (eg, at least 15 years after trial completion). For studies on drugs with approved indications in the US, documents have to be retained for a minimum of 3 years after trial completion.

The investigator must contact Parke-Davis for approval prior to discarding any study-related documents, even if retention requirements have been met.

If the principal investigator leaves the institution at which the study has been conducted, he/she or current representative must contact Parke-Davis to make suitable arrangements to ensure that the study records, including a copy of the master subject/patient log, are retained as specified above and to provide for the continuing access to the records by Parke-Davis personnel and regulatory authorities.

2.2. Guidelines for Recording Data in the Case Report Forms

The completed CRF is a legal document as it is intended for submission to a federal regulatory agency as part of a regulatory submission. Therefore, the following guidelines must be followed in its completion.

- All data entered on the CRF must be in ink (not red). No data entry in the original CRF may be deleted or corrected by either erasure, use of ink eradication fluid, liquid paper, adhesive correction tape, or any other means. When a data entry is in error, draw a single line through the erroneous entry (the original data must remain discernible) and indicate the correct data in whatever way is appropriate. All correction(s) with reason(s) for corrections (eg, entry error) must be initialed and dated by 1 of the investigators or the study coordinator. Usually the initials should be near the corrected data, clearly associated with the specific correction being made;
- Validity of data recorded on CRFs during each subject/patient visit will be attested to by a dated signature. As indicated on each CRF, some must be signed by an investigator, others can be signed by a study coordinator;
- All questions should be answered. If information cannot be provided, appropriately enter or mark a single line; NA for Not Applicable/Available; ND for Not Done; or UNK for Unknown;
- Each page of the CRF must contain the subject's/patient's initials, the subject's/patient's ID number, as well as the study number in the spaces provided. In the interest of subject/patient privacy, initials rather than full names should be used for identification. For the same reason, the social security number, address, or home telephone number of a subject/patient should not be entered in the CRF;

- The investigator is responsible for providing the completed original CRF for each subject/patient to the Parke-Davis Site Monitor. If the subject/patient is hospitalized, the information in the CRF may be compared with the subject's/patient's hospital records by the Parke-Davis Site Monitor to verify its accuracy. If, because of institutional policy, only a copy of certain hospital records can be included in the CRF, the copy must be completely legible and either be signed or initialed in ink by the investigator. Since this verified copy will be considered as the original by Parke-Davis, the investigator is responsible for informing Parke-Davis of any change that may be made in the "true" original that will be in the subject's/patient's hospital records.

2.3. Review of Case Report Forms

The completed CRFs will be reviewed by Parke-Davis Clinical/Medical Colleagues, Site Monitors, Biometrics, Clinical Data Coordination, and/or Clinical Communications colleagues, or equivalent persons in a designated Contract Research Organization. The investigator will be contacted if any corrections or additions are necessary. The investigator is responsible for cooperating fully with Parke-Davis personnel or its designee in correcting any erroneous or contradictory data entries.

2.4. Investigator's Responsibility for Clinical Drug Supply Accountability

All clinical drug supplies, ie, new or marketed drug, any corresponding placebo, and any active drug control (including marketed formulations) in finished dosage form, provided by Parke-Davis to the investigator for use in the clinical study must be accounted for in written documentation (i.e., records of receipt, dispensing, and return/destruction) that must be maintained and retained by the investigator and that will be monitored by Parke-Davis personnel. Note: if applicable, the investigator may designate a pharmacist to be responsible for clinical drug supply accountability.

2.4.1. Receipt of Clinical Drug Supplies

The investigator must verify and acknowledge the receipt of clinical drug supplies and retain related documentation (eg, Investigational Drug Release Authorization form).

2.4.2. Storage

Clinical drug supplies must be maintained as specified in the protocol (eg, environmental conditions required for stability) under secure (locked) conditions. Access to the stored study medication should be limited to the investigators, the study coordinator, and the pharmacist (when applicable).

2.4.3. Dispensing of Clinical Drug Supplies

Medication Dispensing Record forms will be provided with the CRFs. The investigator must assure completion of MDRs with appropriate information (eg, tear-off labels, date dispensed and returned by the patient, etc). A copy of these records must be provided to Parke-Davis as part of the close-out for the study. The drug accountability records must be retained by the investigator along with the subject's/patient's study records.

2.4.4. Return of Clinical Drug Supplies

As specified in the protocol, all medication containers and all unused medication remaining at the termination or completion of the study must be returned to the address shown in the protocol or with the drug shipment.

Used medication containers should be maintained separately and returned periodically during the trial. The Parke-Davis Site Monitor will assist in returning medication and containers as required. The investigator must document on appropriate forms numbers of containers returned. All returned medication provided by Parke-Davis will be counted by Clinical Pharmaceutical Operations as part of the study quality assurance audit and to meet regulatory requirements.

When applicable, any on-site drug destruction must be pre-approved by Parke-Davis, and actual drug destruction must be documented.

3. PROTOCOL AMENDMENTS AND ADDENDA

Definitions

A *protocol amendment* is any systematic change (eg, revision, addition, deletion) that is made to the Final Protocol for all sites from a clinical study and is identified by consecutive Arabic numerals (eg, Amendment 1, Amendment 2, etc). Amendments can be made regardless of whether the protocol has been signed by the investigator or whether or not the protocol has been implemented at a site.

A *Protocol Addendum* is any systematic change (eg, revision, addition, deletion) that is made to the Final Protocol for one/some site(s) but not all sites from a multicenter clinical study and is identified by single sequentially ordered letters (eg, Addendum A, Addendum B).

An *Urgent Protocol Amendment* is one that must be instituted quickly, usually to eliminate an apparent immediate hazard to subjects and may be implemented prior to eventual IRB/EC review (within 5 working days) and submission to regulatory authorities.

An *Amended Protocol* is a working copy of the Final Protocol that incorporates changes described in protocol amendments only that affect study management at the site, is generally created for use by the clinical investigator and staff, and is distributed to the sites concurrent with amendments.

All amendments/addenda to the protocol must be approved by the principal investigator, the Parke-Davis Clinical/Medical Colleague, the Statistician, and the IRB/EC of the investigator's institution. The investigator is responsible for submitting any proposed change in the approved protocol in writing to the IRB/EC for review and approval and for sending a copy of the approval to Parke-Davis. All amendments/addenda will be filed with the FDA or appropriate regulatory authorities by the Regulatory Affairs Departments of Parke-Davis.

With the exception indicated in Section 3.1 below, the amendment/addendum will apply to all subjects/patients entered into the study (or all subjects/patients in affected sites for addenda) after it has gone through the applicable procedure described above and been approved by the IRB/EC. Any amendments/addenda proposed in a multicenter protocol must be approved by the IRB/EC at the individual study site before it can be placed in effect at that site.

Protocol modifications or amendments/addenda fall into 1 of the following categories.

3.1. Urgent Protocol Amendment

If the amendment eliminates an apparent immediate safety hazard to the subject/patient (urgent protocol amendment), it may be implemented immediately. Parke-Davis will promptly notify the FDA and/or appropriate regulatory authorities of the amendment while the investigator will notify his/her IRB/EC of the change in writing within 5 working days of its implementation.

3.2. Other Amendments/Addenda

Examples of protocol modifications requiring amendments/addenda to the Final Protocol and thus prior IRB/EC approval include:

- Changes in the drug dosage or formulation;
- Increases in subjects'/patients' duration of exposure to drug;
- Increases in subject/patient numbers;
- Significant changes in protocol design (eg, drop/add control group)
- Addition of test/procedure that better monitors/reduces risk of side effects/adverse events;
- Elimination of test intended to monitor safety;

- Specific request from a regulatory agency;
- Modification in entry (ie, inclusion/exclusion) or evaluability criteria;
- Significant change in safety/efficacy status of the study drug;
- Ambiguity (scientific or grammatical) in the protocol that needs clarification;
- Changes to the statistical analysis plan.

3.3. Study Termination

The study will normally be carried to completion as described in the protocol. However, if in the course of the study a severe adverse reaction or intercurrent illness is noted in any subject/patient, consideration may be given to abrupt termination of the study for this patient. Such a decision may be made by either the principal investigator or by the Parke-Davis Clinical/Medical Colleague, or both.

Parke-Davis may elect to terminate the entire study at any time for any reasonable cause. If the entire study is terminated, all investigators will be notified immediately. Parke-Davis may elect to terminate the study at a specific site at any time for any reasonable cause, after consultation with the investigator.

APPENDIX F

The Declaration of Helsinki

APPENDIX F

The Declaration of Helsinki

Recommendations Guiding Physicians in Biomedical Research
Involving Human Subjects

Adopted by the 18th World Medical Assembly
Helsinki, Finland, June 1964,

and amended by
the 29th World Medical Assembly, Tokyo, Japan, October 1975;
the 35th World Medical Assembly, Venice, Italy, October 1983;
the 41st World Medical Assembly, Hong Kong, September 1989;

and the

48th General Assembly, Somerset West, Republic of South Africa, October 1996

INTRODUCTION

It is the mission of the physician to safeguard the health of the people. His or her knowledge and conscience are dedicated to the fulfillment of this mission.

The Declaration of Geneva of the World Medical Association binds the physician with the words, "The health of my patient will be my first consideration," and the International Code of Medical Ethics declares that, "A physician shall act only in the patient's interest when providing medical care which might have the effect of weakening the physical and mental condition of the patients."

The purpose of biomedical research involving human subjects must be to improve diagnostic, therapeutic, and prophylactic procedures and the understanding of the etiology and pathogenesis of disease.

In current medical practice most diagnostic, therapeutic, or prophylactic procedures involve hazards. This applies especially to biomedical research.

Medical progress is based on research which ultimately must rest in part on experimentation involving human subjects.

In the field of biomedical research a fundamental distinction must be recognized between medical research in which the aim is essentially diagnostic or therapeutic for a patient, and

medical research, the essential object of which is purely scientific and without implying direct diagnostic or therapeutic value to the person subjected to the research.

Special caution must be exercised in the conduct of research which may affect the environment, and the welfare of animals used for research must be respected.

Because it is essential that the results of laboratory experiments be applied to human beings to further scientific knowledge and to help suffering humanity, the World Medical Association has prepared the following recommendations as a guide to every physician in biomedical research involving human subjects. They should be kept under review in the future. It must be stressed that the standards as drafted are only a guide to physicians all over the world. Physicians are not relieved from criminal, civil, and ethical responsibilities under the laws of their own countries.

I. BASIC PRINCIPLES

A. Biomedical research involving human subjects must conform to generally accepted scientific principles and should be based on adequately performed laboratory and animal experimentation and on a thorough knowledge of the scientific literature.

B. The design and performance of each experimental procedure involving human subjects should be clearly formulated in an experimental protocol which should be transmitted for consideration, comment, and guidance to a specially appointed committee independent of the investigator and the sponsor provided that this independent committee is in conformity with the laws and regulations of the country in which the research experiment is performed.

C. Biomedical research involving human subjects should be conducted only by scientifically qualified persons and under the supervision of a clinically competent medical person. The responsibility for the human subject must always rest with a medically qualified person and never rest on the subject of the research, even though the subject has given his or her consent.

D. Biomedical research involving human subjects cannot legitimately be carried out unless the importance of the objective is in proportion to the inherent risk to the subject.

E. Every biomedical research project involving human subjects should be preceded by careful assessment of predictable risks in comparison with foreseeable benefits to the subject or to others. Concern for the interests of the subject must always prevail over the interests of science and society.

F. The right of the research subject to safeguard his or her integrity must always be respected. Every precaution should be taken to respect the privacy of the subject and to minimize the impact of the study on the subject's physical and mental integrity and on the personality of the subject.

G. Physicians should abstain from engaging in research projects involving human subjects unless they are satisfied that the hazards involved are believed to be predictable. Physicians should cease any investigation if the hazards are found to outweigh the potential benefits.

H. In publication of the results of his or her research, the physician is obliged to preserve the accuracy of the results. Reports of experimentation not in accordance with the principles laid down in this Declaration should not be accepted for publication.

I. In any research on human beings, each potential subject must be adequately informed of the aims, methods, anticipated benefits, and potential hazards of the study and the discomfort it may entail. He or she should be informed that he or she is at liberty to abstain from participation in the study and that he or she is free to withdraw his or her consent to participation at any time. The physician should then obtain the subject's freely-given informed consent, preferably in writing.

J. When obtaining informed consent for the research project, the physician should be particularly cautious if the subject is in a dependent relationship to him or her or may consent under duress. In that case the informed consent should be obtained by a physician who is not engaged in the investigation and who is completely independent of this official relationship.

K. In case of legal incompetence, informed consent should be obtained from the legal guardian in accordance with national legislation. Where physical or mental incapacity makes it impossible to obtain informed consent, or when the subject is a minor, permission from the responsible relative replaces that of the subject in accordance with national legislation.

Whenever the minor child is in fact able to give a consent, the minor's consent must be obtained in addition to the consent of the minor's legal guardian.

L. The research protocol should always contain a statement of the ethical considerations involved and should indicate that the principles enunciated in the present Declaration are complied with.

II. MEDICAL RESEARCH COMBINED WITH PROFESSIONAL CARE (Clinical Research)

A. In the treatment of the sick person, the physician must be free to use a new diagnostic and therapeutic measure, if in his or her judgment it offers hope of saving life, reestablishing health, or alleviating suffering.

B. The potential benefits, hazards, and discomfort of a new method should be weighed against the advantages of the best current diagnostic and therapeutic methods.

C. In any medical study, every patient - including those of a control group, if any - should be assured of the best proven diagnostic and therapeutic methods. This does not exclude the use of inert placebo in studies where no proven diagnostic or therapeutic method exists.

D. The refusal of the patient to participate in a study must never interfere with the physician-patient relationship.

E. If the physician considers it essential not to obtain informed consent, the specific reasons for this proposal should be stated in the experimental protocol for transmission to the independent committee.(I,B)

F. The physician can combine medical research with professional care, the objective being the acquisition of new medical knowledge, only to the extent that medical research is justified by its potential diagnostic or therapeutic value for the patient.

III. NONTHERAPEUTIC BIOMEDICAL RESEARCH INVOLVING HUMAN SUBJECTS (Non-Clinical Biomedical Research)

A. In the purely scientific application of medical research carried out on a human being, it is the duty of the physician to remain the protector of the life and health of that person on whom biomedical research is being carried out.

B. The subjects should be volunteers - either healthy persons or patients for whom the experimental design is not related to the patient's illness.

C. The investigator or the investigating team should discontinue the research if in his/her or their judgment it may, if continued, be harmful to the individual.

D. In research on man, the interest of science and society should never take precedence over considerations related to the well-being of the subject.

Appendix G

Amendment 1 - October 6, 1998

APPENDIX G

Amendment 1 - October 6, 1998

Amended--

OLD >

LABORATORY(IES): [redacted]

October 6, 1998 - Amendment 1

NEW>

LABORATORY(IES): [redacted]

Amended--

OLD>

6 STUDY DESIGN AND METHODOLOGY 9
6.1 Study Design 9
6.2 Study Schedule and Guidelines for Visits 9
6.2.1. Screening Visit (Visit 1) 9
6.2.2. Atorvastatin 10 mg Run-In Period 10
6.2.3. Double-Blind Active Treatment Period 12
6.3. Efficacy Assessments 12
6.3.1. Primary Efficacy Parameters 13
6.3.2. Secondary Efficacy Parameters 13
6.3.3. End Point Committee 13
6.3.4. Clinical End Point Documentation 14
6.4. Safety Assessments 14
6.4.1. Laboratory Evaluation 14

October 6, 1998 - Amendment 1

NEW>

6. STUDY DESIGN AND METHODOLOGY 9
6.1. Study Design 9
6.2. Study Schedule and Guidelines for Visits 9
6.2.1. Screening Visit (Visit 1) 9
6.2.2. Atorvastatin 10 mg Run-InPeriod 10
6.2.3. Rescreening and Retesting Prior to Randomization 12
6.2.4. Double-Blind Active Treatment Period 12
6.3. Efficacy Assessments 12
6.3.1. Primary Efficacy Parameters 13
6.3.2. Secondary Efficacy Parameters 13

6.3.3. End Point Committee 13
6.3.4. Clinical End Point Documentation 14
6.4. Safety Assessments 14
6.4.1. Laboratory Evaluation 14

Amended--

OLD>

12. LIST OF APPENDICES

A. Timetable of Visits and Procedures 33
B. Commonly Prescribed Prohibited Medications 41
C. Study Safety Procedures and Clinical Laboratory Determinations 45
D. Administrative Procedures and Clinical Laboratory Determinations 51
E. Other Administrative and Regulatory Procedures 59
F. Declaration of Helsinki 74

<u>October 6, 1998 - Amendment 1</u>

NEW>

12. LIST OF APPENDICES

A. Timetable of Visits and Procedures 33
B. Commonly Prescribed Prohibited Medications 41
C. Study Safety Procedures and Clinical Laboratory Determinations 45
D. Administrative Procedures and Clinical Laboratory Determinations D1
E. Other Administrative and Regulatory Procedures E1
F. Declaration of Helsinki F1
G. Amendments - September 28, 1998 .... G1

Amended--

OLD>

5.2.1. Inclusion Criteria

<u>At Screening</u>:

1. Men or women. Women of child-bearing potential must be using adequate measures of contraception (as determined by the investigator) to avoid pregnancy and should be highly unlikely to conceive during the study period.

<u>October 6, 1998 - Amendment 1</u>

NEW>

5.2.1. Inclusion Criteria

<u>At Screening</u>:

2. Men or women. Women of child-bearing potential must be using adequate measures of contraception (as determined by the investigator) to avoid pregnancy and should be highly unlikely to conceive during the study period. *Women of child-bearing potential must have a negative pregnancy test.*

Amended---

OLD>

(5.2.1 Inclusion Criteria)

a) prior myocardial infarction;
b) prior or present angina with objective evidence of atherosclerotic CHD (see Appendix A5.
c) prior coronary revascularization procedure (e.g., PTCA, CABG).

<u>October 6, 1998 - Amendment 1</u>

NEW>

(5.2.1 Inclusion Criteria)

a) prior myocardial infarction;
b) prior or present angina with objective evidence of atherosclerotic CHD (see Appendix A5). *As a surrogate for unstable /severe angina, patients qualifying based on this criterion alone (i.e., no prior MI or revascularization) must have been hospitalized for the treatment of angina, and not for diagnostic work alone, 1-60 months prior to screening. The hospitalization must be for an overnight stay.*
c) prior coronary revascularization procedure (e.g., PTCA, CABG).

Amended---

OLD>

(5.2.2 Exclusion Criteria)

3. Evidence of other uncontrolled or concurrent conditions or medications that may obscure the efficacy or safety comparisons:
   a) uncontrolled hypertension (investigator defined) at the screening visit
   b) a myocardial infarction, coronary angioplasty, coronary artery bypass graft, other revascularization procedure, or severe/unstable angina or other hospitalization for an atherosclerotic condition within one month of the screening visit
   c) any planned surgical procedure for the treatment of atherosclerosis
   d) an ejection fraction (if known) of <30%
   e) hemodynamical important valvular disease
   f) GI disease limiting drug absorption or, partial ileal bypass
   g) any non-skin malignancy, malignant melanoma, or other survival limiting disease (including class IIIb/IV congestive heart failure, life-threatening arrhythmias and candidates for organ transplant).
   h) a CPK > 6 times the upper limit of normal (at visit 1) that is unexplained
   i) other significant abnormalities that the investigator feels may compromise the patient's safety or successful participation in the study
   j) patients taking any of the prohibited medications specified in Section 5.3 (below). Patients with concurrent conditions that require frequent use of prohibited medications should be excluded.

<u>October 6, 1998 - Amendment 1</u>

NEW>

(5.2.2 Exclusion Criteria)

3. Evidence of other uncontrolled or concurrent conditions or medications that may obscure the efficacy or safety comparisons:
   a) uncontrolled hypertension (investigator defined) at the screening visit
   b) a myocardial infarction, coronary angioplasty, coronary artery bypass graft, other revascularization procedure, or severe/unstable angina or other hospitalization for an atherosclerotic condition within one month of the screening visit
   c) any planned surgical procedure for the treatment of atherosclerosis
   d) an ejection fraction (if known) of <30%

<u>Continued - NEW</u>

   e) hemodynamically important valvular disease
   f) GI disease limiting drug absorption or, partial ileal bypass

g) any non-skin malignancy, malignant melanoma, or other survival limiting disease (including class IIIb/IV congestive heart failure, life-threatening arrhythmias and candidates for organ transplant). *Patients with a history of the above cancers are not eligible if any treatment (medical, surgical, radiological) and/or recurrence was clinically evident in the 10 years prior to screening.*
h) a CPK > 6 times the upper limit of normal (at visit 1) that is unexplained
i) other significant abnormalities that the investigator feels may compromise the patient's safety or successful participation in the study
j) patients taking any of the prohibited medications specified in Section 5.3 (below). Patients with concurrent conditions that require frequent use of prohibited medications should be excluded.

Amended---
OLD>
(5.3 Prohibited Medications or Precautions)

- Patients taking a lipid-altering drug(s) must have the medication discontinued at or prior to the screening visit to be eligible for screening, except in the case of probucol where medication must have been discontinued for at least 12 months.

October 6, 1998 - Amendment 1
NEW>
(5.3 Prohibited Medications or Precautions)

- Patients taking a lipid-*regulating* drug(s) must have the medication discontinued at or prior to the screening visit to be eligible for screening, except in the case of probucol where medication must have been discontinued for at least 12 months.

Amended---
OLD>
(5.3 Prohibited Medications or Precautions)

- Other drugs known to be associated with an increased risk of rhabdomyolysis in combination with HMG-CoA reductase inhibitors [e.g., erythromycin, azole antifungals (systemic or topical) or mibefradil dihydrochloride].

October 6, 1998 - Amendment 1
NEW>
(5.3 Prohibited Medications or Precautions)

- Other drugs known to be associated with an increased risk of rhabdomyolysis in combination with HMG-CoA reductase inhibitors [including: erythromycin, *any chronic systemic antifungal use, mibefradil dihydrochloride, nefazodone, fluvoxamine and other potent inhibitors of cytochrome P450-3A4]*.

Amended---
OLD>
(5.3 Prohibited Medications or Precautions)
Systemic steroids (long term use is prohibited, i.e., ≥2 weeks)

October 6, 1998 - Amendment 1
NEW>
(5.3 Prohibited Medications or Precautions)
<Letter/Sentence/Paragraph Deleted>

Amended---
OLD>
(5.3 Prohibited Medications or Precautions)
Should these medications be necessary during the course of the trial, consideration should be given to temporarily withholding study drug and restarting it only after time has elapsed to allow for sufficient clearance of the medication. In addition, temporarily withholding study drug should be considered in any patient with a risk factor predisposing to the development of renal failure secondary to

rhabdomyolysis, including severe acute infection; hypertension; major surgery; trauma; severe metabolic, endocrine, or electrolyte disorders; and uncontrolled seizures.

October 6, 1998 - Amendment 1

NEW>

(5.3 Prohibited Medications or Precautions)

Should these medications be necessary during the course of the trial, consideration should be given to temporarily withholding study drug and restarting it only after time has elapsed to allow for sufficient clearance of the medication. In addition, temporarily withholding study drug should be considered in any patient with a risk factor predisposing to the development of renal failure secondary to rhabdomyolysis, including severe acute infection; *hypotension;* major surgery; trauma; severe metabolic, endocrine, or electrolyte disorders; and uncontrolled seizures.

Amended--

OLD>

(5.3 Prohibited Medications or Precautions)

Cocomitant use with digoxin and/or coumarin anti-coagulants requires appropriate monitoring

October 6, 1998 - Amendment 1

NEW>

(5.3 Prohibited Medications or Precautions)

As co-administration of atorvastatin (80 mg) and digoxin increased steady-state plasma digoxin concentrations by approximately 20%, patients taking digoxin should be monitored appropriately.

While atorvastatin had no clinically significant effect on prothrombin time when administered to patients receiving chronic warfarin therapy, prothrombin time should be monitored as clinically appropriate.

Amended--

OLD>

5.4 Allowable Concurrent Medications

The dosage and regimen of any chronic, permitted concurrent medications (e.g., calcium channel blockers, b-blockers, hormone replacement therapy, hormone contraceptives, thiazides, loop diuretics, H-2 antagonists, aspirin, phenytoin) should be stabilized (for at least 30 days) before the open-label run-in phase begins (Visit 2) and kept constant (as is medically appropriate) throughout the study. Any medications prescribed chronically or intermittently during the study or dose adjustments of these medications must be reported on the concurrent medication Case Report Form (CRF). The occasional (< 3 times per week) use of antacids is permitted.

October 6, 1998 - Amendment 1

NEW>

5.5 Allowable Concurrent Medications

The dosage and regimen of any chronic, permitted concurrent medications (e.g., calcium channel blockers, b-blockers, hormone replacement therapy, hormone contraceptives, thiazides, loop diuretics, H-2 antagonists, aspirin, phenytoin) should be stabilized (for at least 30 days) before the open-label run-in phase begins (Visit 2) and kept constant (as is medically appropriate) throughout the study. Any medications prescribed chronically or intermittently during the study *<phrase deleted - dose adjustment>* must be reported on the concurrent medication Case Report Form (CRF). The occasional (< 3 times per week) use of antacids is permitted.

Amended--

OLD>

6.2.1. Screening Visit (Visit 1)

This single visit allows for an initial assessment of those patients the investigator feels may eventually qualify for randomization into the study. At screening an informed consent will be signed, demographic

characteristics assessed, vital signs measured, all concomitant medications documented, and a medical history recorded. The investigator should screen for potential causes of secondary hyperlipidemia. These should be identified and corrected, with laboratory verification, prior to Visit 2. TSH and HbA_{1c} measurements (for known or suspected diabetics) are obtained at Visit 1 for this purpose. Patients who appear to meet the demographic and medical history criteria should stop any prohibited medications at this visit (see section 5.3). Patients will have blood drawn for fasting lipid determinations and a standard clinical profile. LDL-C will be derived from the lipid profile (See Appendix A4 for methodology) in order to help the investigator project whether the patient will meet the inclusion criteria to enter the open-label run-in phase after dietary stabilization.

October 6, 1998 - Amendment 1

NEW>

6.2.1. Screening Visit (Visit 1)

This single visit allows for an initial assessment of those patients the investigator feels may eventually qualify for randomization into the study. At screening an informed consent will be signed, demographic characteristics assessed, vital signs measured, all concomitant medications documented, and a medical history recorded. The investigator should screen for potential causes of secondary hyperlipidemia. These should be identified and corrected, with laboratory verification, prior to Visit 2. TSH and HbA_{1c} measurements (for known or suspected diabetics) are obtained at Visit 1 for this purpose. Patients who appear to meet the demographic and medical history criteria should stop any prohibited medications at this visit (see section 5.3). Patients will have blood drawn for fasting lipid determinations and a standard clinical profile. LDL-C will be derived from the lipid profile (See Appendix A4 for methodology) in order to help the investigator project whether the patient will meet the inclusion criteria to enter the open-label run-in phase after dietary stabilization.

The CHD entry criteria must be documented (prior to Visit 2) through either the investigational site's medical records, or copies of such records from other institutions, or a letter from a referring physician that specifically states the diagnosis and date of the most recent occurrence of the qualifying event.

Amended---

OLD>

NOTE: Patients who, after the screening visit but before randomization, experience a cardiovascular event (e.g., non-fatal MI) or develop an exclusionary condition (e.g., systemic malignancy) must be discontinued and re-evaluated for eligibility according to the timing and criteria of the screening visit.

October 6, 1998 - Amendment 1

NEW>

NOTE: Patients who, after the screening visit but before randomization, experience a cardiovascular event (e.g., non-fatal MI) or develop an exclusionary condition (e.g., systemic malignancy) must be discontinued and re-evaluated for eligibility according to the timing and criteria of the screening visit *(see section 6.2.3 below).*

Amended---

OLD>

6.2.3 **Double-Blind Active Treatment Period**

This period begins at Visit 5 (Week 0). Procedures performed at this visit include an assessment of vital signs, an ECG and physical exam, repeat clinical chemistry/fasting lipid determinations and continued dietary counseling. The ECG and physical exam may be performed between Visits 4 and 5 once a patient's lipid levels qualify them for randomization.

The open-label atorvastatin 10 mg therapy will stop at Visit 5. Qualified patients will be randomized to 1 of 2 blinded treatment groups, either 80 mg atorvastatin or 10 mg atorvastatin. The patient should take the study medication once daily. Neither the investigator nor the study sponsor will be informed of the lipid values of randomized patients (except as noted in section 6.4.1) in order to maintain the blind of the study.

October 6, 1998 - Amendment 1
NEW>

6.2.3 Rescreening and Retesting Prior to Randomization

Patients may be rescreened once for the study if they experience a condition prior to randomization that makes them ineligible to continue in the study. The condition will need to be treated/stablized (e.g., uncontrolled diabetes, hypothyroidism, experiencing a potential endpoint) prior to consideration for rescreening. Rescreening of a patient must have prior approval of the Parke-Davis or Pfizer medical monitor or their designee.

Patients may be retested during the pre-randomization period only once for laboratory values that make them ineligible for the study (e.g. exclusionary levels of CPK and ALT/AST). Retesting will not be allowed for lipid eligibility at Visits 2, 3, or 4. Patients failing to meet eligibility based on retesting must be discontinued and are not eligible for rescreening. Retesting of a patient must have prior approval of the Parke-Davis or Pfizer medical monitor or their designee.

Amended--
OLD>

6.2.3 Double-Blind Active Treatment Period

October 6, 1998 - Amendment 1
NEW>

6.2.4 Double-Blind Active Treatment Period

Amended--
OLD>
(6.4.1.1 Clinically Important Laboratory Abnormalities)
Following normalization of a clinically important laboratory abnormality, the patient may be rechallenged with study medication. Rechallenges must be discussed with, and approved by, the Parke-Davis or Pfizer clinical monitor (or their designee) and appropriate IRB/EC. These patients will maintain their original treatment group assignment during rechallenge.

October 6, 1998 - Amendment 1
NEW>
(6.4.1.1 Clinically Important Laboratory Abnormalities)
Following normalization of a clinically important laboratory abnormality, the patient may be rechallenged with study medication. Rechallenges must be discussed with, and approved by, the Parke-Davis or Pfizer clinical monitor (or their designee) and appropriate IRB/EC. These patients will maintain their original treatment group assignment during rechallenge. *A rechallenge is defined as reinstitution of study drug following discontinuation for a medically significant adverse event considered to be caused by atorvastatin.*

Amended--

OLD>

APPENDIX A. 1
TIMETABLE OF CLINIC VISITS AND PROCEDURES

Study Period	Screening	Atorvastatin 10 mg ± Add-On			Double-Blind Treatment ± Add-On						
Visit #	1	2	3	4	5	6	7	8	9	10,12,14,16,18[i]	11,13,15,17,19[i]
study week		wk -8	wk -4	wk -2	wk 0	wk 12					
study month							mo 6	mo 9	mo 12	mos. 18, 30, 42,54, 66	mos. 24, 36, 48, 60, 72
**PROCEDURE**											
Informed Consent	X										
Demographics/ Med. History	X										
Vital Signs[a]	X	X	X	X	X	X	X	X	X	X	X
Clinical Labs[b,c]	X				X				X		X
Lipid Labs[d]	X	X	X	X	X[e]	X			X[e]		X
Safety Labs[f]				X		X					
Dietary Counseling	X----	----------throughout trial----------									----------X
Physical Exam				X[g]----	----X[g]				X		X
ECG				X[g]----	----X[g]				X		X
Dispense Drug		X[h]			X	X	X	X	X	X	X
Concom. Therapy	X	X	X	X	X	X	X	X	X	X	X
Adverse Events		X	X	X	X	X	X	X	X	X	X
End Points[j]		X	X	X	X	X	X	X	X	X	X

[a] height (screen only), weight, blood pressure

[b] serum chemistry and hematology by central laboratory and uninalysis at local site (see Appendix C.4.)

[c] includes TSH and HbA1C at screening, and the HbA1C annually beginning at Visit 9 (known/suspected diabetics only)

[d] includes total cholesterol, triglycerides, LDL-C, HDL-C

[e] includes apolipoprotein A1, B

[f] includes transaminases (ALT, AST) and CPK if clinically indicated

[g] can be done between Visit 4 or 5 once a patient's lipid levels qualify them for randomization

[h] open label atorvastatin 10 mg

[i] the semi-annual visit schedule beginning with Visits 10 and 11 will extend beyond month 72 for patients beyond this time point if the trial has not yet been stopped

[j] includes resource utilization for economic analyses

October 6, 1998 - Amendment 1
NEW>

APPENDIX A. 1
TIMETABLE OF CLINIC VISITS AND PROCEDURES

Study Period	Screening	Atorvastatin 10 mg *<Add-on removed>*			Double-Blind Treatment *<Add-on removed>*						
Visit #	1	2	3	4	5	6	7	8	9	10,12,14,16,18[i]	11,13,15,17,19[i]
study week		wk -8	wk -4	wk -2	wk 0	wk 12					
study month							mo 6	mo 9	mo 12	mos. 18, 30, 42,54, 66	mos. 24, 36, 48, 60, 72
**PROCEDURE**											
Informed Consent	X										
Demographics/ Med. History	X										
Vital Signs[a]	X	X	X	X	X	X	X	X	X	X	X
Clinical Labs[b,c]	X				X				X		X
Lipid Labs[d]	X	X	X	X	X[e]	X			X[e]		X
Safety Labs[f]				X		X					
Dietary Counseling	X----	------------throughout trial------------									----------X
Physical Exam				X[g]----	----X[g]				X		X
ECG				X[g]----	----X[g]				X		X
Dispense Drug		X[h]			X	X	X	X	X	X	X
Concom. Therapy	X	X	X	X	X	X	X	X	X	X	X
Adverse Events		X	X	X	X	X	X	X	X	X	X
End Points[j]		X	X	X	X	X	X	X	X	X	X

[a] height (screen only), weight, blood pressure
[b] serum chemistry and hematology by central laboratory and urinalysis at local site (see Appendix C.4.)
[c] includes TSH, *pregnancy test (women of child-bearing potential), and* HbA1C at screening; pregnancy testing (women of child-bearing potential) and HbA1C annually (known/suspected diabetics only) beginning at Visit 9
[d] includes total cholesterol, triglycerides, LDL-C, HDL-C
[e] includes apolipoprotein A1, B
[f] includes transaminases (ALT, AST) and CPK if clinically indicated
[g] can be done between Visit 4 or 5 once a patient's lipid levels qualify them for randomization
[h] open label atorvastatin 10 mg
[i] the semi-annual visit schedule beginning with Visits 10 and 11 will extend beyond month 72 for patients beyond this time point if the trial has not yet been stopped
[j] includes resource utilization for economic analyses

Amended---

OLD>

(Appendix A.2 Study Procedures By Visit)

Visit 1

- Obtain signed informed consent
- Obtain Medical History
- Begin to assess the patient's eligibility for the study considering the protocol

inclusion/exclusion criteria such as presence of CHD, history of previous myocardial infarction, age, etc.

- Draw blood samples for a complete clinical laboratory determination (including HbA_{1c}), lipid profile, and hematology
- Vital signs
- Urinalysis
- Begin dietary counseling
- Concurrent medication
- Discontinue prior dyslipidemic medication (if applicable)

October 6, 1998 - Amendment 1

NEW>

(Appendix A.2 Study Procedures By Visit)

Visit 1

- Obtain signed informed consent
- Obtain Medical History
- Begin to assess the patient's eligibility for the study considering the protocol

inclusion/exclusion criteria such as presence of CHD, history of previous myocardial infarction, age, etc.

- Draw blood samples for a complete clinical laboratory determination (including HbA_{1c}), lipid profile, and hematology
- Vital signs
- Urinalysis
- Begin dietary counseling
- Concurrent medication
- Discontinue prior dyslipidemic medication (if applicable)
- *Pregnancy test for women of child-bearing potential*

Amended---

OLD>

(Appendix A.2 Study Procedures By Visit)

Visits 9,11,13,15,17,19 (and at 12 month intervals thereafter as required)

- Physical examination
- Draw blood samples for full clinical laboratory, lipid profile (special lipids at Visit 9 only), hematology, HbA1C (known/suspected diabetics), and plasma storage
- Vital signs
- Urinalysis
- ECG
- Continue dietary counseling
- Dispense study medication (not required at final visit)
- Record adverse events
- Record potential end points
- Record changes in and additions to concurrent medications

October 6, 1998 - Amendment 1
NEW>
(Appendix A.2 Study Procedures By Visit)
Visits 9,11,13,15,17,19 (and at 12 month intervals thereafter as required)

- Physical examination
- Draw blood samples for full clinical laboratory, lipid profile (special lipids at Visit 9 only), hematology, HbA1C (known/suspected diabetics), and plasma storage
- Vital signs
- Urinalysis
- ECG
- Continue dietary counseling
- Dispense study medication (not required at final visit)
- Record adverse events
- Record potential end points
- Record changes in and additions to concurrent medications
- *Pregnancy test for women of child-bearing potential*

Amended---
OLD>
(Appendix A.5 Objective Criteria for Atherosclerotic CHD)
Patient must have been hospitalized with a discharge diagnosis that includes angina, chest pain, or similar condition, and has documented evidence of at least one of the following:

1. chest pain with associated evidence of myocardial ischemia defined as either
   - electrocardiographic evidence
   - any positive myocardial stress test (e.g., thallium^{201})
   - any wall motion abnormality as detected on cardiac ultrasound
   - new coronary angiographic lesions of significant atherosclerotic lesions.

October 6, 1998 - Amendment 1
NEW>
(Appendix A.5 Objective Criteria for Atherosclerotic CHD)
Patient must have been hospitalized with a discharge diagnosis that includes angina, chest pain, or similar condition, and has documented evidence of at least one of the following:

1. chest pain with associated evidence of myocardial ischemia defined as either
   - electrocardiographic evidence
   - any positive myocardial stress test (e.g., thallium^{201})
   - any wall motion abnormality as detected on cardiac ultrasound
   - new coronary angiographic lesions of $\geq$ *50% stenosis in one or more epicardial vessels.*

Amended--

OLD>

(Appendix B Commonly Prescribed Prohibited Medicaitons)

Systemic Steroids (long term use is prohibited i.e., $\geq$ 2 weeks)

Generic Name	Market Name*	Form/Source	Manufacturer
Triamcinolone Acetonide	Kenalog-10	Sterile Injection	Westwood-Squibb
	Kenalog-40	Sterile Injection	Westwood-Squibb
Triamcinolone Diacetate	Aristocort	Sterile Injection	Fujisawa
	Aristospan	Sterile Injection	Fujisawa
Betamethasone Sodium Phosphate, Betamethasone Acetate	Celestone Soluspan	Sterile Injection	Schering
Hydrocortisone	Cortenema	Enema Solution	Solvay
	Hydrocortone	Tablets	Merck, Sharp and Dohme
Hydrocortisone Acetate	Hydrocortone Acetate	Sterile Injection	Merck, Sharp and Dohme
Hydrocortisone Sodium Phosphate	Hydrocortone Phosphate	Sterile Injection	Merck, Sharp and Dohme
Hydrocortisone Sodium Succinate	Solu-Cortef	Sterile Injection (IV)	Upjohn
Cortisone Acetate	Cortone Acetate	Sterile Injection	Merck, Sharp and Dohme
Dexamethasone	Decadron	Oral Elixir	Merck, Sharp and Dohme
Dexamethasone Acetate	Decadron - LA	Sterile Injection	Merck, Sharp and Dohme
Dexamethasone Sodium Phosphate	Decadron	Sterile Injection	Merck, Sharp and Dohme
		Tablets	Merck, Sharp and Dohme
Prednisone	Deltasone	Tablets	Upjohn
Methylprednisolone	Medrol	Tablets	Upjohn
Methylprednisolone Acetate	Depo-Medrol	Sterile Injection	Upjohn
Methylprednisolone Sodium Succinate	Solu-Medrol	Sterile Injection (IV)	Upjohn
Prednisolone Tebutate	Hydeltra-T.B.A.	Sterile Injection	Merck, Sharp, and Dohme
Prednisolone Sodium Phosphate	Hydeltrasol	Sterile Injection	Merck, Sharp, and Dohme
Methyltestosterone	Android-10	Tablet	ICN Pharmaceuticals
	Android-25		
	Metandren	Tablet	CIBA Pharmaceuticals
Fluoxymesterone	Halotestin	Tablet	Upjohn

October 6, 1998 - Amendment 1

NEW>

(Appendix B Commonly Prescribed Prohibited Medicaitons)

<Systemic Steroid Section Deleted>

Amended--

OLD>

(Appendix B Commonly Prescribed Prohibited Medications)

The following are also prohibited:

- anti-fungal agents (chronic systemic use)
- mibefradil dihydrochloride (Posicor®)
- fish oils

October 6, 1998 - Amendment 1
NEW>
(Appendix B Commonly Prescribed Prohibited Medications)

The following are also prohibited:

- any chronic systemic anti-fungal agent use
- mibefradil dihydrochloride (Posicor®)
- fish oils
- *nefazodone*
- *fluvoxamine*
- *concurrent use of other potent cytochrome P450-3A4 inhibitors*

Amended---

OLD>
(Appendix B Commonly Prescribed Prohibited Medicaitons)

Note: Concomitant use with digoxin and/or coumarin anti-coagulants requires appropriate monitoring.

October 6, 1998 - Amendment 1

NEW>
(Appendix B Commonly Prescribed Prohibited Medicaitons)

(Letter/Sentence/Paragraph Deleted)

Amended---

OLD>

APPENDIX C.2
ELECTROCARDIOGRAM

A 12-lead electrocardiogram will be recorded as scheduled with the patient recumbent and resting. The electrocardiogram will be interpreted by a central reading laboratory.

October 6, 1998 - Amendment 1

NEW>

APPENDIX C.2
ELECTROCARDIOGRAM

A 12-lead electrocardiogram will be recorded as scheduled with the patient recumbent and resting. *The electrocardiogram should be performed and submitted to [redacted]l using the procedure specified for routine ECGs.*

If a patient experiences a myocardial infarction, an ECG should be performed between 6 weeks and 3 months following the myocardial infarction. It may be done at a regularly scheduled visit if the visit is to occur within that timeframe. The ECG should be performed and submitted to [redacted]l using the procedure specified for routine ECGs.

Amended---

OLD>

(Appendix C.3 Routine Safety Profile)

Chemistry

SGOT (AST)
SGPT (ALT)
Alkaline Phosphatase
LDH

<u>Continued - OLD</u>

CPK if CPK elevated above 2 times the upper reference limit)
Blood Urea Nitrogen
Creatinine
Uric Acid
Total Protein
Albumin
Total Bilirubin
Sodium
Potassium
Glucose
Calcium
HbA_{1c}

<u>October 6, 1998 - Amendment 1</u>

NEW>

(Appendix C.3 Routine Safety Profile)

Chemistry

SGOT (AST)
SGPT (ALT)
Alkaline Phosphatase
LDH
CPK (*Troponin-T* if CPK elevated above 2 times the upper reference limit)
Blood Urea Nitrogen
Creatinine
Uric Acid
Total Protein
Albumin
Total Bilirubin
Sodium
Potassium
Glucose (HbA_{1c} if glucose >126 mg/dl)
Calcium
HbA_{1c}
TSH *(at Visit 1 only)*

Amended--

OLD>

APPENDIX D

ADMINISTRATIVE PROCEDURES FOR THE
REPORTING OF ADVERSE EVENTS

APPENDIX E

OTHER ADMINISTRATIVE AND REGULATORY PROCEDURES

APPENDIX F

THE DECLARATION OF HELSINKI

October 6, 1998 - Amendment 1

NEW>

New Appendices D, E, F are now attached via hard-copy and are not paginated electronically within this document.

Amended--

October 6, 1998 - Amendment 1

NEW>

APPENDIX G

Amendments - October 6, 1998

Appendix H

Amendment 2 - January 15, 1999

APPENDIX H

Amendment 2 - January 15, 1999

Amended--
OLD >
(Synopsis)
TOTAL PLANNED SAMPLE SIZE Approximately 35-60 patients at each of 150-250 centers for 8,600 randomized patients.

January 15, 1999 - Amendment 2
NEW>
(Synopsis)
TOTAL PLANNED SAMPLE SIZE ***A minimum of 35*** patients at each of 150-250 centers for 8,600 randomized patients.

Amended--
OLD >
(Title/Signature Pages)
October 6, 1998 - Amendment 1

[redacted]
[redacted]2

January 15, 1999 - Amendment 2
NEW>
(Title/Signature Pages)

[redacted]

Amended--
OLD >
(1. INTRODUCTION)
Preliminary results from the 9,000 patient Long-Term Intervention With Pravastatin in Ischemic Disease (LIPID) Study which utlized a similar population and treatment regimen to CARE, showed a 29% reduction in CHD. There was no statistically significant heterogeneity of the treatment effect among prespecified subgroups, including baseline LDL-C.[24]

January 15, 1999 - Amendment 2
NEW>
(1. INTRODUCTION)
Results from the *9,014* patient Long-Term Intervention With Pravastatin in Ischemic Disease (LIPID) Study which utlized a similar population and treatment regimen to CARE, showed a 29% reduction in CHD. There was no statistically significant heterogeneity of the treatment effect among prespecified subgroups, including baseline LDL-C, *with point estimates ranging from a 16% reduction to a 30% reduction in CHD for baseline LDL subgroups of <135 mg/dl and ≥ 174 mg/dl, respectively.*[24]

Amended---
OLD >
(5.1 Source and Number of Patients)
Source: Approximately 150-250 medical care facilities will participate. These centers will consist of providers of medical care treating patients with primary hyperlipidemia with coexistent coronary, cerebral, or peripheral vascular disease. Each center will be responsible for overseeing a total randomization of approximately 35-60 patients into 2 treatment arms: drug therapy to achieve an LDL-C goal of approximately 100 mg/dl (2.6 mmol/l) using atorvastatin 10 mg once daily or, an LDL-C goal approximately 25% below that using atorvastatin 80 mg once daily.

January 15, 1999 - Amendment 2
NEW>
(5.1 Source and Number of Patients)
Source: Approximately 150-250 medical care facilities will participate. These centers will consist of providers of medical care treating patients with primary hyperlipidemia with coexistent coronary, cerebral, or peripheral vascular disease. Each center will be responsible for overseeing a total randomization of ***at least 35*** patients into 2 treatment arms: drug therapy to achieve an LDL-C goal of approximately 100 mg/dl (2.6 mmol/l) using atorvastatin 10 mg once daily or, an LDL-C goal approximately 25% below that using atorvastatin 80 mg once daily.

Amended---
OLD >
(5.2.1 Inclusion Criteria)
3. have clinically evident CHD defined as at least one of the following clinical manifestations of coronary atherosclerosis. The most recent occurrence must be 1-60 months prior to screening.

a) prior myocardial infarction;

October 6, 1998 - Amendment 1

b) prior or present angina with objective evidence of atherosclerotic CHD (see Appendix A5). *As a surrogate for unstable/severe angina, patients qualifying based on this criterion alone (i.e., no prior MI or revascularization) must have been hospitalized for the treatment of angina, and not for diagnostic work alone, 1-60 months prior to screening. The hospitalizaiton must be for an overnight stay.*

c) prior coronary revascularization procedure (e.g., PTCA, CABG).

January 15, 1999 - Amendment 2
NEW>
(5.2.1 Inclusion Criteria)

3. ***Have clinically evident CHD defined as at least one of the following clinical manifestations of coronary atherosclerosis.***
   a) ***prior myocardial infarction 1-60 months prior to screening;***
   b) ***prior or present angina with objective evidence of atherosclerotic CHD. As a surrogate for unstable /severe angina, patients qualifying based on this criterion alone must have also been hospitalized with a discharge diagnosis of angina. The hospitalization must have been for management of angina and not for diagnostic work alone. The hospitalization must have occurred***

1-60 months prior to screening. Objective evidence of atherosclerotic CHD requires documentation of one or more of the following:

- ***A reversible defect on nuclear stress testing. The stress must be exercise, adenosine, dobutamine or dipyridamole. The imaging agent must be thallium, sestamibi, or tetrafosmin. Fixed defects or defects reported as "possible artifact" or "possible ischemia" do not meet this criterion.***
- ***A reversible wall motion abnormality on echocardiographic stress testing. The stress must be exercise, adenosine, dobutamine or dipyridamole. Fixed wall motion abnormalities, or abnormalities considered to be borderline, or possibly not due to ischemia, do not meet this criterion.***
- ***The demonstration by coronary angiography of a 50% or greater diameter stenosis in at least one of the major vessels (left main, LAD, circumflex, or dominant RCA). Stenoses in non-dominant RCA, or diagonal, marginal or posterolateral branches do not meet this criterion.***

c) prior coronary revascularization procedure as follows:
CABG: any prior occurrence
PTCA or other procedure: 1-60 months prior to screening

Amended--
OLD >
(5.2.1 Inclusion Criteria)

- Lipid-regulating drugs not specified as study treatment in the protocol: probucol, fibrates and derivatives, bile-acid sequestering resins, other HMG-CoA reductase inhibitors, niacin (>500 mg/day), and supplemental fish oils. HMG-CoA reductase inhibitors may only be taken as described in the protocol.

<u>January 15, 1999 - Amendment 2</u>
NEW>
(5.2.1 Inclusion Criteria)

- Lipid-regulating drugs not specified as study treatment in the protocol: probucol, fibrates and derivatives, bile-acid sequestering resins, other HMG-CoA reductase inhibitors, niacin (>500 mg/day), and supplemental fish oils ***prescription only***. HMG-CoA reductase inhibitors may only be taken as described in the protocol.

Amended--
OLD >
(6.4.1 Laboratory Evaluation)

Following randomization, lipid and lipoprotein determinations will not be reported to study sites except as follows: if a patient's LDL-C is maintained at ≥130 mg/dl (3.4 mmol/l) or their triglycerides increase to > 1,000 mg/dl (11.3 mmol/l) at the Visit 9 (month 12) determination or as measured annually thereafter, the central laboratory will notify the sponsor and the study site. In this situation, the patient's glycemic control and/or dietary compliance should be reviewed, other hygienic measures instituted, and a second lipid measurement performed approximately 4 weeks later. If the second value is still greater than the above guidelines, the sponsor and study site will be notified by the central laboratory, and add-on drug therapy may be instituted at the discretion of the investigator

and approval of the Parke-Davis or Pfizer medical monitor or their designee. A lipid measurement must be performed 4 weeks after add-on therapy is instituted. If the value is still above the guidelines, consideration should be given to discontinuing study medication, but patient follow-up must continue as described in section 6.6.

January 15, 1999 - Amendment 2
NEW>
(6.4.1 Laboratory Evaluation)

Following randomization, lipid and lipoprotein determinations ***(including those at Visit 5)*** will not be reported to study sites except as follows: if a patient's LDL-C is maintained at ≥130 mg/dl (3.4 mmol/l) or their triglycerides increase to > 1,000 mg/dl (11.3 mmol/l) at the Visit 9 (month 12) determination or as measured annually thereafter, the central laboratory will notify the sponsor and the study site. In this situation, the patient's glycemic control and/or dietary compliance should be reviewed, other hygienic measures instituted, and a second lipid measurement performed approximately 4 weeks later. If the second value is still greater than the above guidelines, the sponsor and study site will be notified by the central laboratory, and add-on drug therapy may be instituted at the discretion of the investigator and approval of the Parke-Davis or Pfizer medical monitor or their designee. A lipid measurement must be performed 4 weeks after add-on therapy is instituted. If the value is still above the guidelines, consideration should be given to discontinuing study medication, but patient follow-up must continue as described in section 6.6.

Amended---
OLD >

6.4.2. Adverse Event Reporting [c]

Each patient will be observed and queried in a nonspecific fashion at each visit after screening (Visit 1) during the study for any new or continuing symptoms since the previous visit. All serious and nonserious adverse events must be recorded on the appropriate CRF including the onset, duration, severity, relationship to study drug and ultimate management as detailed in Appendix D.

Clinical end points and associated symptoms as defined in Section 6.3, are considered efficacy parameters and are not to be reported as adverse events. Procedural-related complications that do not meet the definition of an the clinical end points as defined in Sections 6.3 will be considered an adverse event. For

example, pneumonia during the hospital stay for a CABG will be considered an adverse event, while a myocardial infarction resulting from a CABG will be considered a clinical end point.

January 15, 1999 - Amendment 2
NEW>

6.4.2. Adverse Event Reporting [c]

(c) See Appendix D, Administrative Procedures For The Reporting Of Adverse Events

Each patient will be observed and queried in a nonspecific fashion at each visit after screening (Visit 1) during the study for any new or continuing symptoms since the previous visit. All serious and nonserious adverse events must be recorded on the appropriate CRF including the onset, duration, severity, relationship to study drug and ultimate management as detailed in Appendix D.

Rechallenges for medically significant adverse events that are considered to be caused by atorvastatin should follow the procedures outlined in Section 6.4.1.1.

Clinical end points and associated symptoms as defined in Section 6.3, are considered efficacy parameters and are not to be reported as adverse events. Procedural-related complications that do not meet the definition of an the clinical end points as defined in Sections 6.3 will be considered an adverse event. For example, pneumonia during the hospital stay for a CABG will be considered an adverse event, while a myocardial infarction resulting from a CABG will be considered a clinical end point.

Amended---

OLD >

(Footnotes to Appendix A.1)

[a] height (screen only), weight, blood pressure
[b] serum chemistry and hematology by central laboratory and uninalysis at local site (see Appendix C.4.)
[c] includes TSH, *pregnancy test (women of child-bearing potential), and* HbA1C at screening; *pregnancy testing (women of child-bearing potential)* and the HbA1C annually (known/suspected diabetics only) beginning at Visit 9
[d] includes total cholesterol, triglycerides, LDL-C, HDL-C
[e] includes apolipoprotein A1, B
[f] includes transaminases (ALT, AST) and CPK if clinically indicated
[g] can be done between Visit 4 or 5 once a patient's lipid levels qualify them for randomization
[h] open label atorvastatin 10 mg
[i] the semi-annual visit schedule beginning with Visits 10 and 11 will extend beyond month 72 for patients beyond this time point if the trial has not yet been stopped
j.includes resource utilization for economic analyses

<u>January 15, 1999 - Amendment 2</u>

NEW>

(Footnotes to Appendix A.1)

[a]height (screen only), weight, blood pressure
[b]serum chemistry and hematology by central laboratory and uninalysis at local site (see Appendix C.4.)
[c] includes TSH, *pregnancy test (women of child-bearing potential), and* HbA1C at screening; *pregnancy testing (women of child-bearing potential)* and the HbA1C annually (known/suspected diabetics only) beginning at Visit 9; *No Pregnancy Test or HbA1C will be performed at Visit 5. Additional plasma will be stored at Visit 5, 9, and annually thereafter.*
[d] includes total cholesterol, triglycerides, LDL-C, HDL-C
[e] includes apolipoprotein A1, B
[f] includes transaminases (ALT, AST) and CPK if clinically indicated
[g] can be done between Visit 4 or 5 once a patient's lipid levels qualify them for randomization
[h] open label atorvastatin 10 mg
[i] the semi-annual visit schedule beginning with Visits 10 and 11 will extend beyond month 72 for patients beyond this time point if the trial has not yet been stopped
[j] includes resource utilization for economic analyses

(c) See Appendix D, Administrative Procedures For The Reporting Of Adverse Events

Amended---
OLD >

24. Tonken A. LIPID Study. Preliminary results presented at the 70th Scientific Sessions of the American Heart Association. Orlando, Florida; November 1997.

January 15, 1999 - Amendment 2
NEW>

24. The Long-Term Intervention with Pravastatin in Ischemic Disease (LIPID) Study Group. Prevention of cardiovascular events and death with pravastatin in patients with coronary heart disease and a broad range of initial cholesterol levels. N Engl J Med 1998; 339:1349-57.

Amended---
OLD >

APPENDIX A.5
OBJECTIVE CRITERIA FOR ATHEROSCLEROTIC CHD

Patient must have been hospitalized with a discharge diagnosis that includes angina, chest pain, or similar condition, and has documented evidence of at least one of the following:

1. chest pain with associated evidence of myocardial ischemia defined as either
   - electrocardiographic evidence
   - any positive myocardial stress test (e.g., thallium^{201})
   - any wall motion abnormality as detected on cardiac ultrasound

   October 6, 1998 - Amendment 1
   - *new coronary angiographic lesions of ≥ 50% stenosis in one or more epicardial vessels.*

2. use of thrombolytic therapy with associated reduction of chest pain symptoms (i.e., aborted myocardial infarction)

January 15, 1999 - Amendment 2
NEW>
Appendix A-5 - Objective Criteria for Atheroschlerotic CHD <Appendix deleted>

Amended---
OLD >
(Appendix B - Commonly Prescribed Prohibited Medication)
The following are also prohibited:
- any chronic systemic anti-fungal agent use
- mibefradil dihydrochloride (Posicor®)
- fish oils

Continued OLD
October 6, 1998 - Amendment 1
- *nefazodone*
- *fluvoxamine*
- *concurrent use of other potent cytochrome P450-3A4 inhibitors*

<u>January 15, 1999 - Amendment 2</u>
NEW>
(Appendix B - Commonly Prescribed Prohibited Medication)
The following are also prohibited:

- any chronic systemic anti-fungal agent use
- mibefradil dihydrochloride (Posicor®)
- fish oils ***(prescription only)***

<u>October 6, 1998 - Amendment 1</u>

- *nefazodone*
- *fluvoxamine*
- *concurrent use of other potent cytochrome P450-3A4 inhibitors*

Amended---
OLD>

APPENDIX C.2
ELECTROCARDIOGRAM

<u>October 6, 1998 - Amendment 1</u>
A 12-lead electrocardiogram will be recorded as scheduled with the patient recumbent and resting. The electrocardiogram *should be performed and submitted to* [redacted] *using the procedure specified for routine ECGs.*
<u>October 6, 1998 - Amendment 1</u>
If a patient experiences a myocardial infarction, an ECG should be performed between 6 weeks and 3 months following the myocardial infarction. It may be done at a regularly scheduled visit if the visit is to occur within that time frame. The ECG should be performed and submitted to [redacted] *using the procedure specified for routine ECGs.*

<u>January 15, 1999 - Amendment 2</u>
NEW>

APPENDIX C.2
ELECTROCARDIOGRAM

A 12-lead electrocardiogram will be recorded as scheduled with the patient recumbent and resting. The electrocardiogram *should be performed and submitted to* [redacted]*e* [redacted]*s* [redacted]*) using the procedure specified for routine ECGs.*

If a patient experiences a myocardial infarction ***after randomization, a new baseline*** *ECG, an ECG should be performed between 6 weeks and 3 months following the myocardial infarction. It may be done at a regularly scheduled visit if the visit is to occur within that time frame.* ***If not, a new baseline ECG should be performed at an unscheduled study visit.*** *The ECG should be performed and submitted to CCD using the procedure specified for routine ECGs.*

Appendix I

Amendment 3 – June 4, 1999

APPENDIX I

Amendment 3 – June 4, 1999

Amended---
OLD>
(Table of Contents)

11. REFERNCES
12. LIST OF APPENDICES

<u>**June 4, 1999 – Amendment**</u>
NEW>

11. DNA Databank ***27***

11.1 Introduction..

11.2 Procedure...

11.3 Process for Anonymity of Blood Samples ....

12. REFERENCES...

13. LIST OF APPENDICIES

A. Timetable of Visits and Procedures 32

B. Commonly Prescribed Prohibited Medications... 40

C. Study Safety Procedures and Clinical Laboratory Determinations...................... 43

<u>October 6, 1998 - Amendment 1</u>

D. Administrative Procedures and Clinical Laboratory Determinations ....D-i

E. Other Administrative and Regulatory Procedures.. E-i

F. Declaration of Helsinki ....F-i

G. Amendments - October 6, 1998..*G-1*

<u>January 15, 1999 - Amendment 2</u>

H. Amendments - January 15, 1999 ....*H-1*

<u>**June 4, 1999 – Amendment 3**</u>

I. Amendments – June 4, 1999 I-1

Amended---
OLD>

(5.3. Prohibited Medications or Precautions)

<u>October 6, 1998 - Amendment 1</u>

- Other drugs known to be associated with an increased risk of rhabdomyolysis in combination with HMG-CoA reductase inhibitors [*including:* erythromycin, *any chronic systemic antifungal use*, mibefradil dihydrochloride, *nefazodone, fluvoxamine and other potent inhibitors of cytochrome P450-3A4*].

NEW>
June 4, 1999 – Amendment 3

- Other drugs known to be associated with an increased risk of rhabdomyolysis in combination with HMG-CoA reductase inhibitors [*including:* erythromycin, ***clarithromycin***, *any chronic systemic antifungal use*, mibefradil dihydrochloride, *nefazodone, fluvoxamine and other potent inhibitors of cytochrome P450-3A4*]. ***Coadministration of atorvastatin (10 mg) and azithromycin (500 mg QD) has been shown not to alter plasma concentrations of atorvastatin.***

Amended---
OLD>
(6.4.1. Laboratory Evaluation)

January 15, 1999 - Amendment 2
Following randomization, lipid and lipoprotein determinations *(including those at Visit 5)* will not be reported to study sites except as follows: if a patient's LDL-C is maintained at ≥130 mg/dl (3.4 mmol/l) or their triglycerides increase to > 1,000 mg/dl (11.3 mmol/l) at the Visit 9 (month 12) determination or as measured annually thereafter, the central laboratory will notify the sponsor and the study site. In this situation, the patient's glycemic control and/or dietary compliance should be reviewed, other hygienic measures instituted, and a second lipid measurement performed approximately 4 weeks later. If the second value is still greater than the above guidelines, the sponsor and study site will be notified by the central laboratory, and add-on drug therapy may be instituted at the discretion of the investigator and approval of the Parke-Davis or Pfizer medical monitor or their designee. A lipid measurement must be performed 4 weeks after add-on therapy is instituted. If the value is still above the guidelines, consideration should be given to discontinuing study medication, but patient follow-up must continue as described in section 6.6.

If a patient's LDL-C is less than 50 mg/dl (<1.3 mmol/l), the central laboratory will notify the sponsor and study site.

June 4, 1999 – Amendment 3
NEW>
(6.4.1 Laboratory Evaluation)

Following randomization, lipid and lipoprotein determinations (including those at Visit 5) will not be reported to study sites. The central lab will monitor LDL values, starting at Visit 9 and annually thereafter, and triglyceride values, starting at Visit 6 and annually thereafter and if necessary action will be taken according to the following plan:

LDL Action Plan. If the LDL level at a scheduled annual visit (visits 9, 11, 13, 15, 17...) is either ≥130 mg/dl (3.4 mmol/l) or <50 mg/dl (<1.3 mmol/l), the central laboratory will notify the study site that the level is "outside protocol limits"(the specific LDL value will remain blinded). The site will have the patient return for a repeat fasting lipid panel and TSH determination within 3-4 weeks. During the period prior to retest, the site will be contacted by the Parke-Davis or Pfizer medical monitor or their designee to reinforce compliance with study medication and non-pharmacological hygienic measures. Actions based on retest result levels are as follows:

- **25-130 mg/dl (~0.7-3.4 mmol/l). LDL levels and treatment group will continue to be blinded. Study medication should continue and no further action is required for this patient group.**
- **<25 mg/dl (<0.7mmol/l). LDL levels will continue to be blinded but treatment group will be unblinded. It is recommended that study medication continue however, in discussion with the Parke-Davis or Pfizer medical monitor or their designee, various alternatives for continued treatment will be discussed and the site will document a treatment plan. The preferred treatment action for this patient group is to remain on study medication.**
- **131-160 mg/dl (~3.4-4.1 mmol/l). LDL levels and treatment group will continue to be blinded. Study medication should be continued. Retests are to be done at the next scheduled visit and sites will be requested to continue to reinforce compliance with study medication and non-pharmacological hygienic measures. If at semi-annual retest, the LDL continues in the 131-160 range, repeat determination will be managed as described below in the >160 mg/dl group.**
- **>160 mg/dl (>4.1 mmol/l). LDL levels and treatment group will continue to be blinded. Study medication should be continued. In discussion with the Parke-Davis or Pfizer medical monitor or their designee, various alternatives for add-on therapy will be discussed and the site will be required to document a follow-up and treatment plan.**

Triglyceride Action Plan. If the triglyceride level at any post-randomization scheduled visit is ≥1,000 mg/dl (11.3 mmol/l), the central laboratory will notify the study site that the level is in the critical range and the value will be reported. The site will be requested to have the patient return for a repeat determination within 2 weeks. During the period prior to retest, the site will be contacted by the Parke-Davis or Pfizer medical monitor or their designee to reinforce compliance with study medication , low carbohydrate diet and restriction of alcohol intake. Actions based on retest result levels are as follows:

- **<1,000 mg/dl (11.3 mmol/l). Triglyceride levels and treatment group will be blinded and study medication should be continued. No further action is required for this patient group.**
- **≥1,000 mg/dl (11.3 mmol/l). Triglyceride levels and treatment group will be unblinded. In discussion with the Parke-Davis or Pfizer medical monitor or their designee, various alternatives for continued treatment will be discussed including add-on therapy. The site will be required to document a follow-up and treatment plan.**

Amended--

OLD>

<u>October 6, 1998 - Amendment 1</u>

Following normalization of a clinically important laboratory abnormality, the patient may be rechallenged with study medication. Rechallenges must be discussed with, and approved by, the Parke-Davis or Pfizer clinical monitor (or their designee) and appropriate IRB/EC. These patients will maintain their original treatment group assignment during rechallenge. *A rechallenge is defined as reinstitution of study drug following discontinuation for a medically significant adverse event considered to be caused by atorvastatin.*

NEW>

June 4, 1999 – Amendment 3

Following normalization of a clinically important laboratory abnormality ***considered causally related to atorvastatin***, the patient may be rechallenged with study medication. Rechallenges must be discussed with, and approved by, the Parke-Davis or Pfizer clinical monitor (or their designee) and appropriate IRB/EC. These patients will maintain their original treatment group assignment during rechallenge. *A rechallenge is defined as reinstitution of study drug following discontinuation for a medically significant adverse event considered to be caused by atorvastatin.* ***(See Section 6.4.2.3 for further details)***

Amended---

OLD>

January 15, 1999 - Amendment 2

Rechallenges for medically significant adverse events that are considered to be caused by atorvastatin should follow the procedures outlined in Section 6.4.1.1.

NEW>

June 4, 1999 – Amendment 3

Rechallenges for medically significant adverse events that are considered to be caused by atorvastatin should follow the procedures outlined in ***Section 6.4.2.3.***

Amended---

(6.4.2.1. Serious Adverse Events[(d)])

June 4, 1999 – Amendment 3

NEW>

Hospitalizations for diagnostic procedures performed for underlying coronary disease that are not in association with acute medical events shall not be reported as SAEs provided: 1) The procedure was ordered in routine management of the underlying disease state for which the patient is being treated in the study. 2) The patient was not hospitalized for a worsening of the underlying disease state. 3) The patient was not hospitalized for an acute event which subsequently led to a procedure.

Overdoses with an associated adverse event are to be reported as serious adverse events. Overdoses are defined as follows:

- ***Pre-Randomization: greater than 8 tablets of open label drug taken in a 24 hour period.***
- ***Post Randomization: greater than 8 tablets from bottle A, or greater than 2 tablets from bottle B, taken in a 24 hour period.***

Amended---

NEW>

June 4, 1999 – Amendment 3

6.4.2.3 Rechallenge Procedure

If after normalization of a study medication related adverse event or significant lab abnormality, rechallenge with study medication is to follow the procedure described below. The rechallenge must be approved by the appropriate sponsor medical personnel or their designees. The rechallange process as well as the risks and benefits

---

[(d)] See Appendix D, Administrative Procedures for the Reporting of Adverse Events (Section 4.1, Immediately Reportable Adverse Events)

of rechallenge should be discussed with the patient. If the physician and patient agree, the patient will be asked to sign the appropriate, IRB/EC approved (and sponsor approved if applicable) consent form addendum for the rechallenge. One of two options for restarting study medication is to followed:

1. *Patient will be re-started at full dose of study medication (1-A and 2-B tablets). For laboratory abnormalities, patients will have follow-up labs at 6,12,18, and 24 weeks post-start or at a frequency agreed to between the sponsor medical team and investigator. For non-laboratory abnormality related rechallenges, follow-up visits should be as agreed between the investigator and sponsor medical team or their designee(s). If there is a recurrence of the adverse event/lab abnormality, continued study medication dosing and follow-up should be determined (by the investigator) in conjunction with the sponsor medical team or their designee(s). Patients should resume the normal study visit schedule following the above specified monitoring period.*
2. *Patient will re-start study medication taking open-label 10 mg atorvastatin. For laboratory abnormality related rechallenges, follow-up labs will be done at 6 weeks post-restart. If all parameters are at satisfactory levels, the patient will stop open-label 10 mg atorvastatin and begin taking 1 tablet from bottle A and 1 tablet from bottle B. Follow-up labs will again be performed at 6 weeks following increasing the dose. If all laboratory parameters are still at satisfactory levels, the patient will be instructed to increase the dose to 1 tablet from bottle A and 2 tablets from bottle B. Repeat labs will once again be performed 6 and 12 weeks after increasing the dose. For non-laboratory abnormality related rechallenges, follow-up visits should be as agreed between the investigator and the sponsor medical team or their designee(s). The patient will resume a normal study visit schedule after the above specified monitoring period is completed. If repeat labs show elevations, or if there is recurrence of the clinical adverse event, continued study medication dosing and follow-up should be determined (by investigator) in conjunction with the sponsor medical team or their designee(s).*

Deviations from the procedure outlined above will only be allowed after discussion and approval with the sponsor medical team or their designee(s) and, if necessary, approval of the site's IRB/EC. The Individual patient rechallenge plan will be documented (by the site) in the patient's medical record.

Amended--

OLD> **11. REFERENCES**

June 4, 1999 – Amendment 3

NEW> 11. DNA Databank

11.1 INTRODUCTION

New developments in molecular biology have directed research of human disease towards its genetic basis. The understanding of the molecular origin of a disorder and the insight into the way genetic information is expressed or modulated by the environment will have a major impact on treatment and prevention of disease. It is estimated that the genetic predisposition for atherosclerotic disease involves more than 200 genes. The exact role in the atherosclerotic process is only known for a small number of genes. These genes play an important role in the aetiology of atherosclerotic disorders such as coronary heart disease (CHD), as has been shown in a large number of studies. The use of large, well documented patient cohorts has enabled the identification of new genetic risk factors for CHD. The aim of the DNA databank is to establish the relationship between a number of genetic markers, known to influence cardiovascular risk in the general population, and the clinical phenotype and response to lipid lowering therapy in patients with pre-existing cardiovascular disease.

11.2. PROCEDURE

At Visit 5 or the first possible visit at which blood is collected post-randomization, 12 mls of whole blood will be collected into plastic tubes containgin EDTA from randomized subjects who consent to undergo additional genetic testing. Refusal to participate in the additional testing will not effect the subject's participation in the main study. After collection and labeling of the sample, the whole blood sample with the other biochemical samples collected during this visit will be mailed to:

[redacted] *(US Sites)* *or* [redacted]

11.3. PROCESS FOR ANONYMITY OF BLOOD SAMPLES

Prior to transfer to the genetics laboratory that will perform the DNA analyses, the blood sample will be relabeled with an anonymous bar code label which contains no patient identifying information. A sample registration form which contains an identical bar code will be used to record the patient entry/screening number, patient randomization number, and date of collection. The registration form is mailed separately to an administrator and held secured until the patient data is transferred to the disease database as described below.

Demographic and disease-related characteristics of subjects in this study who agree to participate in genetic testing will be extracted from the database and moved to a separate Disease Database. This process will not alter the protocol-specific database in any way. For data copied into the Disease Database, the subject identifying information will be removed and the demographic, disease information, and their lipid profile will only be linked to samples collected. Results of such tests will be kept confidential and will not be available to investigators or subjects. Samples and disease information will be used to help define disease characteristics that are associated with specific genetic and biochemical markers. When the study is completed, the secure administrator will populate the Disease Database with the genotype data, biochemical

data, disease information, but no patient identifiers will be transferred. The genetic sample registration form will then be destroyed.

Amended---
OLD>
11. REFERENCES

June 4, 1999 – Amendment 3
NEW>
12. REFERENCES

Amended---
OLD>
12. LIST OF APPENDICES

June 4, 1999 – Amendment 3
NEW>
13. LIST OF APPENDICES

**Appendix**	**Title**
A	Schedule and Description of Study Procedures
B	Commonly Prescribed Prohibited Medications
C	Study Safety Procedures and Clinical Laboratory Determinations
D	Administrative Procedures for the Reporting of Adverse Events
E	Other Administrative and Regulatory Procedures
F	Declaration of Helsinki
G	*Amendments - October 6, 1998*
H	*Amendments – January 15, 1999*
I	***Amendments – June 4, 1999***

Amended---
OLD>
<u>October 6, 1998 - Amendment 1</u>

APPENDIX A. 1
TIMETABLE OF CLINIC VISITS AND PROCEDURES

Study Period	Screening	Atorvastatin 10 mg *<letter/sentence/ paragraph deleted>*			Double-Blind Treatment *<letter/sentence/paragraph deleted>*						
**Visit #**	**1**	**2**	**3**	**4**	**5**	**6**	**7**	**8**	**9**	**10,12,14,16,18[i]**	**11,13,15,17,19[i]**
**Study week**		**wk -8**	**wk -4**	**wk -2**	**wk 0**	**wk 12**					
**Study month**							**mo 6**	**mo 9**	**mo 12**	**mos. 18, 30, 42,54, 66**	**mos. 24, 36, 48, 60, 72**
**PROCEDURE**											
**Informed Consent**	X										
**Demographics/ Med. History**	X										
**Vital Signs [a]**	X	X	X	X	X	X	X	X	X	X	X
**Clinical Labs [b,c,]**	X				X				X		X
**Lipid Labs [d]**	X	X	X	X	X[e]	X			X[e]		X
**Safety Labs [f]**				X		X					
**Dietary Counseling**	X----	------------------	------------------	------------------	------------------	------throughout trial	------------------	------------------	------------------	------------------	----------X
**Physical Exam**				X[g]----	----X[g]				X		X
**ECG**				X[g]----	----X[g]				X		X
**Dispense Drug**		X[h]			X	X	X	X	X	X	X
**Concom. Therapy**	X	X	X	X	X	X	X	X	X	X	X
**Adverse Events**		X	X	X	X	X	X	X	X	X	X
**End Points[j]**		X	X	X	X	X	X	X	X	X	X

[a] height (screen only), weight, blood pressure
[b] serum chemistry and hematology by central laboratory and uninalysis at local site (see Appendix C.4.)
<u>January 15, 1999 - Amendment 2</u>
[c] includes TSH, *pregnancy test (women of child-bearing potential), and* HbA1C at screening*; pregnancy testing (women of child-bearing potential)* and the HbA1C annually (known/suspected diabetics only) beginning at Visit 9; *No Pregnancy Test or HbA1C will be performed at Visit 5. Additional plasma will be stored at Visits 5, 9, and annually thereafter.*
[d] includes total cholesterol, triglycerides, LDL-C, HDL-C
[e] includes apolipoprotein A1, B
[f] includes transaminases (ALT, AST) and CPK if clinically indicated
[g] can be done between Visit 4 or 5 once a patient's lipid levels qualify them for randomization
[h] open label atorvastatin 10 mg
[i] the semi-annual visit schedule beginning with Visits 10 and 11 will extend beyond month 72 for patients beyond this time point if the trial has not yet been stopped
[j] includes resource utilization for economic analyses

June 4, 1999 – Amendment 3
NEW>

APPENDIX A. 1
TIMETABLE OF CLINIC VISITS AND PROCEDURES

Study Period	Screening	Atorvastatin 10 mg *<letter/sentence/ paragraph deleted>*			Double-Blind Treatment *<letter/sentence/paragraph deleted>*						
**Visit #**	**1**	**2**	**3**	**4**	**5**	**6**	**7**	**8**	**9**	**10,12,14,16,18[i]**	**11,13,15,17,19[i]**
**study week**		**wk -8**	**wk -4**	**wk -2**	**wk 0**	**wk 12**					
**study month**							**mo 6**	**mo 9**	**mo 12**	**mos. 18, 30, 42,54, 66**	**mos. 24, 36, 48, 60, 72**
**PROCEDURE**											
**Informed Consent**	X										
**Demographics/ Med. History**	X										
**Vital Signs[a]**	X	X	X	X	X	X	X	X	X	X	X
Clinical Labs [b,c,k]	X				X				X		X
Lipid Labs [d,k]	X	X	X	X	X[e]	X			X[e]		X
Safety Labs [f,k]				X		X					
**Dietary Counseling**	X----	---------------------throughout trial---------------------									----------X
**Physical Exam**				X[g]----	----X[g]				X		X
**ECG**				X[g]----	----X[g]				X		X
**Dispense Drug**		X[h]			X	X	X	X	X	X	X
**Concom. Therapy**	X	X	X	X	X	X	X	X	X	X	X
**Adverse Events**		X	X	X	X	X	X	X	X	X	X
**End Points[j]**		X	X	X	X	X	X	X	X	X	X

[a] height (screen only), weight, blood pressure
[b] serum chemistry and hematology by central laboratory and uninalysis at local site (see Appendix C.4.)
<u>January 15, 1999 - Amendment 2</u>
[c] includes TSH, *pregnancy test (women of child-bearing potential), and* HbA1C at screening*; pregnancy testing (women of child-bearing potential)* and the HbA1C annually (known/suspected diabetics only) beginning at Visit 9; *No Pregnancy Test or HbA1C will be performed at Visit 5. Additional plasma will be stored at Visits 5, 9, and annually thereafter.*
[d] includes total cholesterol, triglycerides, LDL-C, HDL-C
[e] includes apolipoprotein A1, B
[f] includes transaminases (ALT, AST) and CPK if clinically indicated
[g] can be done between Visit 4 or 5 once a patient's lipid levels qualify them for randomization
[h] open label atorvastatin 10 mg
[i] the semi-annual visit schedule beginning with Visits 10 and 11 will extend beyond month 72 for patients beyond this time point if the trial has not yet been stopped
[j] includes resource utilization for economic analyses
k. one 12 mls whole blood will be collected (at Visit 5 or the first available post-randomization visit) for the DNA database

Amended---
OLD >

Visit 5

- Assign patient number
- Draw blood samples for a complete clinical laboratory, lipid profile, special lipids, hematology and plasma storage
- Vital signs
- Urinalysis
- Physical Examination (if not done at Visit 4)
- Perform baseline ECG (if not done at Visit 4)
- Continue dietary counseling
- Dispense study medication
- Record adverse events
- Record potential end points
- Record changes in and additions to concurrent medications

June 4, 1999 – Amendment 3
NEW >

Visit 5

- Assign patient number
- Draw blood, ***DNA databank (Note: this sample will be collected at this visit or the first visit after Visit 5 where blood samples are drawn)*** samples for a complete clinical laboratory, lipid profile, special lipids, hematology and plasma storage
- Vital signs
- Urinalysis
- Physical Examination (if not done at Visit 4)
- Perform baseline ECG (if not done at Visit 4)
- Continue dietary counseling
- Dispense study medication
- Record adverse events
- Record potential end points
- Record changes in and additions to concurrent medications

Amended---
OLD>
(Appendix C.4)
October 6, 1999 – Amendment 1
TSH (at Visit 1 only)

NEW>
June 4, 1999 Amendment 3
TSH (at Visit 1 and as described on the LDL action plan)

Appendix J

Amendment 4 – June 13, 2003

APPENDIX J

Amendment 4 – June 13, 2003

Amended---

June 13, 2003 – Amendment 4

Parke-Davis Pharmaceutical Research has been removed from the protocol in the following sections:

All header information, cover page, ii, iii, iv, v, vi, pg 5 (section 4), pg 12 (section 6.2.3 and 6.3.4), pg 14 (section 6.3.4), pg 15 (section 6.4.10), pg 16 (section 6.4.1 and 6.4.1.1), pg 17 (section 6.4.1.1), pg 18 (section 6.4.2.1), pg 19 (section 6.4.2.1), pg 22 (section 7.1), pg 23 (section 8),

Amended---

OLD>

PFIZER PROTOCOL #981-117/SYNOPSIS

NEW>

June 13, 2003 – Amendment 4

PFIZER PROTOCOL #981-117/*258-102* SYNOPSIS

Amended---

OLD>

DESCRIPTION OF MEDICATIONS

Parke-Davis CI Numbers	Pfizer Drug Code	Generic Name	Strength and Dosage Form	Therapeutic Classification
981	258	atorvastatin	40-mg tablets	lipid regulator
981	258	atorvastatin	10-mg tablets	lipid regulator

NEW>

June 13, 2003 – Amendment 4

DESCRIPTION OF MEDICATIONS

Pfizer Drug Code	Generic Name	Strength and Dosage Form	Therapeutic Classification
981/258	atorvastatin	40-mg tablets	lipid regulator
981/258	atorvastatin	10-mg tablets	lipid regulator

Amended---
OLD>

PROTOCOL FOR PARKE-DAVIS/PFIZER PHARMACEUTICAL RESEARCH
CLINICAL TRIAL

TITLE: The Effect Of LDL-Cholesterol Lowering Beyond Currently Recommended Minimum Targets On Coronary Heart Disease (CHD) Recurrence In Patients With Pre-Existing CHD

Parke-Davis Clinical/Medical Colleagues:

Name: [redacted]e[redacted] MD ______________________ __________
Title: Sr. Director, Medical Research Signature Date
Department: Medical Research

Name: [redacted]a[redacted]r, PhD ______________________ __________
Title: Sr. Director, Biometrics, Epidemiology and Data Management Signature Date
Department: Medical Research

Pfizer Clinical/Medical Colleagues:

Name: [redacted].[redacted], Dr PH ______________________ __________
Title: Director, Metabolic Diseases Signature Date
Department: Central Research

Name: [redacted].[redacted], PhD ______________________ __________
Title: Assoc. Director II, Biometrics Signature Date
Department: Central Research

Parke-Davis Pharmaceutical Research
Division of Warner-Lambert Company
International Clinical Development

Name: [redacted]h[redacted]s ______________________ __________
Title: International Study Manager Signature Date
Address: Lambert Court, Chestnut Ave.
Eastleigh, SO53 3ZQ
UK

The above signed confirms herewith to have read and understood this trial protocol and attached appendices, furthermore, to accomplish this study according to the protocol and the Good Clinical Practice guidelines, as well as local regulations, and to accept respective revisions conducted by authorized personnel of Parke-Davis Pharmaceutical Research or Pfizer and by competent authorities.

PROTOCOL FOR PARKE-DAVIS/PFIZER PHARMACEUTICAL RESEARCH
CLINICAL TRIAL

TITLE: The Effect Of LDL-Cholesterol Lowering Beyond Currently Recommended Minimum Targets On Coronary Heart Disease (CHD) Recurrence In Patients With Pre-Existing CHD

Principal Investigator:
Name: ______________________ __________
Address: Signature Date

Co-Principal Investigator:
Name: ______________________ __________
Address: Signature Date

The above signed confirms herewith to have read and understood this trial protocol and attached appendices, furthermore, to accomplish this study according to the protocol and the Good Clinical Practice guidelines, as well as local regulations, and to accept respective revisions conducted by authorized personnel or Pfizer and by competent authorities.

NEW>

June 13, 2003 – Amendment 4

PROTOCOL FOR ***<letter/sentence/paragraph deleted>*** PFIZER ***<letter/sentence/paragraph deleted>***

TITLE: The Effect Of LDL-Cholesterol Lowering Beyond Currently Recommended Minimum Targets On Coronary Heart Disease (CHD) Recurrence In Patients With Pre-Existing CHD

<letter/sentence/paragraph deleted>

<letter/sentence/paragraph deleted>

Pfizer Clinical/Medical Colleagues:

Name:	[redacted].[redacted], Dr PH	______________________	__________
Title:	***Executive*** Director, Metabolic Diseases	Signature	Date
Department:	***Pfizer Global Research and Development***		
Name:	[redacted]	______________________	__________
Title:	***Mecical Director***	Signature	Date
Department:	***Pfizer Global Pharmaceuticals***		
Name:	[redacted]	______________________	__________
Title:	***Senior Director Biometrics***	Signature	Date
Department:	***Pfizer Global Pharmaceuticals***		

The above signed confirms herewith to have read and understood this trial protocol and attached appendices, furthermore, to accomplish this study according to the protocol and the Good Clinical Practice guidelines, as well as local regulations, and to accept respective revisions conducted by authorized personnel *<letter/sentence/paragraph deleted>* of Pfizer and by competent authorities.

PROTOCOL FOR ***<letter/sentence/paragraph deleted>*** PFIZER ***<letter/sentence/paragraph deleted>*** CLINICAL TRIAL

TITLE: The Effect Of LDL-Cholesterol Lowering Beyond Currently Recommended Minimum Targets On Coronary Heart Disease (CHD) Recurrence In Patients With Pre-Existing CHD

Principal Investigator:<br>Name:<br>Address:	______________________<br>Signature	________<br>Date
Co-Principal Investigator:<br>Name:<br>Address:	______________________<br>Signature	________<br>Date

The above signed confirms herewith to have read and understood this trial protocol and attached appendices, furthermore, to accomplish this study according to the protocol and the Good Clinical Practice guidelines, as well as local regulations, and to accept respective revisions conducted by authorized personnel *<letter/sentence/paragraph deleted>* of Pfizer and by competent authorities.

Amended--

OLD>

LABORATORY(IES): [redacted] [redacted]

NEW>

June 13, 2003 – Amendment 4

LABORATORY(IES): [redacted] [redacted]

Amended--

OLD>

OBJECTIVES To assess the CHD event reduction efficacy and safety of low-density lipoprotein cholesterol (LDL-C) lowering to achieve LDL-C targets beyond currently recommended minimums when compared to only achieving the minimum targets.

NEW>

June 13, 2003 – Amendment 4

OBJECTIVES To assess the ***cardiovascular*** event reduction efficacy and safety of low-density lipoprotein cholesterol (LDL-C) lowering to achieve LDL-C targets beyond

currently recommended minimums when compared to only achieving the minimum targets.

Amended---
OLD>

2. STUDY OBJECTIVE

The primary objective of this study is to determine the degree of additional reduction in CHD risk that will accure to patients by lowering their LDL-C beyond the currently accepted minimum target level for patients with pre-existing CHD. Subsidiary objectives include the safety profile of this treatment strategy, its cost-effectiveness, effect on other atherosclerotic-related events and procedures and total mortality.

NEW>
The primary objective of this study is to determine the degree of additional reduction in ***cardiovascular*** risk that will ***accrue*** to patients by lowering their LDL-C beyond the currently accepted minimum target level for patients with pre-existing CHD. Subsidiary objectives include the safety profile of this treatment strategy, its cost-effectiveness, effect on other atherosclerotic-related events and procedures and total mortality.

Amended---
OLD>

3. SUMMARY OF STUDY

This is a double-blind, randomized, parallel-group study of the recurrence of CHD in patients whose LDL-C will be lowered on average to approximately 100 mg/dl (2.6 mmol/l) compared with those whose LDL-C is approximately 25% lower. Patients will have clinically evident CHD. The study has 3 periods: (1) a screening visit to determine a patient's initial eligibility to participate in the study, (2) an open-label atorvastatin 10 mg run-in period whose primary purpose is to achieve an LDL-C <130 mg/dl (3.4 mmol/l) and determine baseline study parameters, and (3) a randomized double-blind treatment period (atorvastatin 10 vs. 80 mg once daily) expected to require an average 5 years duration or until the target number of primary endpoints is achieved, whichever is sooner.

NEW>
<u>June 13, 2003 – Amendment 4</u>

3. SUMMARY OF STUDY

This is a double-blind, randomized, parallel-group study of the ***occurrence*** of ***cardiovascular events*** in patients whose LDL-C will be lowered on average to approximately 100 mg/dl (2.6 mmol/l) compared with those whose LDL-C is approximately 25% lower. Patients will have clinically evident CHD. The study has 3 periods: (1) a screening visit to determine a patient's initial eligibility to participate in the study, (2) an open-label atorvastatin 10 mg run-in period whose primary purpose is to achieve an LDL-C <130 mg/dl (3.4 mmol/l) and determine baseline study parameters, and (3) a randomized double-blind treatment period (atorvastatin 10 vs. 80 mg once daily) expected to require an average ***5.5*** years duration ***<letter, sentence, paragraph deleted>*** until the target number of ***events*** is achieved ***<letter, sentence, paragraph deleted>.***

Amended---
OLD>
PRIMARY EFFICACY PARAMETERS The occurrence of the primary clinical endpoint of major CHD events (defined as either CHD death or non-fatal myocardial infarction).

SECONDARY EFFICACY PARAMETERS The occurrence of the following clinical events: any CHD event (any primary event or CABG, PTCA, other revascularization procedure, or documented angina; cerebrovascular events (fatal and non-fatal stroke or TIA); peripheral vascular disease; hospitalization with primary diagnosis of CHF; any cardiovascular event (any of the above); and all-cause mortality.

NEW>
June 13, 2003 – Amendment 4
PRIMARY EFFICACY PARAMETERS The occurrence of the primary clinical endpoint of major cardiovascular events (defined as ***<letter, sentence, paragraph deleted>*** CHD death,***<letter, sentence, paragraph deleted>*** non-fatal myocardial infarction, ***resuscitated cardiac arrest, or stroke [fatal or non-fatal]).***

SECONDARY EFFICACY PARAMETERS The occurrence of the following clinical events: ***major coronary event (CHD death, non-fatal myocardial infarction or resuscitated cardiac arrest);*** any ***coronary*** event (***major coronary event*** or CABG, PTCA, other revascularization procedure, ***procedure-related myocardial infarction***, or documented angina); ***cerebrovascular event (fatal or non-fatal stroke, TIA)***; peripheral vascular disease; hospitalization with primary diagnosis of CHF; any cardiovascular event (any of the above); and all-cause mortality.

Amended---
OLD>
PROTOCOL FOR PARKE-DAVIS/PFIZER PHARMACEUTICAL RESEARCH CLINICAL TRIAL
NEW>
June 13, 2003 – Amendment 4
PROTOCOL FOR ***<letter, sentence, paragraph deleted>*** PFIZER ***<letter, sentence, paragraph deleted>***CLINICAL TRIAL

Amended---
OLD>
All revisions and/or amendments to the protocol must be approved in advance in writing by the investigator, the Pfizer or Parke-Davis clinical monitor(s) or their designee, and the appropriate Ethical Review Committee. These will be filed with the Food and Drug Administration in the United States by the Regulatory Affairs Department of Parke-Davis or its designee. The investigator is responsible for the retention of the patient log and patient records.

NEW>
<u>June 13, 2003 – Amendment 4</u>
All revisions and/or amendments to the protocol must be approved in advance in writing by the investigator, the Pfizer ***<letter, sentence, paragraph deleted>*** clinical monitor(s) or their designee, and the appropriate Ethical Review Committee. These will be filed with the Food and Drug Administration in the United States by the Regulatory Affairs Department of ***<letter, sentence, paragraph deleted> Pfizer*** or its designee. The investigator is responsible for the retention of the patient log and patient records.

Amended--
OLD>
5.3. Prohibited Medications or Precautions

The following medications are not be taken at any time during this study:

<u>January 15, 1999 - Amendment 2</u>
- Lipid-regulating drugs not specified as study treatment in the protocol: probucol, fibrates and derivatives, bile-acid sequestering resins, other HMG-CoA reductase inhibitors, niacin (>500 mg/day), and supplemental fish oils (prescription only). HMG-CoA reductase inhibitors may only be taken as described in the protocol.

NEW>
<u>June 13, 2003 – Amendment 4</u>
- Lipid-regulating drugs not specified as study treatment in the protocol: probucol, fibrates and derivatives, bile-acid sequestering resins, other HMG-CoA reductase inhibitors, niacin (>500 mg/day), and supplemental fish oils (prescription only). HMG-CoA reductase inhibitors may only be taken as described in the protocol. ***Following the completion of year 1, patients will be allowed to utilize certain lipid-regulating drugs ("add-on therapy") if the patient's LDL levels are found to be higher than protocol range (see section 6.4.1). "Add-on" therapy will be agreed upon by investigator and Pfizer medical personnel or designee.***

Amended--
OLD>
6.1. Study Design
This is a double-blind, randomized, parallel-group study of the recurrence of CHD in patients whose LDL-C will be lowered on average to approximately 100 mg/dl (2.6 mmol/l) compared with those whose LDL-C is approximately 25% lower. Patients will have clinically evident CHD. Following dietary therapy, all patients meeting initial eligibility criteria will begin an open-label atorvastatin 10 mg (once daily) run-in period. Patients whose LDL-C is < 130 mg/dl (< 3.4 mmol/l) at the end of the run-in period (average of Visits 3 and 4) and meet all other inclusion/exclusion criteria will be randomized to double-blind therapy consisting of either atorvastatin 10 mg/day or 80 mg/day. Treatment and follow-up of patients will be continued until either the average

duration of therapy reaches 5 years or the number of primary events reaches 750, whichever occurs sooner. Early stopping rules may be applied based on interim analyses.

NEW>

June 13, 2003 – Amendment 4

6.1. Study Design

This is a double-blind, randomized, parallel-group study of the recurrence of ***cardiovascular events*** in patients whose LDL-C will be lowered on average to approximately 100 mg/dl (2.6 mmol/l) compared with those whose LDL-C is approximately 25% lower. Patients will have clinically evident CHD. Following dietary therapy, all patients meeting initial eligibility criteria will begin an open-label atorvastatin 10 mg (once daily) run-in period. Patients whose LDL-C is < 130 mg/dl (< 3.4 mmol/l) at the end of the run-in period (average of Visits 3 and 4) and meet all other inclusion/exclusion criteria will be randomized to double-blind therapy consisting of either atorvastatin 10 mg/day or 80 mg/day. Treatment and follow-up of patients will be continued until ***<letter, sentence, paragraph deleted>*** the number of ***major coronary events (CHD death, non-fatal myocardial infarction, or resuscitated cardiac arrest)*** reaches 750 ***(approximately 5.5 years average follow-up).*** Early stopping rules may be applied based on interim analyses.

Amended--

OLD>

6.3.1. Primary Efficacy Parameters

The primary efficacy clinical end point is the occurrence of a major coronary event, defined as either:

-CHD death, or
-non-fatal myocardial infarction

NEW>

June 13, 2003 – Amendment 4

6.3.1. Primary Efficacy Parameters

The primary efficacy clinical end point is the occurrence of a major ***cardiovascular*** event, defined as either:

-CHD death, ***<letter, sentence, paragraph deleted>***
-non-fatal myocardial infarction
-Resuscitated cardiac arrest or
-stroke (fatal or non- fatal)

Amended--

OLD>

6.3.2. Secondary Efficacy Parameters

The secondary efficacy clinical end points are the occurrence of the following:

-any coronary event (primary end point or, CABG, PTCA, other revascularization procedure or documented angina)

-cerebrovascular event (fatal or non-fatal stroke, TIA)
-peripheral vascular disease (PVD)
-hospitalization with primary diagnosis of CHF
-any cardiovascular event (any of the above)
-all cause mortality

In addition, the assessment will include as secondary end points the changes from Visit 2 and baseline in the various lipid and lipoprotein parameters included in the study (Appendix A.4) as well as the relationship between the occurrence of the primary and secondary clinical end points defined above with baseline, relative and absolute changes from baseline in these lipid and lipoprotein parameters.

Subgroup analyses will also be performed to investigate variation in the treatment effect of the primary and secondary efficacy end points by the following: age, gender, smoking status, presence or absence of diabetes, TG (< 200, ≥ 200 mg/dl), CHF, other prevalent pre-existing condition categories of the study cohort, and prevalent concomitant medication use including hormone replacement therapy, aspirin, beta-blockers, or calcium channel blockers.

NEW>
June 13, 2003 – Amendment 4

6.3.2. Secondary Efficacy Parameters

The secondary efficacy clinical end points are the occurrence of the following:
-major coronary event (CHD death, non-fatal myocardial infarction, or resuscitated cardiac arrest)
-any coronary event (***major coronary event*** or, CABG, PTCA, other revascularization procedure, ***procedure-related myocardial infarction*** or documented angina)
-cerebrovascular event (fatal or non-fatal stroke, TIA)
-peripheral vascular disease (PVD)
-hospitalization with primary diagnosis of CHF
-any cardiovascular event (any of the above)
-all cause mortality

In order to validly assess the primary and secondary endpoints and the safety profile of the treatment groups, the vital status of each randomized patient must be obtained at the completion of the study.

In addition, the assessment will include as secondary end points the changes from Visit 2 and baseline in the various lipid and lipoprotein parameters included in the study (Appendix A.4) as well as the relationship between the occurrence of the primary and secondary clinical end points defined above with baseline, relative and absolute changes from baseline in these lipid and lipoprotein parameters.

Subgroup analyses will also be performed to investigate variation in the treatment effect of the primary and secondary efficacy end points by the following: age, gender, smoking status, presence or absence of diabetes, ***baseline*** TG (< 200, ≥ 200 mg/dl), ***baseline LDL, baseline HDL,*** CHF, other prevalent pre-existing condition categories of the study

cohort, and prevalent concomitant medication use (***such as*** hormone replacement therapy, aspirin, beta-blockers, ***<letter, sentence, paragraph deleted>*** calcium channel blockers, ***etc.).***

Amended---
OLD>

1.1.1. Laboratory Evaluation

LDL Action Plan. If the LDL level at a scheduled annual visit (visits 9, 11, 13, 15, 17...) is either ≥130 mg/dl (3.4 mmol/l) or <50 mg/dl (<1.3 mmol/l), the central laboratory will notify the study site that the level is "outside protocol limits" (the specific LDL value will remain blinded). The site will have the patient return for a repeat fasting lipid panel and TSH determination within 3-4 weeks. During the period prior to retest, the site will be contacted by the Parke-Davis or Pfizer medical monitor or their designee to reinforce compliance with study medication and non-pharmacological hygienic measures. Actions based on retest result levels are as follows:

- 25-130 mg/dl (~0.7-3.4 mmol/l). LDL levels and treatment group will continue to be blinded. Study medication should continue and no further action is required for this patient group.

- <25 mg/dl (<0.7mmol/l). LDL levels will continue to be blinded but treatment group will be unblinded. It is recommended that study medication continue however, in discussion with the Parke-Davis or Pfizer medical monitor or their designee, various alternatives for continued treatment will be discussed and the site will document a treatment plan. The preferred treatment action for this patient group is to remain on study medication.

- 131-160 mg/dl (~3.4-4.1 mmol/l). LDL levels and treatment group will continue to be blinded. Study medication should be continued. Retests are to be done at the next scheduled visit and sites will be requested to continue to reinforce compliance with study medication and non-pharmacological hygienic measures. If at semi-annual retest, the LDL continues in the 131-160 range, repeat determination will be managed as described below in the >160 mg/dl group.

- >160 mg/dl (>4.1 mmol/l). LDL levels and treatment group will continue to be blinded. Study medication should be continued. In discussion with the Parke-Davis or Pfizer medical monitor or their designee, various alternatives for add-on therapy will be discussed and the site will be required to document a follow-up and treatment plan.

Triglyceride Action Plan. If the triglyceride level at any post-randomization scheduled visit is ≥1,000 mg/dl (11.3 mmol/l), the central laboratory will notify the study site that the level is in the critical range and the value will be reported. The site will be requested to have the patient return for a repeat determination within 2 weeks. During the period prior to retest, the site will be contacted by the Parke-Davis or Pfizer medical monitor or

their designee to reinforce compliance with study medication, low carbohydrate diet and restriction of alcohol intake. Actions based on retest result levels are as follows:

- <1,000 mg/dl (11.3 mmol/l). Triglyceride levels and treatment group will be blinded and study medication should be continued. No further action is required for this patient group.

- ≥1,000 mg/dl (11.3 mmol/l). Triglyceride levels and treatment group will be unblinded. In discussion with the Parke-Davis or Pfizer medical monitor or their designee, various alternatives for continued treatment will be discussed including add-on therapy. The site will be required to document a follow-up and treatment plan.

NEW>

June 13, 2003 – Amendment 4

LDL Action Plan. If the LDL level at a scheduled annual visit (visits 9, 11, 13, 15, 17...) is either ≥130 mg/dl (3.4 mmol/l) or <50 mg/dl (<1.3 mmol/l), the central laboratory will notify the study site that the level is "outside protocol limits" (the specific LDL value will remain blinded). The site will have the patient return for a repeat fasting lipid panel and TSH determination within 3-4 weeks. During the period prior to retest, the site will be contacted by the ***<letter, sentence, paragraph deleted>*** Pfizer medical monitor or their designee to reinforce compliance with study medication and non-pharmacological hygienic measures. Actions based on retest result levels are as follows:

- 25-130 mg/dl (~0.7-3.4 mmol/l). LDL levels and treatment group will continue to be blinded. Study medication should continue and no further action is required for this patient group.

- <25 mg/dl (<0.7mmol/l). LDL levels will continue to be blinded ***<letter, sentence, paragraph deleted>***. It is recommended that study medication continue however, in discussion with the ***<letter, sentence, paragraph deleted>*** Pfizer medical monitor or their designee, various alternatives for continued treatment will be discussed and the site will document a treatment plan. The preferred treatment action for this patient group is to remain on study medication.

- 131-160 mg/dl (~3.4-4.1 mmol/l). LDL levels and treatment group will continue to be blinded. Study medication should be continued. Retests are to be done at the next scheduled visit and sites will be requested to continue to reinforce compliance with study medication and non-pharmacological hygienic measures. If at semi-annual retest, the LDL continues in the 131-160 range, repeat determination will be managed as described below in the >160 mg/dl group.

- >160 mg/dl (>4.1 mmol/l). LDL levels and treatment group will continue to be blinded. Study medication should be continued. In discussion with the

<letter, sentence, paragraph deleted> Pfizer medical monitor or their designee, various alternatives for add-on therapy ***or use of open-label atorvastatin*** will be discussed and the site will be required to document a follow-up and treatment plan.

Triglyceride Action Plan. If the triglyceride level at any post-randomization scheduled visit is ≥*500* mg/dl (***<letter, sentence, paragraph deleted>***mmol/l), the central laboratory will notify the study site that the level is in the critical range and the value will be reported. The site will be requested to have the patient return for a repeat determination within 2 weeks. During the period prior to retest, the site will be contacted by the ***<letter, sentence, paragraph deleted>*** Pfizer medical monitor or their designee to reinforce compliance with study medication, low carbohydrate diet and restriction of alcohol intake. Actions based on retest result levels are as follows:

- <*500* mg/dl (*5.6* mmol/l). Triglyceride levels and treatment group will be blinded and study medication should be continued. No further action is required for this patient group.
- ≥*500* mg/dl (*5.6* mmol/l). Triglyceride levels ***will be unblinded while out of range.*** In discussion with the ***<letter, sentence, paragraph deleted>*** Pfizer medical monitor or their designee, various alternatives for continued treatment will be discussed including add-on therapy. The site will be required to document a follow-up and treatment plan.

Amended--

OLD>

6.4.1.1. Clinically Important Laboratory Abnormalities

If either of the above abnormalities occur, the Parke-Davis or Pfizer study manager or clinical monitor (or their designees) should be contacted immediately.

Following normalization of a clinically important laboratory abnormality considered causally related to atorvastatin, the patient may be rechallenged with study medication. Rechallenges must be discussed with, and approved by, the Parke-Davis or Pfizer clinical monitor (or their designee) and appropriate IRB/EC. These patients will maintain their original treatment group assignment during rechallenge. A rechallenge is defined as reinstitution of study drug following discontinuation for a medically significant adverse event considered to be caused by atorvastatin. (See Section 6.4.2.3 for further details)

NEW>

June 13, 2003 – Amendment 4

If either of the above abnormalities occur, the ***<letter, sentence, paragraph deleted>*** Pfizer study manager or clinical monitor (or their designees) should be contacted immediately.

Following normalization of a clinically important laboratory abnormality considered causally related to atorvastatin, the patient may be rechallenged with study medication.

Rechallenges must be discussed with, and approved by, the ***<letter, sentence, paragraph deleted>*** Pfizer clinical monitor (or their designee) and appropriate IRB/EC. These patients will maintain their original treatment group assignment during rechallenge. A rechallenge is defined as reinstitution of study drug following discontinuation for a medically significant adverse event considered to be caused by atorvastatin. (See Section 6.4.2.3 for further details)

Amended--
OLD>

6.4.2.1. Serious Adverse Events[(d)]

Except as noted previously for clinical end points, a serious adverse event is any experience that suggests a significant hazard, contraindication, side effect, or precaution. A serious adverse event includes an experience that:

- Is fatal or immediately life-threatening.
- Is severely or permanently disabling.
- Requires or prolongs inpatient hospitalization.
- Is a congenital anomaly, cancer, or overdose (intentional or accidental).
- Is a medically significant event (includes laboratory abnormalities).

Hospitalizations for diagnostic procedures performed for underlying coronary disease that are not in association with acute medical events shall not be reported as SAEs provided: 1) The procedure was ordered in routine management of the underlying disease state for which the patient is being treated in the study. 2) The patient was not hospitalized for a worsening of the underlying disease state. 3) The patient was not hospitalized for an acute event which subsequently led to a procedure.

Overdoses with an associated adverse event are to be reported as serious adverse events. Overdoses are defined as follows:

- Pre-Randomization: greater than 8 tablets of open label drug taken in a 24 hour period.
- Post Randomization: greater than 8 tablets from bottle A, or greater than 2 tablets from bottle B, taken in a 24 hour period.

(d) See Appendix D, Administrative Procedures for the Reporting of Adverse Events (Section 4.1, Immediately Reportable Adverse Events)

NEW>
June 13, 2003 – Amendment 4
6.4.2.1. Serious Adverse Events[d]

Except as noted previously for clinical end points, a serious adverse event is any experience that suggests a significant hazard, contraindication, side effect, or precaution. A serious adverse event includes an experience that:

- ***Results in death;***
- ***Is life-threating;***
- ***Results in inpatient hospitalization or prolongation of existing hospitalization***
- ***Results in a persistent or significant disability/incapacity; or***
- ***Results in congenital anomaly/birth defect***

Hospitalizations for diagnostic procedures performed for underlying coronary disease that are not in association with acute medical events shall not be reported as SAEs provided: 1) The procedure was ordered in routine management of the underlying disease state for which the patient is being treated in the study. 2) The patient was not hospitalized for a worsening of the underlying disease state. 3) The patient was not hospitalized for an acute event which subsequently led to a procedure.

Overdoses with an associated adverse event are to be reported as serious adverse events. Overdoses are defined as follows:

- Pre-Randomization: greater than 8 tablets of open label drug taken in a 24 hour period.
- Post Randomization: greater than 8 tablets from bottle A, or greater than 2 tablets from bottle B, taken in a 24 hour period.

Appendix D provides serious adverse event definitions as well as administrative and reporting information.

Amended--
OLD>
6.4.2.2. Adverse Event Follow-up

Adverse events reported during the screening, open-label run-in, double-blind treatment periods and up to 15 days after the cessation of treatment should be recorded and followed until the adverse event has subsided and abnormal findings have returned to normal or stabilized.

[d] See Appendix D, Administrative Procedures for the Reporting of Adverse Events (Section 4.1, Immediately Reportable Adverse Events)

NEW>
June 13, 2003 – Amendment 4
6.4.2.2. Adverse Event Follow-up

Adverse events reported during the screening, open-label run-in, double-blind treatment periods and up to *30* days after the cessation of treatment or ***final follow-up visit required by the protocol (whichever comes later)*** should be recorded and followed until the adverse event has subsided and abnormal findings have returned to normal or stabilized.

Amended---
OLD>
6.4.2.1 Serious Adverse Events[d]
IN CASE OF SERIOUS OR LIFE-THREATENING ADVERSE EVENTS, OR IN THE EVENT OF DEATH, THE PARKE-DAVIS OR PFIZER STUDY MANAGER OR CLINICAL MONITOR (or their designees) SHOULD BE CONTACTED IMMEDIATELY.

If any serious adverse event occurs, study treatment should be interrupted or discontinued at the investigator's discretion except as specified by the protocol. If in an acute medical emergency the Parke-Davis or Pfizer monitor (or their designees) cannot be contacted, the randomization code may be broken only if this is required for proper treatment of the patient.[e] Notify the Parke-Davis or Pfizer Clinical Monitor (or their designees) of emergency code breaks as soon as possible.

NEW>
June 13, 2003 – Amendment 4
IN CASE OF SERIOUS OR LIFE-THREATENING ADVERSE EVENTS, OR IN THE EVENT OF DEATH, THE *<letter, sentence, paragraph deleted>* PFIZER STUDY MANAGER OR CLINICAL MONITOR (or their designees) SHOULD BE CONTACTED IMMEDIATELY.

If any serious adverse event occurs, study treatment should be interrupted or discontinued at the investigator's discretion except as specified by the protocol. If in an acute medical emergency the ***<letter, sentence, paragraph deleted>*** Pfizer monitor (or their designees) cannot be contacted, the randomization code may be broken only if this is required for proper treatment of the patient.[e] Notify the or ***<letter, sentence, paragraph deleted>*** Pfizer Clinical Monitor (or their designees) of emergency code breaks as soon as possible.

---

[d] See Appendix D, Administrative Procedures for the Reporting of Adverse Events (Section 4.1, Immediately Reportable Adverse Events)

[e] See Appendix E, Other Administrative and Regulatory Procedures (Section 1.7, Emergency Information)

[e] See Appendix E, Other Administrative and Regulatory Procedures (Section 1.7, Emergency Information)

Amended--

OLD>

6.5 Patient Follow-up/Discontinuation of Study Drug

If necessary, an independent search firm will be contracted to locate patients who are lost-to-follow-up at the end of the study to determine if the patient is alive or deceased.

Clearly indicate on the appropriate CRF the reason for withdrawal from the study.

NEW>

June 13, 2003 – Amendment 4

If necessary, an independent search firm will be contracted to locate patients who are lost-to-follow-up at the end of the study to determine if the patient is alive or deceased.

In order to validly assess the safety and efficacy of the two treatment groups, complete follow-up of all randomized patients is critical. At a minimum, the vital status of every randomized patient must be determined upon study completion. Within local laws and regulations, all sources of data (direct patient follow-up, telephone contact, mail questionnaires, and public sources of information will be utilized in order to ascertain the vital status of each randomized patient.

Clearly indicate on the appropriate CRF the reason for withdrawal from the study.

Amended--

OLD>

6.7 Study Completion[f]

January 15, 1999 - Amendment 2

Unless the study is stopped early as described in Section 9.6.2, patients will complete their participation in the study when the number of primary end points is achieved (n = 750) or until an average 5 years duration of therapy is obtained, at which time all patients will have a final annual visit. Clearly indicate on the appropriate CRF the completion date for each patient.

NEW>

June 13, 2003 – Amendment 4

6.7. Study Completion[f]

Unless the study is stopped early as described in Section 9.6.2, patients will complete their participation in the study when the number of ***major coronary events (CHD death, non-fatal myocardial infarction, or resuscitated cardiac arrest)*** is achieved (n = 750) ***<letter/sentence/paragraph deleted>***, at which time all patients will have a final annual visit ***(if an annual visit has not been completed in the past 3 months). Subjects not***

[f] See Appendix E, Other Administrative and Regulatory Procedures (Section 3.3, Study Termination)
[f] See Appendix E, Other Administrative and Regulatory Procedures (Section 3.3, Study Termination)

requiring a final clinic visit will be contacted for a final study follow-up/non-clinic visit. Clearly indicate on the appropriate CRF the completion date for each patient.

Amended--
OLD>

7.1 Formulation

The Clinical Pharmaceutical Operations Department of Parke-Davis will provide medication assembled for each patient based on a randomization code prepared by the Biometrics Department (or their designee).

NEW>
June 13, 2003 – Amendment 4

7.1 Formulation

Pfizer Pharmacy Operations or their designee will provide medication assembled for each patient based on a randomization code prepared by the Biometrics Department (or their designee).

Amended--
OLD>

8. DATA COLLECTION

CRFs for all patients will be supplied by Parke-Davis Pharmaceutical Research, Pfizer Central Research or their designees. These are to be completed as instructed.[h] Original source documents, CRFs, and other study documents must be maintained at the site as specified.[i] Regular monitoring will be performed by Parke-Davis Pharmaceutical Research, Pfizer Central Research or their designees.[j] CRFs will be collected at regular intervals.

NEW>
June 13, 2003 – Amendment 4

8. DATA COLLECTION

CRFs for all patients will be supplied by ***<letter, sentence, paragraph deleted>*** Pfizer***<letter, sentence, paragraph deleted>***, or their designees. These are to be completed as instructed.[h] Original source documents, CRFs, and other study documents must be maintained at the site as specified.[i] Regular monitoring will be performed by

---

[h] See Appendix E, Other Administrative and Regulatory Procedures (Section 2.2, Guidelines for Recording Data in the Case Report Forms)

[i] See Appendix E, Other Administrative and Regulatory Procedures (Section 2.1.4, Retention of Study Records)

[j] See Appendix E, Other Administrative and Regulatory Procedures (Sections 1.4, Monitoring the Study and 2.3, Review of Case Report Forms)

[h] See Appendix E, Other Administrative and Regulatory Procedures (Section 2.2, Guidelines for Recording Data in the Case Report Forms)

[i] See Appendix E, Other Administrative and Regulatory Procedures (Section 2.1.4, Retention of Study Records)

<letter, sentence, paragraph deleted> Pfizer***<letter, sentence, paragraph deleted>***, or their designees.[j] CRFs will be collected at regular intervals.

Amended---

OLD>

9.1 Sample Size Considerations

The sample size estimation is based on the primary efficacy endpoint, major coronary events, as defined previously.

A group sequential test of the null hypothesis of equal incidence of major coronary events with 2 interim analyses using a Peto type alpha-spending is planned.

Assuming a 5 year cumulative event rate in the atorvastatin 80 mg group of 7.02%, a 5 year cumulative event rate in the atorvastatin 10 mg group of 9%, and no dropouts, drop-ins, or noncompliance, a sample size of 6,928 subjects would have 91% power for a one-sided test at alpha of 0.05 and 85% power for a two-sided test at alpha of 0.05. In order to compensate for an estimated 10% dropout/drop-in/noncomplier rate, the study will have a target enrollment of approximately 8,600 subjects [$6{,}928/(1-0.1)^2 = 8{,}553$] (Lachin, JM, *Controlled Clinical Trials* 2, 93-113 (1981)).

The group sequential boundaries were computed using EaSt, by Cytel Software, Cambridge, MA USA. The power calculations were performed using n-Query Advisor, Version 2.0, by Janet D. Elashoff.

NEW>

<u>June 13, 2003 – Amendment 4</u>

<Letter, sentence, paragraph deleted>

Assuming a 5 year cumulative ***major coronary*** event rate in the atorvastatin 80 mg group of 7.02%, a 5 year cumulative ***major coronary*** event rate in the atorvastatin 10 mg group of 9%, and no dropouts, drop-ins, or noncompliance, a sample size of 6,928 subjects would have 91% power for a one-sided test at alpha of 0.05 and 85% power for a two-sided test at alpha of 0.05. In order to compensate for an estimated 10% dropout/drop-in/noncomplier rate, the study will have a target enrollment of approximately 8,600 subjects [$6{,}928/(1-0.1)^2 = 8{,}553$] ***in order to accumulate the target number of 750 major coronary events in an average follow-up time estimated at 5.5 years*** (Lachin, JM, *Controlled Clinical Trials* 2, 93-113 (1981)).

The sample size estimation shown above was based on assumptions regarding incidence rates for major coronary events (the primary endpoint excluding stroke). It is anticipated that approximately 950 primary endpoints (an additional 200 stroke events) will accrue over the total expected duration of the trial, thereby providing 91% power for a one-sided test at alpha of 0.05 and 85% power for a two-sided test at alpha

[j] See Appendix E, Other Administrative and Regulatory Procedures (Sections 1.4, Monitoring the Study and 2.3, Review of Case Report Forms)

of 0.05 to detect a 17% reduction in the 5 year cumulative primary endpoint rate for atorvastatin 80 mg relative to atorvastatin 10 mg.

<letter, sentence, paragraph deleted> The power calculations were performed using n-Query Advisor, Version 2.0, by Janet D. Elashoff.

The steering committee will examine blinded incidence, dropout rate, and other key assumption information when approximately 2 years of completed follow-up data is available and decide whether the proposed target number of events (described in Section 6.1) is appropriate. The sponsor will be approached if a change is proposed.

Amended---
OLD>

9.2. Analysis Populations

The primary efficacy population will consist of all randomized subjects who took at least one dose of blinded study drug and had at least one post-baseline assessment of the primary clinical endpoint, occurrence of a major coronary event, irrespective of any other deviations from the protocol or premature discontinuation.

The primary safety population will consist of all randomized subjects who took at least one dose of blinded study medication and were not lost to follow-up immediately post-randomization.

NEW>
June 13, 2003 – Amendment 4

9.2. Analysis Populations

The primary efficacy ***and safety*** population will consist of all randomized subjects who ***were dispensed*** at least one dose of blinded study drug ***<letter, sentence, paragraph deleted>*** irrespective of any ***<letter, sentence, paragraph deleted>*** deviations from the protocol or premature discontinuation.

<Letter, sentence, paragraph deleted>.

Amended---
OLD>

9.3. Efficacy and Safety Variables: Definitions

NEW>
June 13, 2003 – Amendment 4

9.3. Efficacy Parameters

Amended--
OLD>

9.3.1. Primary Efficacy: Major Coronary Events

Major coronary events will be defined to have occurred according to the judgement of the End Point Committee. Incidence rates are defined as the proportion of the primary efficacy population in each treatment group who experience major coronary events.

NEW>
June 13, 2003 – Amendment 4

9.3.1 Primary Efficacy: Time to the Occurrence of Major Cardiovascular Events

<Letter, sentence, paragraph deleted>The time to occurrence of a major cardiovascular event (CHD death, non-fatal myocardial infarction, resuscitated cardiac arrest, fatal or non-fatal stroke) is defined as the period of time elapsing between the first day of blinded study drug dispensed and the first day of documented occurrence of the major event.

Amended--
OLD>

9.3.2 Primary Safety: Elevated ALT and AST, CPK with Muscle Symptoms

The post-randomization incidence of elevated ALT, AST, and CPK with muscle symptoms (defined in section 6.4.1.1.) will be computed as a proportion of the primary safety population.

9.3.3. Secondary Efficacy: Definitions

9.3.3.1. Time to the Occurrence of Major Coronary Events

The time to occurrence of major coronary events is defined as the period of time elapsing between the day of first blinded study drug administration and the (first) day of documented recurrence.
If a subject dies from a cause not related to major coronary events or study drug, then the time to event for that subject will be considered censored as if the subject had been lost to follow-up at that point. The censoring time will be taken as the time point of the latest documentation of event-free status.

Subjects who drop out will be followed to assess the time of occurrence of the end point. Subjects who are known not to have experienced an end point by the time of the data cutoff for the final analysis will be considered to be censored at the time of the data cutoff. Subjects who are lost to follow-up without experiencing an end point will be considered censored at the time of their last contact.

9.3.3.2. Other Cardiovascular Morbidity and Mortality

The incidence of the following events will be computed as a proportion of the efficacy population experiencing them.

-any coronary event (primary endpoint or, CABG, PTCA, other revascularization procedure or documented angina)
-cerebrovascular event (fatal or non-fatal stroke, TIA)
-peripheral vascular disease (PVD)
-hospitalization with primary diagnosis of CHF
-any cardiovascular event (any of the above)

9.3.3.3. All Cause Mortality

The all cause mortality endpoint is defined as any death from any cause occurring after randomization and before the announced end of the study or data cutoff for analysis. Subjects who drop out will be followed for all-cause mortality. A censoring event occurs if and only if the subject is lost to follow-up. At the announced end of the study, subjects still living will contribute censored observations; the censoring time will be the end of study or data cutoff date.

9.3.3.4. Changes in Serum Lipids

Changes in serum lipid levels after 1, 2, 3, 4, and 5 years' treatment will be defined in two ways. The first is percent change from baseline (average of Visits 4 and 5 measurements when available) and the second is percent change from Visit 2. Lipids and lipoproteins to be analyzed are total cholesterol, LDL-cholesterol, HDL-cholesterol, triglycerides and apolipoproteins A1 and B.

9.4. General Safety

9.4.1. Adverse Experiences

Incidence of post-randomization adverse experiences will be computed as a fraction of the primary safety subpopulation.

9.4.2. Laboratory Values

The incidence of abnormalities will be computed as a fraction of the primary safety subpopulation.

9.5. Methods of Analysis

9.5.1. Primary Efficacy

The null hypothesis is that the probabilities of experiencing major coronary events are equal for the atorvastatin 10 and 80 mg groups. The one-sided alternative is that the

probability is lower for atorvastatin 80 mg treated subjects, and the two-sided alternative is that the probabilities are not equal for the atorvastatin 10 and 80 mg groups.

The primary hypothesis test will be carried out by means of Pearson's Chi Square test. Additionally, the relative risk point estimate and corresponding confidence interval will be reported for the whole cohort as well as for subgroups defined below.

It is not anticipated that enrollment by center will be sufficiently uniform to permit the straightforward interpretation of any analysis that is blocked by center. If any center(s) should contribute data on 50 or more subjects, then separate estimates of the incidence and relative risk for such centers will be reported.

9.5.2. Primary Safety

Point and interval estimates of the incidence and relative risks of elevated AST, ALT, and CPK with muscle symptoms will be reported.

9.5.3. Secondary Efficacy

9.5.3.1. Subgroup, Interaction Analyses

Secondary analyses will examine the effects of the covariables age, gender, smoking status, presence or absence of diabetes, TG (< 200, ≥ 200 mg/dl), CHF, other prevalent pre-existing condition categories of the study cohort, and prevalent concomitant medication use including hormone replacement therapy, aspirin, beta-blockers, or calcium channel blockers. The objective of these analyses will be to determine whether there are any interactions between treatment and these covariables. Estimates of treatment effect will be reported for the subgroups defined by these covariables.

9.5.3.2. Changes in Serum Lipids

The percent changes from baseline and Visit 2 will be compared via the Mann-Whitney-Wilcoxon rank sum statistic at a significance level of 0.05.

9.5.3.3. Time to Major Coronary Event and Overall All-Cause Mortality

Product limit estimators for time to the occurrence of a major coronary event and survival (all cause mortality) in each treatment group will be prepared and displayed graphically. Equality of the time-to-event distributions for the period of the study will be compared by the logrank test at a significance level of 0.05 and, if appropriate, the hazard ratio will be estimated by means of Cox regression (treatment-only model). Additional models including covariable terms will be examined if appropriate.

9.5.4. General Safety

9.5.4.1. Adverse Experiences

Adverse events will be coded and grouped by body system. The incidence of adverse events reported during the study will be reported by first occurrence and by total number of reports. These events will be further summarized by severity and relationship to treatment.

9.5.4.2. Laboratory Values

Incidence rates for abnormal values will be displayed by treatment.

NEW>
June 13, 2003 – Amendment 4
<Letter, sentence, paragraph deleted from 9.3.2 thru 9.5.4.2>
9.3.2 Secondary Efficacy: Definitions

9.3.2.1 Other Cardiovascular Morbidity and Mortality

The time to occurrence of the following events will be calculated as the period of time elapsing between the first day of blinded study drug dispensed and the first day of documented occurrence of:

a. ***Major coronary event (CHD death, non-fatal myocardial infarction, or resusciltated cardiac arrest).***
b. ***Any coronary event major coronary event or, CABG, PTCA, other revascularization procedure, procedure-related myocardial infarction or documented angina)***
c. ***cerebrovascular event (fatal or non-fatal stroke, TIA)***
d. ***peripheral vascular disease (PVD)***
e. ***hospitalization with primary diagnosis of CHF***
f. ***any cardiovascular event (any of the above)***

9.3.2.2 All Cause Mortality

The time to occurrence of all cause mortality will be calculated as the period of time elapsing between the first day of blinded study drug dispensed and the day of documented occurrence of death.

9.4. Methods of Analysis

All hypothesis testing will be two-sided at a significance level of 0.05 unless otherwise indicated.

Subjects who discontinue study medication will continue to be followed to assess the time to occurrence of each endpoint. Subjects who do not experience endpoints during

the trial will be considered censored at the time of the data cut-off, death or last contact, whichever is earliest.

9.4.1. Primary Efficacy Analysis

The null hypothesis that there is no difference in the time to occurrence of a major cardiovascular event between the two treatment groups will be examined using a log rank test. The product limit estimates for the time to occurrence will be calculated and displayed graphically as "survival" curves.

9.4.2. Secondary Efficacy Analysis

A log rank test will be used to examine the differences in time to occurrence between the two treatment groups for the other cardiovascular morbidity and mortality variables and all cause mortality.

Risk ratios and 95% confidence limits will be estimated for the primary and secondary efficacy endpoints using Cox regression.

9.4.3. Covariate and Subgroup Analyses

Cox regression models will be used to examine the treatment effect adjusted for various covariates, and to examine the treatment by covariate interaction effects. Covariates to be examined include age, gender smoking status, presence or absence of diabetes, baseline triglycerides, baseline HDL, baseline LDL, CHF, other prevalent pre-existing condition categories of the study cohort, and prevalent concomitant medication use (such as hormone replacement therapy, aspirin, beta-blockers, calcium channel blockers, etc.). In addition, the above analyses will be used to examine subgroups of interest as specified above, such as women and revascularized patients.

9.5. Safety Parameters

Adverse events will be coded and grouped by body system. The incidence of adverse events reported during the study will be tabulated according to the number of affected patients as well as the number of events. The intensity and relationship to study drug of adverse events will also be summarized by body system and treatment groups.

The incidence of shifts from normal at baseline to abnormal at study completion will be summarized both overall and by direction for all safety laboratories by treatment group.

In addition, changes in serum lipid levels after 1, 2,3,4 and 5 years of treatment will be calculated for each treatment group both as percent change from baseline (the average of the values at Visit 4 and Visit 5) and as percent change from Visit 2. Serum lipids include total cholesterol, LDL, HDL, triglycerides, apolipoproteins A1 and B, and non-HDL. Further analysis to determine the relationship between the occurance of endpoints and these lipid parameters will be investigated.

Amended---

OLD>

9.6.1. Timing, Content

Interim analyses to examine efficacy differences will be performed at information times approximately equal to 0.5 and 0.75.

The interim analyses will include analyses of recruitment, subject characteristics, the primary efficacy and safety variables, all-cause mortality, and adverse events.

NEW>

June 13, 2003 – Amendment 4

9.6.1. Timing, Content

Interim analyses to examine ***primary*** efficacy ***<letter, sentence, paragraph deleted>*** will ***begin after a median of 3 years of follow-up. The timing and number of interim analyses will be at the discretion of the DSMB.***

The interim analyses will include analyses of recruitment, subject characteristics, the primary efficacy and safety variables, all-cause mortality, and adverse events.

Amended---

OLD>

9.6.2. Early stopping

The chi square statistic for the hypothesis test of the null hypothesis of no difference in the probability of occurrence of major coronary events between atorvastatin 10 and 80 mg groups will be compared to the critical boundary values. If the group sequential boundary is crossed, then the DSMB will consider a recommendation to stop the trial.

However, actual stopping of the trial is a complex decision involving a number of factors besides the simple comparison of one statistic with one critical value. The DSMB will take all the data together and analyze the data in concert including performing additional analyses before making a recommendation that the trial be stopped. A partial list of factors besides the magnitude of an apparent treatment effect that should be taken into account include the clinical significance of an apparent effect, whether an apparent effect could be explained by baseline imbalance or concomitant therapy, whether occurrence of major coronary events is consistent with other signals in the data, whether results are consistent within subgroups and centers, the likelihood of a reversal in the current trend, and the needs of the medical research community and future patients for a definitive answer to the research questions.

In summary, a recommendation to modify or terminate the trial would not be based totally on the crossing of a group-sequential boundary.

The DSMB would always have discretionary power to recommend stopping the trial if it felt that the safety of the subjects was at risk.

NEW>
June 13, 2003 – Amendment 4
9.6.2. Early stopping

The ***log-rank*** statistic for the hypothesis test of the null hypothesis of no difference in the probability of occurrence of major ***cardiovascular*** events between atorvastatin 10 and 80 mg groups will be compared to the critical boundary values ***based on a two-sided Peto type monitoring boundary with a critical value (Z) of ± 3.0.*** If the group sequential boundary is crossed, then the DSMB will consider a recommendation to stop the trial.

However, actual stopping of the trial is a complex decision involving a number of factors besides the simple comparison of one statistic with one critical value. The DSMB will take all the data together and analyze the data in concert including performing additional analyses before making a recommendation that the trial be stopped. A partial list of factors besides the magnitude of an apparent treatment effect that should be taken into account include the clinical significance of an apparent effect, whether an apparent effect could be explained by baseline imbalance or concomitant therapy, whether occurrence of major coronary events is consistent with other signals in the data, whether results are consistent within subgroups and centers, the likelihood of a reversal in the current trend, and the needs of the medical research community and future patients for a definitive answer to the research questions.

Interim monitoring for harm will be based on all-cause mortality using a log-rank test with an O'Brien-Fleming boundary (two-sided α= .05) truncated to ±3.0. If the group sequential boundary is crossed, then the DSMB will consider a recommendation to stop the trial. The DSMB will take all the data together and analyze the data in concert including performing additional analyses before making a recommendation that the trial be stopped for harm.

In summary, a recommendation to modify or terminate the trial would not be based totally on the crossing of a group-sequential boundary.

<Letter, sentence, paragraph deleted>

Amended--
OLD>
11.2 PROCEDURE

[redacted] (US Sites) or [redacted]

NEW>

June 13, 2003 – Amendment 4

11.2 PROCEDURE

[redacted] [redacted]

ATTN: [redacted] 32 Excelsiorlaan

[redacted] [redacted]

[redacted]

Amended--

OLD>

Total cholesterol
LDL-C[a]
HDL-C
VLDL-C[b]
TG

---

a At all designated visits, LDL-C will be estimated by the Friedewald formula unless the TG value at that visit is >400 mg/dL (>4.5 mmol/l) at which point the LDL-C will be measured directly.

b VLDL-C will be calculated as total cholesterol - [HDL-C] - [LDL-C] whenever LDL-C is measured directly or will be estimated as TG/5 when the Friedewald formula is used.

NEW>

June 13, 2003 – Amendment 4

Total cholesterol
LDL-C[a] [c]
HDL-C
VLDL-C[b]
TG

---

a At all designated visits, LDL-C will be estimated by the Friedewald formula unless the TG value at that visit is >400 mg/dL (>4.5 mmol/l) at which point the LDL-C will be measured directly.

b VLDL-C will be calculated as total cholesterol - [HDL-C] - [LDL-C] whenever LDL-C is measured directly or will be estimated as TG/5 when the Friedewald formula is used.

c ***At all annual visits where LDL-C < 50 mg/dl (1.3 mmol/l), a direct LDL measurement will be completed. All LDL results will remain blinded.***

Amended---
OLD>

TABLE OF CONTENTS

(Page 1 of 3)

Page

SYNOPSIS ii

1. INTRODUCTION 1

2. STUDY OBJECTIVE 4

3. SUMMARY OF STUDY 4

4. ETHICAL AND LEGAL CONSIDERATIONS 5

5. STUDY POPULATION 5
5.1. Source and Number of Patients 5
5.2. Patient-Selection Criteria 5
5.2.1. Inclusion Criteria 6
5.2.2. Exclusion Criteria 7
5.3. Prohibited Medications or Precautions 8
5.4. Allowable Concurrent Medications 9

6. STUDY DESIGN AND METHODOLOGY 9
6.1. Study Design 9
6.2. Study Schedule and Guidelines for Visits 9
6.2.1. Screening Visit (Visit 1) 10
6.2.2. Atorvastatin 10 mg Run-In Period 11
October 6, 1998 Amendment 1
6.2.3. Rescreening and Retesting Prior to Randomization 12
6.2.4 Double-Blind Active Treatment Period 12
6.3. Efficacy Assessments 13
6.3.1. Primary Efficacy Parameters 13
6.3.2. Secondary Efficacy Parameters 14
6.3.3. End Point Committee 14

6.3.4. Clinical End Point Documentation 14
6.4. Safety Assessments 14
6.4.1. Laboratory Evaluation 15
6.4.1.1. Clinically Important Laboratory Abnormalities 16

TABLE OF CONTENTS
(Page 2 of 3)

6.4.2. Adverse Event Reporting 17
6.4.2.1. Serious Adverse Events 18
6.4.2.2. Adverse Event Follow-up 19
6.4.3. Other Safety Monitoring 20
6.4.4. Data and Safety Monitoring Board (DSMB) 20
6.4.5. Steering Committee 21
6.5. Economic Evaluation 21
6.6. Patient Follow-up/Discontinuation of Study Drug 21
6.7. Study Completion 22

7. STUDY MEDICATION 22
7.1. Formulation 22
7.2. Medication Dispensing 23
7.3. Dosage Regimen 23

8. DATA COLLECTION 23

9. DATA ANALYSIS AND STATISTICAL CONSIDERATIONS 23
9.1. Sample Size Considerations 24
9.2. Analysis Populations 25
9.3. Efficacy and Safety Variables: Definitions 25
9.3.1. Primary Efficacy: Major Coronary Events 25
9.3.2. Primary Safety: Elevated ALT and AST, CPK with Muscle Symptoms 25
9.3.3. Secondary Efficacy: Definitions 25
9.3.3.1. Time to the Occurrence of Major Coronary Events 25
9.3.3.2. Other Cardiovascular Morbidity and Mortality 26
9.3.3.3. All Cause Mortality 26
9.3.3.4. Changes in Serum Lipids 26
9.4. General Safety 26
9.4.1. Adverse Experiences 26

9.4.2. Laboratory Values 26
9.5. Methods of Analysis 26
9.5.1. Primary Efficacy 26

TABLE OF CONTENTS
(Page 3 of 3)

9.5.2. Primary Safety 27
9.5.3. Secondary Efficacy 27
9.5.3.1. Subgroup, Interaction Analyses 27
9.5.3.2. Changes in Serum Lipids 27
9.5.3.3. Time to Major Coronary Event and Overall All-Cause Mortality 27
9.5.4. General Safety 27
9.5.4.1. Adverse Experiences 28
9.5.4.2. Laboratory Values 28
9.6. Interim Analyses 28
9.6.1. Timing, Content 28
9.6.2. Early Stopping 28
9.6.3. Distribution of the Interim Analysis 29

10. ECONOMIC ANALYSES 29

June 4, 1999 - Amendment 3

11. DNA Databank* *29
11.1 Introduction* *29
11.2 Procedure* *29
11.3 Process for Anonymity of Blood Samples* *30

12. REFERENCES* *30

13. LIST OF APPENDICIES* *34
A. Timetable of Visits and Procedures 35
B. Commonly Prescribed Prohibited Medications 43
C. Study Safety Procedures and Clinical Laboratory Determinations 46
October 6, 1998 - Amendment 1
D. Administrative Procedures and Clinical Laboratory Determinations .... D-i
E. Other Administrative and Regulatory Procedures .... E-i
F. Declaration of Helsinki .... F-i
G. Amendments - October 6, 1998 .... *G-1*

January 15, 1999 - Amendment 2
H. Amendments - January 15, 1999 .... H-1
June 4, 1999 – Amendment 3
I. Amendments – June 4, 1999

NEW>
June 13, 2003 – Amendment 4

TABLE OF CONTENTS

(Page 1 of 3)

Page

SYNOPSIS ii

1. INTRODUCTION 1

2. STUDY OBJECTIVE 4

3. SUMMARY OF STUDY 4

4. ETHICAL AND LEGAL CONSIDERATIONS 4

5. STUDY POPULATION 5
5.1. Source and Number of Patients 5
5.2. Patient-Selection Criteria 5
5.2.1. Inclusion Criteria 5
5.2.2. Exclusion Criteria 6
5.3. Prohibited Medications or Precautions 7
5.4. Allowable Concurrent Medications 8

6. STUDY DESIGN AND METHODOLOGY 8
6.1. Study Design 8
6.2. Study Schedule and Guidelines for Visits 9
6.2.1. Screening Visit (Visit 1) 9
6.2.2. Atorvastatin 10 mg Run-In Period 10
6.2.3. Rescreening and Retesting Prior to Randomization 11
6.2.4 Double-Blind Active Treatment Period 12
6.3. Efficacy Assessments 12
6.3.1. Primary Efficacy Parameters 13
6.3.2. Secondary Efficacy Parameters 13
6.3.3. End Point Committee 13

6.3.4. Clinical End Point Documentation 14
6.4. Safety Assessments 14
6.4.1. Laboratory Evaluation 14
6.4.1.1. Clinically Important Laboratory Abnormalities 15

TABLE OF CONTENTS

(Page 2 of 3)

6.4.2. Adverse Event Reporting 16
6.4.2.1. Serious Adverse Events 17
6.4.2.2. Adverse Event Follow-up 18
6.4.2.3 Rechallenge Procedure 18
6.4.3. Other Safety Monitoring 19
6.4.4. Data and Safety Monitoring Board (DSMB) 19
6.4.5. Steering Committee 20
6.5. Economic Evaluation 20
6.6. Patient Follow-up/Discontinuation of Study Drug 20
6.7. Study Completion 21

7. STUDY MEDICATION 21
7.1. Formulation 21
7.2. Medication Dispensing 22
7.3. Dosage Regimen 22

8. DATA COLLECTION 22

9. DATA ANALYSIS AND STATISTICAL CONSIDERATIONS 22
9.1. Sample Size Considerations 22
9.2. Analysis Populations 24
9.3. Efficacy Paramaters 24
9.3.1. Primary Efficacy: Time to the Occurrence of Major Cardiovascular Events. 24
9.3.2. Secondary Efficacy: Definitions 24
9.3.2.1 Other Cardiovascular Morbidity and Mortality 24
9.3.2.2 All Cause Mortality 25
9.4. Methods of Analysis 25
9.4.1. Primary Efficacy Analysis 25
9.4.2. Secondary Efficacy 25
9.4.3 Covariate and Subgroup Analyses 25
9.5. Safety Parameters 25

TABLE OF CONTENTS

(Page 3 of 3)

9.6. Interim Analyses 26
9.6.1. Timing, Content 26
9.6.2. Early Stopping 26
9.6.3. Distribution of the Interim Analysis 27

10. ECONOMIC ANALYSES 27

11. DNA Databank 27
11.1 Introduction 27
11.2 Procedure 27
11.3 Process for Anonymity of Blood Samples 28

12. REFERENCES 28

13. LIST OF APPENDICIES 32
A. Timetable of Visits and Procedures 33
B. Commonly Prescribed Prohibited Medications 43
C. Study Safety Procedures and Clinical Laboratory Determinations 46
D. Administrative Procedures and Clinical Laboratory Determinations .... D-i
E. Other Administrative and Regulatory Procedures .... E-i
F. Declaration of Helsinki .... F-i
G. Amendments - October 6, 1998 .... G-1
H. Amendments - January 15, 1999 .... H-1
I. Amendments – June 4, 1999 .... I-1
J. Amendments – June 13, 2003 .... J-1

Amended---
OLD>

Visits 9,11,13,15,17,19 (and at 12 month intervals thereafter as required)

- Physical examination
- Draw blood samples for full clinical laboratory, lipid profile (special lipids at Visit 9 only), hematology, HbA1C (known/suspected diabetics), and plasma storage
- Vital signs
- Urinalysis
- ECG
- Continue dietary counseling

- Dispense study medication (not required at final visit)
- Record adverse events
- Record potential end points
- Record changes in and additions to concurrent medications
- Pregnancy test for women of child-bearing potential

NEW>

June 13, 2003 – Amendment 4

Visits 9,11,13,15,17,19 (and at 12 month intervals thereafter as required) *and Study Completion*

- Physical examination
- Draw blood samples for full clinical laboratory, lipid profile (special lipids at Visit 9 only), hematology, HbA1C (known/suspected diabetics), and plasma storage
- Vital signs
- Urinalysis
- ECG
- Continue dietary counseling
- Dispense study medication (not required at final visit)
- Record adverse events
- Record potential end points
- Record changes in and additions to concurrent medications
- Pregnancy test for women of child-bearing potential

Amended--
OLD>
APPENDIX D Amendments

INTRODUCTION

The administrative procedures for reporting adverse events described in this appendix are to be followed during the conduct of this protocol.

If you have any questions concerning adverse event reporting, please contact the Parke-Davis colleague or representative who is monitoring your site or a Parke-Davis Clinical/Medical Colleague whose name, address, and telephone number appears on the cover sheet to this protocol.

NEW>
June 13, 2003 – Amendment 4
INTRODUCTION

The administrative procedures for reporting adverse events described in this appendix are to be followed during the conduct of this protocol.

If you have any questions concerning adverse event reporting, please contact the ***Pfizer Inc and its affiliates*** colleague or representative who is monitoring your site or a ***Pfizer Inc and its affiliates*** Clinical/Medical Colleague whose name, address, and telephone number appears on the
cover sheet to this protocol.

Amended--
OLD>

TABLE OF CONTENTS

Page
1. ADVERSE EVENTS DURING THE STUDY D-1

2. DEFINITIONS D-1
2.1. Attributes of Adverse Events D-3
2.1.1. Treatment-Emergent Signs and Symptoms (TESS) D-3
2.1.2. Serious Adverse Events D-3
2.1.3. Intensity D-3
2.1.4. Relationship to Study Drug—Physician's Assessment D-4
2.1.5. Clinical Outcome D-4

3. CAPTURING ADVERSE EVENTS D-4
3.1. Pre-Existing Condition D-4
3.2. Lack of Efficacy D-5
3.3. Clinical Laboratory Adverse Event D-5
3.4. Hospitalization or Surgery/Procedure D-5

3.5. Death D-5
3.6. General Physical Examination Findings D-5
4. REPORTING TO THE SPONSOR. D-5
4.1. Immediately Reportable Adverse Events D-5
4.2. Other Adverse Events D-6
4.3. Follow-up Period D-6

NEW>

June 13, 2003 – Amendment 4

TABLE OF CONTENTS

Page

1. STANDARD SAFETY SECTION FOR INCLUSION IN PFIZER-SPONSORED CLINICAL TRIAL PROTOCOLS D4
1.1. Adverse Events D4
1.2. Serious Adverse Events D5
1.3. Abnormal Laboratory Test Results D7
1.4. Abnormal Physical Examination Findings D8
1.5. Discontinuations D9

2. DEFINITIONS D-9
2.1. Attributes of Adverse Events D-10
2.1.1. Treatment-Emergent Signs and Symptoms (TESS) D-10
2.1.2. Serious Adverse Events D-10
2.1.3. Intensity D-10
2.1.4. Relationship to Study Drug—Physician's Assessment D-11
2.1.5. Clinical Outcome D-12

3. CAPTURING ADVERSE EVENTS D-12
3.1. Pre-Existing Condition D-12
3.2. Lack of Efficacy D-12
3.3. <letter/sentence/paragraph deleted> Hospitalization or Surgery/Procedure . D-13
3.4. Death D-13
3.5. General Physical Examination Findings D-13

4. REPORTING TO THE SPONSOR. D-13
4.1. Immediately Reportable Adverse Events D-13
4.2. Other Adverse Events D-14
4.3. Follow-up Period D-14

Amended---
OLD>

1. ADVERSE EVENTS DURING THE STUDY

Each subject/patient will be observed and queried in a nonspecific fashion by the investigator or study coordinator at each visit during baseline, study treatment, and protocol-defined follow-up for any new or continuing adverse events (AEs) since the previous visit. Any new AEs or ones changing in character, frequency, or in intensity reported by the subject/patient or caregiver or noted by the investigator or study coordinator after the signing of the informed consent will be recorded on the AE Case Report Form (CRF).

The investigator will review the clinical laboratory test results in a timely fashion when received from the laboratory. Those results qualifying as AEs as defined in Section 3.3 of this appendix will be recorded on the AE CRF and will be handled according to these administrative procedures for reporting AEs.

The investigator will review concomitant medications being taken by the subject/patient. The AE that led to the administration of any new concomitant medications (not specified in the protocol) will be reported on the AE CRF.

NEW>
June 13, 2003 – Amendment 4
1. Standard safety section for inclusion in pfizer-sponsored clinical trial protocols
1.1. Adverse Events
All observed or volunteered adverse events, regardless of treatment group or suspected causal relationship to study drug, will be recorded on the adverse event page(s) of the case report form.

Events involving adverse drug reactions, illnesses with onset during the study, or exacerbations of pre-existing illnesses should be recorded. Exacerbation of pre-existing illness, including the disease under study, is defined as a manifestation (sign or symptom) of the illness that indicates a significant increase in the severity of the illness as compared to the severity noted at the start of the trial. It may include worsening or increase in severity of signs or symptoms of the illness, increase in frequency of signs and symptoms of an intermittent illness, or the appearance of a new manifestation/complication. Exacerbation of a pre-existing illness should be considered when a patient/subject requires new or additional concomitant drug or non-drug therapy for the treatment of that illness during the trial. Lack of or insufficient clinical response, benefit, efficacy, therapeutic effect, or pharmacologic action, should not be recorded as an adverse event. The investigator must make the distinction between exacerbation of pre-existing illness and lack of therapeutic efficacy.

In addition, clinically significant changes in physical examination findings and abnormal objective test findings (eg, laboratory, x-ray, ECG) should also be recorded

as adverse events. The criteria for determining whether an abnormal objective test finding should be reported as an adverse event are as follows:

1) *Test result is associated with accompanying symptoms; and/or*
2) *Test result requires additional diagnostic testing or medical/surgical intervention; and/or*
3) *Test result leads to a change in study dosing or discontinuation from the study, significant additional concomitant drug treatment or other therapy; and/or*
4) *Test result leads to any of the outcomes included in the definition of a serious adverse event; and/or*
5) *Test result is considered to be an adverse event by the investigator or sponsor.*

Merely repeating an abnormal test, in the absence of any of the above conditions, does not meet condition #2 above for reporting as an adverse event.

Any abnormal test result that is determined to be an error does not require reporting as an adverse event, even if it did meet one of the above conditions except for condition #4.

For all adverse events, the investigator must pursue and obtain information adequate both to determine the outcome of the adverse event and to assess whether it meets the criteria for classification as a serious adverse event requiring immediate notification to Pfizer or its designated representative (see Section 1.2). For all adverse events, sufficient information should be obtained by the investigator to determine the causality of the adverse event (ie, study drug or other illness). The investigator is required to assess causality and indicate that assessment on the CRF. Follow-up of the adverse event, after the date of therapy discontinuation, is required if the adverse event or its sequelae persist. Follow-up is required until the event or its sequelae resolve or stabilize at a level acceptable to the investigator and the Pfizer clinical monitor or his/her designated representative.

1.2. Serious Adverse Events

All serious adverse events (as defined below), regardless of treatment group or suspected relationship to study drug, must be reported immediately by telephone to the named individuals identified within the protocol text.

A serious adverse event is any adverse drug experience occurring at any dose that:

1) *results in death;*
2) *is life-threatening;*
3) *results in inpatient hospitalization or prolongation of existing hospitalization;*
4) *results in a persistent or significant disability/incapacity; or*
5) *results in congenital anomaly/birth defect.*

Important medical events that may not result in death, be life-threatening, or require hospitalization may be considered serious adverse drug experiences when, based upon appropriate medical judgment, they may jeopardize the patient/subject and may require

medical or surgical intervention to prevent one of the outcomes listed in this definition. Examples of such medical events include allergic bronchospasm requiring intensive treatment in an emergency room or at home, blood dyscrasias or convulsions that do not result in inpatient hospitalization, or the development of drug dependency or drug abuse.

Regardless of the above criteria, any additional adverse experiences which Pfizer personnel or an investigator considers serious should be immediately reported to Pfizer and included in the Corporate adverse events database system.

A life-threatening adverse event is any adverse drug experience that places the patient/subject at immediate risk of death from the reaction as it occurred; ie, it does not include a reaction that, had it occurred in a more severe form, might have caused death.

Initial hospitalization is defined as any inpatient admission (even if less than 24 hours). For chronic or long-term inpatients, inpatient admission also includes transfer within the hospital to an acute/intensive care inpatient unit (eg, from the psychiatric wing to a medical floor, from a medical floor to the coronary care unit, from the neurological floor to the tuberculosis unit).

1) *Inpatient admission in the absence of a precipitating, treatment-emergent, clinical adverse event may meet criteria for "seriousness" but is not an adverse experience and thus is not subject to immediate reporting to Pfizer. For example:*

   i. *Admission for treatment of a pre-existing condition not associated with the development of a new adverse event or with a worsening of the pre-existing condition (eg, for work-up of persistent pretreatment lab abnormality)*
   ii. *Social admission (eg, subject has no place to sleep)*
   iii. *Administrative admission (eg, for yearly physical exam)*
   iv. *Protocol-specified admission during a clinical trial (eg, for a procedure required by the study protocol)*
   v. *Optional admission not associated with a precipitating clinical adverse event (eg, for elective cosmetic surgery)*

   However, if a hospitalization for an unknown event occurs, it should be considered as a serious adverse event.

2) *Inpatient admission does not include the following:*

   i. *Emergency Room/Accident and Emergency/Casualty Department visits*
   ii. *Outpatient/same-day/ambulatory procedures*
   iii. *Observation/short-stay units*
   iv. *Rehabilitation facilities*
   v. *Hospice facilities*
   vi. *Respite care (eg, caregiver relief)*
   vii. *Skilled nursing facilities*

viii. Nursing homes
ix. Custodial care facilities
x. Clinical research/Phase I units

Prolongation of hospitalization is defined as any extension of an inpatient hospitalization beyond the stay anticipated/required in relation to the original reason for the initial admission, as determined by the investigator or treating physician. For protocol-specified hospitalizations in clinical trials, prolongation is defined as any extension beyond the length of stay described in the protocol. Prolongation in the absence of a precipitating, treatment-emergent, clinical adverse event (ie, not associated with the development of a new adverse event or worsening of a pre-existing condition) may meet criteria for "seriousness" but is not an adverse experience and thus is not subject to immediate reporting to Pfizer.

Preplanned treatments or surgical procedures should be noted in the baseline documentation for the entire protocol and/or for the individual patient/subject.

Disability is a substantial disruption of a person's ability to conduct normal life functions.

Any serious adverse event or death must be reported immediately independent of the circumstances or suspected cause if it occurs or comes to the attention of the investigator at any time during the study through the last follow-up visit required by the protocol or 30 days after the last administration of study drug, whichever comes later. Any serious adverse event occurring at any other time after completion of the study must be promptly reported if a causal relationship to study drug is suspected. The only exception to these reporting requirements are serious adverse events that occur during a prerandomization/
washout run-in period, during which either placebo alone is administered, or no active study drug or no protocol-specified background drug is administered.

For all serious adverse events, the investigator is obligated to pursue and provide information as requested by the Pfizer clinical monitor or designated representative in addition to that on the case report form. In general, this will include a description of the adverse event in sufficient detail to allow for a complete medical assessment of the case and independent determination of possible causality. Information on other possible causes of the event, including concomitant medications and illnesses must be provided. The investigator's assessment of causality must also be provided. If causality is unknown and the investigator does not know whether or not study drug caused the event, then it should be attributed to study drug. If the investigator's causality assessment is "unknown but not related to study drug," this should be clearly documented on study records. In the case of a subject death, a summary of available autopsy findings must be submitted as soon as possible to Pfizer or its designated representative. The investigator should ensure that information reported immediately by telephone or other means and information entered in the case report form are accurate and consistent.

1.3. Abnormal Laboratory Test Results
The results of all laboratory tests required by the protocol will be recorded in the subject's case report form. All clinically important abnormal laboratory tests occurring during the study will be repeated at appropriate intervals until they return either to baseline or to a level deemed acceptable by the investigator and the Pfizer clinical monitor (or his/her designated representative), or until a diagnosis that explains them is made. The criteria for determining whether an abnormal objective test finding should be reported as an adverse event are as follows:

1) *Test result is associated with accompanying symptoms, and/or*
2) *Test result requires additional diagnostic testing or medical/surgical intervention, and/or*
3) *Test result leads to a change in study dosing or discontinuation from the study, significant additional concomitant drug treatment or other therapy, and/or*
4) *Test result leads to any of the outcomes included in the definition of a serious adverse event, and/or*
5) *Test result is considered to be an adverse event by the investigator or sponsor.*

Merely repeating an abnormal test, in the absence of any of the above conditions, does not meet condition #2 above for reporting as an adverse event.

Any abnormal test result that is determined to be an error does not require reporting as an adverse event, even if it did meet one of the above conditions except for condition #4.

1.4. Abnormal Physical Examination Findings
Clinically significant changes, in the judgment of the investigator, in physical examination findings (abnormalities) will be recorded as adverse events.

1.5. Discontinuations
The reason for a subject discontinuing from the study will be recorded in the case report form. A discontinuation occurs when an enrolled subject ceases participation in the study, regardless of the circumstances, prior to completion of the protocol. The investigator must determine the primary reason for discontinuation. Withdrawal due to adverse event should be distinguished from withdrawal due to insufficient response according to the definition of adverse event noted earlier. A discontinuation must be reported immediately to the Pfizer clinical monitor or his/her designated representative if it is due to a serious adverse event. The final evaluation required by the protocol will be performed at the time of study discontinuation. The investigator will record the reason for study discontinuation, provide or arrange for appropriate follow-up (if required) for such subjects, and document the course of the subject's condition.

Amended---
OLD>
2. DEFINITIONS

Pre-Existing Condition

A pre-existing condition is one that is present at the start of study treatment.

Baseline

Defined by the Therapeutic Group for each program.

Adverse Event

Any untoward medical occurrence in a patient or clinical investigation subject administered a pharmaceutical product and which does not necessarily have to have a causal relationship with this treatment. An AE can therefore be any unfavorable and unintended sign (including an abnormal laboratory finding), symptom, or disease temporally associated with the use of a medicinal (investigational) product, whether or not related to the medicinal (investigational) product.

Related Adverse Event

An AE where there is a reasonable possibility that the experience may have been caused by drug (Unknown is also considered related).

Serious Adverse Event (SAE)

Any adverse event occurring at any dose that results in any of the following outcomes:
- Death;
- Life-threatening adverse event;
- ?Inpatient hospitalization or prolongation of existing hospitalization;
- Persistent or significant disability/incapacity;
- ?Congenital anomaly/birth defect; and
- ?Medically significant event (includes laboratory abnormalities).

Medically significant events may not be immediately life-threatening or result in death or hospitalization but may jeopardize the patient or may require intervention to prevent one of the outcomes listed in the definition above. Examples of such events are intensive treatment in an emergency room or at home for allergic bronchospasm; blood dyscrasias or convulsions that do not result in hospitalization; or development of drug dependency or drug abuse, or laboratory abnormalities.

The following hospitalizations are not considered SAEs:

- ?For diagnostic or elective surgical procedures for a pre-existing condition;
- ?For therapy of the target disease(s) of the study if the protocol explicitly anticipated
- and defined the symptoms or episodes;
- ?For study efficacy measurement, as defined in the protocol.

Life-Threatening Adverse Event

Any adverse event that places the patient or subject, in the view of the investigator, at immediate risk of death from the reaction as it occurred, ie, it does not include a reaction that, had it occurred in a more severe form, might have caused death.

Unexpected Adverse Event

Any adverse event, the specificity or severity of which is not consistent with the current Investigator's Brochure. For example, under this definition, hepatic necrosis would be unexpected (by virtue of greater severity) if the Investigator's Brochure only referred to elevated hepatic enzymes or hepatitis.

Similarly, cerebral thromboembolism and cerebral vasculitis would be unexpected (by virtue of greater specificity) if the Investigator's Brochure only listed cerebral vascular accidents. "Unexpected," as used in this definition, refers to an AE that has not been previously observed (eg, included in the Investigator's Brochure) rather than from the perspective of such experience not being anticipated from the pharmacological properties of the pharmaceutical product.

Clinical Laboratory Adverse Event

A clinical laboratory abnormality that is regarded as an AE if it has been confirmed by at least 1 repeat test and suggests a disease and/or organ toxicity of a severity that requires active management, eg, change of dose, discontinuation of drug, more frequent follow-up, diagnostic investigation, etc.

Treatment-Emergent Signs and Symptoms (TESS)

Any AE that was not evident during baseline as defined by the study protocol, or that increases in intensity or frequency, or changes in character during treatment.

NEW>
June 13, 2003 – Amendment 4
2. DEFINITIONS
Pre-Existing Condition

A pre-existing condition is one that is present at the start of study treatment.

Baseline

Defined by the ***protocol as applicable to the study.***

<Letter/Sentence/Paragraph Deleted>

Related Adverse Event

An AE where there is a reasonable possibility that the experience may have been caused by drug (Unknown is also considered related).

<Letter/Sentence/Paragraph Deleted>

<Letter/Sentence/Paragraph Deleted>

Unexpected Adverse Event

Any adverse event, the specificity or severity of which is not consistent with the current Investigator's Brochure. For example, under this definition, hepatic necrosis would be unexpected (by virtue of greater severity) if the Investigator's Brochure only referred to elevated hepatic enzymes or hepatitis.

Similarly, cerebral thromboembolism and cerebral vasculitis would be unexpected (by virtue of greater specificity) if the Investigator's Brochure only listed cerebral vascular accidents. "Unexpected," as used in this definition, refers to an AE that has not been previously observed (eg, included in the Investigator's Brochure) rather than from the perspective of such experience not being anticipated from the pharmacological properties of the pharmaceutical product.

<Letter/Sentence/Paragraph Deleted>

Treatment-Emergent Signs and Symptoms (TESS)

Any AE that was not evident during baseline as defined by the study protocol, or that increases in intensity or frequency, or changes in character during treatment.

Amended---
OLD>

2.1.2. Serious Adverse Events

All SAEs, as defined in Section 2, are immediately reportable to the Sponsor within 24 hours of the Investigator's first knowledge of the event (see Section 4.1 of this appendix).

NEW>
<u>June 13, 2003 – Amendment 4</u>
2.1.2. Serious Adverse Events

All SAEs, as defined in Section *1.2*, are immediately reportable to ***Pfizer Inc. and its affiliates. <Letter/Sentence/Paragraph Deleted>***

Amended---
OLD>
2.1.4. Relationship to Study Drug—Physician's Assessment

The categories for the physician's assessment of the causal relationship between study drug and an AE are Yes, No, and Unknown.

NEW>

June 13, 2003 – Amendment 4

2.1.4. Relationship to Study Drug—Physician's Assessment

There are 6 categories for the physician's assessment of the causal relationship between study drug and an AE:

Physician's Assessment	*Causal Relationship Reported to Regulatory Agencies*
Definitely	*Yes*
Probably	*Yes*
Possibly	*Yes*
Unlikely	*No*
Definitely Not	*No*
Unknown[a]	*Yes (ie, interpreted as "possibly" related)*

[a] ***Insufficient information or indeterminable, too***

The following criteria are to be used to assess the causal relationship between an AE and study drug:

Definite:

Follows a reasonable temporal sequence from administration of the drug or from drug levels established in body fluids or tissues; AE improves or disappears after stopping or reducing the dosage (dechallenge); AE reappears with rechallenge.

or

Is known to be associated with the drug or class of compounds and cannot be explained by other therapy or subject/patient's physical condition.

Probable:

Follows a reasonable temporal sequence from drug administration; AE improves or disappears after stopping or reducing the dosage (dechallenge) and rechallenge was not attempted; AE cannot be explained by other therapy or the subject/patient's physical condition.

Possible (Reasonable Possibility):

Plausible temporal sequence; it is reasonable to suspect drug causation after considering other therapy or the subject/patient's physical condition.

Unlikely:

Does not meet above criteria and current knowledge indicates that a relationship is extremely unlikely.

Definitely Not:

Does not meet above criteria; AE is known to be associated with other therapy or the subject's/patient's physical condition.

Unknown:

Not possible to assign to any other category.

Amended---
OLD>
2.1.5. Clinical Outcome

The following categories are used to assess the clinical outcome of each AE:

- ?*Recovered (with or without residual effects*):

The subject/patient has fully recovered from the AE with or without observable residual effects.

- *Not Yet Recovere*d:

The subject/patient is still being treated for the residual effects of the original AE. This does not include treatment for pre-existing conditions including the indication for the study drug.

- ?*Died Due to This Adverse Event*
- ?*Died, Other Causes*
- ?*Unknown*
- *Surgery/Procedure*

NEW>
June 13, 2003 – Amendment 4
2.1.5. Clinical Outcome
The following categories are used to assess the clinical outcome of each AE:

- ***Unknown:***

The outcome of the event is not known at the time of reporting. Attempt to obtain information for follow-up report.

- ***Patient Recovered***
- ***Patient has Not Yet Recovered:***
  The patient still has symptoms. This can be identified as "Recovering", "Recovered with Sequelae", or "Change in Intensity".
- ***Patient Died Due to Adverse Events or Patient Died due to Other Causes:***

  If the patient died, whether or not the death is attributed to the adverse event. The date and probable cause(s) of death should be reported.

Amended--
OLD>
3.3. Clinical Laboratory Adverse Event

A clinical laboratory abnormality should be reported as an AE only if the conditions are met as defined in Section 2.

NEW>
June 13, 2003 – Amendment 4
3.3 <Letter/Sentence/Paragraph Deleted> Hospitalization or Surgery/Procedure

Amended--
OLD>
3.5. General Physical Examination Findings

At screening, any clinically significant finding should be recorded on the General Medical History CRF. After the signing of the informed consent document, any new clinically significant finding that meets the definition of an AE must be documented as such.

NEW>
June 13, 2003 – Amendment 4
3.5. General Physical Examination Findings
At screening, any clinically significant finding should be recorded on the General Medical History CRF. After the signing of the informed consent document and being formally accepted into the study, any new clinically significant finding that meets the definition of an AE must be documented as such. ***Illnesses or medical events discovered after signing Informed Consent during screening phase but prior to meeting all inclusion/ exclusion criteria should be considered a pre-existing condition, and therefore, not recorded as an AE.***

Amended--
OLD>
4.1. Immediately Reportable Adverse Events

If an AE meets the definition of Serious (see Section 2 of this appendix), it is *immediately reportable*. The investigator should contact Parke-Davis by telephone or fax within 24 hours and should forward a written report to the Clinical/Medical Colleague as soon as possible. In addition, the investigator must complete an AE CRF. If any SAE occurs, the

investigator can withdraw the subject/patient from the study at the investigator's discretion while taking the appropriate follow-up action.

The Parke-Davis Worldwide Adverse Event Reporting System (WAERS) is the database Parke-Davis uses to collect SAE information from all studies. For each immediately reportable AE, a WAERS form must be filled out by the site monitor with the assistance of the investigator/site personnel, describing what is known about the event and its management. A copy of the completed WAERS form must be signed by the investigator and filed at the study site.

The WAERS form is IN ADDITION TO, not instead of, the standard AE CRF. Any discrepancies between the completed standard AE CRF and the WAERS form should be resolved at the time the event is being evaluated and must be resolved prior to submission of the AE CRF to Parke-Davis.

Follow-up reports and an AE CRF must be provided to Parke-Davis within 10 working days of the SAE report.

NEW>
June 13, 2003 – Amendment 4
4.1. Immediately Reportable Adverse Events
If an AE meets the definition of Serious (see Section *1*), it is *immediately reportable*. The investigator should contact ***Pfizer Inc and its affiliates*** by telephone or fax ***and report event*** to the Clinical/Medical Colleague ***immediately***. In addition, the investigator must complete an AE CRF. If any SAE occurs, the investigator can withdraw the subject/patient from the study at the investigator's discretion while taking the appropriate follow-up action.

The Pfizer Inc and its affiliates Worldwide Adverse Event Monitoring System ***(AEM)*** is the database ***Pfizer Inc and its affiliates uses*** to collect SAE information from all studies. For each immediately reportable ***SAE, an AEM*** form must be filled out by the ***Clinical/Medical Colleague*** with the assistance of the investigator/site personnel, describing what is known about the event and its management.
<Letter/Sentence/Paragraph Deleted>

The ***AEM*** form is IN ADDITION TO, not instead of, the standard AE CRF. Any discrepancies between the completed standard AE CRF and the AEM form should be resolved at the time the event is being evaluated and must be resolved prior to submission of the AE CRF to ***Pfizer Inc and its affiliates.***

<Letter/Sentence/Paragraph Deleted>

Amended--
OLD>
4.2. Other Adverse Events

AEs that are not immediately reportable according to the definitions in this appendix will only be recorded on the standard AE CRF. These forms will be collected by the sponsor

after the event is resolved, or if the event is continuing, at approximately 12- to 16-week intervals until after the AE ends or if the AE does not end, until the subject/patient completes the study or the protocol-specified follow-up period.

NEW>
June 13, 2003 – Amendment 4

4.2. Other Adverse Events

AEs that are not immediately reportable according to the definitions in this appendix will be recorded on the standard AE CRF. ***<Letter/Sentence/Paragraph Deleted>***

APPENDIX D
Administrative Procedures for the Reporting of Adverse Events

APPENDIX D
Administrative Procedures for the Reporting of Adverse Events

March 26, 2003 – Amendment 4

INTRODUCTION

The administrative procedures for reporting adverse events described in this appendix are to be followed during the conduct of this protocol.

If you have any questions concerning adverse event reporting, please contact the ***Pfizer Inc and its affiliates*** colleague or representative who is monitoring your site or a ***Pfizer Inc and its affiliates*** Clinical/Medical Colleague whose name, address, and telephone number appears on the cover sheet to this protocol.

TABLE OF CONTENTS

Page

1. STANDARD SAFETY SECTION FOR INCLUSION IN PFIZER-SPONSORED CLINICAL TRIAL PROTOCOLS D4

1.1. Adverse Events D4
1.2. Serious Adverse Events D5
1.3. Abnormal Laboratory Test Results D7
1.4. Abnormal Physical Examination Findings D8
1.5. Discontinuations D9

2. DEFINITIONS D-9
2.1. Attributes of Adverse Events D-10
2.1.1. Treatment-Emergent Signs and Symptoms (TESS) D-10
2.1.2. Serious Adverse Events D-10
2.1.3. Intensity D-10
2.1.4. Relationship to Study Drug—Physician's Assessment D-11
2.1.5. Clinical Outcome D-12

3. CAPTURING ADVERSE EVENTS D-12
3.1. Pre-Existing Condition D-12
3.2. Lack of Efficacy D-12
3.3. <letter/sentence/paragraph deleted> Hospitalization or Surgery/Procedure . D-13
3.4. Death D-13
3.5. General Physical Examination Findings D-13

4. REPORTING TO THE SPONSOR. D-13
4.1. Immediately Reportable Adverse Events D-13
4.2. Other Adverse Events D-14
4.3. Follow-up Period D-14

1. Standard safety section for inclusion in pfizer-sponsored clinical trial protocols

1.1. Adverse Events

All observed or volunteered adverse events, regardless of treatment group or suspected causal relationship to study drug, will be recorded on the adverse event page(s) of the case report form.

Events involving adverse drug reactions, illnesses with onset during the study, or exacerbations of pre-existing illnesses should be recorded. Exacerbation of pre-existing illness, including the disease under study, is defined as a manifestation (sign or symptom) of the illness that indicates a significant increase in the severity of the illness as compared to the severity noted at the start of the trial. It may include worsening or increase in severity of signs or symptoms of the illness, increase in frequency of signs and symptoms of an intermittent illness, or the appearance of a new manifestation/complication. Exacerbation of a pre-existing illness should be considered when a patient/subject requires new or additional concomitant drug or non-drug therapy for the treatment of that illness during the trial. Lack of or insufficient clinical response, benefit, efficacy, therapeutic effect, or pharmacologic action, should not be recorded as an adverse event. The investigator must make the distinction between exacerbation of pre-existing illness and lack of therapeutic efficacy.

In addition, clinically significant changes in physical examination findings and abnormal objective test findings (eg, laboratory, x-ray, ECG) should also be recorded as adverse events. The criteria for determining whether an abnormal objective test finding should be reported as an adverse event are as follows:

1) ***Test result is associated with accompanying symptoms; and/or***
2) ***Test result requires additional diagnostic testing or medical/surgical intervention; and/or***
3) ***Test result leads to a change in study dosing or discontinuation from the study, significant additional concomitant drug treatment or other therapy; and/or***
4) ***Test result leads to any of the outcomes included in the definition of a serious adverse event; and/or***
5) ***Test result is considered to be an adverse event by the investigator or sponsor.***

Merely repeating an abnormal test, in the absence of any of the above conditions, does not meet condition #2 above for reporting as an adverse event.

Any abnormal test result that is determined to be an error does not require reporting as an adverse event, even if it did meet one of the above conditions except for condition #4.

For all adverse events, the investigator must pursue and obtain information adequate both to determine the outcome of the adverse event and to assess whether it meets the

criteria for classification as a serious adverse event requiring immediate notification to Pfizer or its designated representative (see Section 1.2). For all adverse events, sufficient information should be obtained by the investigator to determine the causality of the adverse event (ie, study drug or other illness). The investigator is required to assess causality and indicate that assessment on the CRF. Follow-up of the adverse event, after the date of therapy discontinuation, is required if the adverse event or its sequelae persist. Follow-up is required until the event or its sequelae resolve or stabilize at a level acceptable to the investigator and the Pfizer clinical monitor or his/her designated representative.

1.2. Serious Adverse Events

All serious adverse events (as defined below), regardless of treatment group or suspected relationship to study drug, must be reported immediately by telephone to the named individuals identified within the protocol text.

A serious adverse event is any adverse drug experience occurring at any dose that:

1) *results in death;*
2) *is life-threatening;*
3) *results in inpatient hospitalization or prolongation of existing hospitalization;*
4) *results in a persistent or significant disability/incapacity; or*
5) *results in congenital anomaly/birth defect.*

Important medical events that may not result in death, be life-threatening, or require hospitalization may be considered serious adverse drug experiences when, based upon appropriate medical judgment, they may jeopardize the patient/subject and may require medical or surgical intervention to prevent one of the outcomes listed in this definition. Examples of such medical events include allergic bronchospasm requiring intensive treatment in an emergency room or at home, blood dyscrasias or convulsions that do not result in inpatient hospitalization, or the development of drug dependency or drug abuse.

Regardless of the above criteria, any additional adverse experiences which Pfizer personnel or an investigator considers serious should be immediately reported to Pfizer and included in the Corporate adverse events database system.

A life-threatening adverse event is any adverse drug experience that places the patient/subject at immediate risk of death from the reaction as it occurred; ie, it does not include a reaction that, had it occurred in a more severe form, might have caused death.

Initial hospitalization is defined as any inpatient admission (even if less than 24 hours). For chronic or long-term inpatients, inpatient admission also includes transfer within the hospital to an acute/intensive care inpatient unit (eg, from the psychiatric wing to a medical floor, from a medical floor to the coronary care unit, from the neurological floor to the tuberculosis unit).

1) *Inpatient admission in the absence of a precipitating, treatment-emergent, clinical adverse event may meet criteria for "seriousness" but is not an adverse experience and thus is not subject to immediate reporting to Pfizer. For example:*

   i. *Admission for treatment of a pre-existing condition not associated with the development of a new adverse event or with a worsening of the pre-existing condition (eg, for work-up of persistent pretreatment lab abnormality)*
   ii. *Social admission (eg, subject has no place to sleep)*
   iii. *Administrative admission (eg, for yearly physical exam)*
   iv. *Protocol-specified admission during a clinical trial (eg, for a procedure required by the study protocol)*
   v. *Optional admission not associated with a precipitating clinical adverse event (eg, for elective cosmetic surgery)*

   However, if a hospitalization for an unknown event occurs, it should be considered as a serious adverse event.

2) *Inpatient admission does not include the following:*

   i. *Emergency Room/Accident and Emergency/Casualty Department visits*
   ii. *Outpatient/same-day/ambulatory procedures*
   iii. *Observation/short-stay units*
   iv. *Rehabilitation facilities*
   v. *Hospice facilities*
   vi. *Respite care (eg, caregiver relief)*
   vii. *Skilled nursing facilities*
   viii. *Nursing homes*
   ix. *Custodial care facilities*
   x. *Clinical research/Phase I units*

Prolongation of hospitalization is defined as any extension of an inpatient hospitalization beyond the stay anticipated/required in relation to the original reason for the initial admission, as determined by the investigator or treating physician. For protocol-specified hospitalizations in clinical trials, prolongation is defined as any extension beyond the length of stay described in the protocol. Prolongation in the absence of a precipitating, treatment-emergent, clinical adverse event (ie, not associated with the development of a new adverse event or worsening of a pre-existing condition) may meet criteria for "seriousness" but is not an adverse experience and thus is not subject to immediate reporting to Pfizer.

Preplanned treatments or surgical procedures should be noted in the baseline documentation for the entire protocol and/or for the individual patient/subject.

Disability is a substantial disruption of a person's ability to conduct normal life functions.

Any serious adverse event or death must be reported immediately independent of the circumstances or suspected cause if it occurs or comes to the attention of the

investigator at any time during the study through the last follow-up visit required by the protocol or 30 days after the last administration of study drug, whichever comes later. Any serious adverse event occurring at any other time after completion of the study must be promptly reported if a causal relationship to study drug is suspected. The only exception to these reporting requirements are serious adverse events that occur during a prerandomization/
washout run-in period, during which either placebo alone is administered, or no active study drug or no protocol-specified background drug is administered.

For all serious adverse events, the investigator is obligated to pursue and provide information as requested by the Pfizer clinical monitor or designated representative in addition to that on the case report form. In general, this will include a description of the adverse event in sufficient detail to allow for a complete medical assessment of the case and independent determination of possible causality. Information on other possible causes of the event, including concomitant medications and illnesses must be provided. The investigator's assessment of causality must also be provided. If causality is unknown and the investigator does not know whether or not study drug caused the event, then it should be attributed to study drug. If the investigator's causality assessment is "unknown but not related to study drug," this should be clearly documented on study records. In the case of a subject death, a summary of available autopsy findings must be submitted as soon as possible to Pfizer or its designated representative. The investigator should ensure that information reported immediately by telephone or other means and information entered in the case report form are accurate and consistent.

1.3. Abnormal Laboratory Test Results

The results of all laboratory tests required by the protocol will be recorded in the subject's case report form. All clinically important abnormal laboratory tests occurring during the study will be repeated at appropriate intervals until they return either to baseline or to a level deemed acceptable by the investigator and the Pfizer clinical monitor (or his/her designated representative), or until a diagnosis that explains them is made. The criteria for determining whether an abnormal objective test finding should be reported as an adverse event are as follows:

1) *Test result is associated with accompanying symptoms, and/or*
2) *Test result requires additional diagnostic testing or medical/surgical intervention, and/or*
3) *Test result leads to a change in study dosing or discontinuation from the study, significant additional concomitant drug treatment or other therapy, and/or*
4) *Test result leads to any of the outcomes included in the definition of a serious adverse event, and/or*
5) *Test result is considered to be an adverse event by the investigator or sponsor.*

Merely repeating an abnormal test, in the absence of any of the above conditions, does not meet condition #2 above for reporting as an adverse event.

Any abnormal test result that is determined to be an error does not require reporting as an adverse event, even if it did meet one of the above conditions except for condition #4.

1.4. Abnormal Physical Examination Findings

Clinically significant changes, in the judgment of the investigator, in physical examination findings (abnormalities) will be recorded as adverse events.

1.5. Discontinuations

The reason for a subject discontinuing from the study will be recorded in the case report form. A discontinuation occurs when an enrolled subject ceases participation in the study, regardless of the circumstances, prior to completion of the protocol. The investigator must determine the primary reason for discontinuation. Withdrawal due to adverse event should be distinguished from withdrawal due to insufficient response according to the definition of adverse event noted earlier. A discontinuation must be reported immediately to the Pfizer clinical monitor or his/her designated representative if it is due to a serious adverse event. The final evaluation required by the protocol will be performed at the time of study discontinuation. The investigator will record the reason for study discontinuation, provide or arrange for appropriate follow-up (if required) for such subjects, and document the course of the subject's condition.

March 26, 2003 – Amendment 4

2. DEFINITIONS

Pre-Existing Condition

A pre-existing condition is one that is present at the start of study treatment.

Baseline

Defined by the ***protocol as applicable to the study.***

<Letter/Sentence/Paragraph Deleted>

Related Adverse Event

An AE where there is a reasonable possibility that the experience may have been caused by drug (Unknown is also considered related).

<Letter/Sentence/Paragraph Deleted>

<Letter/Sentence/Paragraph Deleted>

Unexpected Adverse Event

Any adverse event, the specificity or severity of which is not consistent with the current Investigator's Brochure. For example, under this definition, hepatic necrosis would be unexpected (by virtue of greater severity) if the Investigator's Brochure only referred to elevated hepatic enzymes or hepatitis.

Similarly, cerebral thromboembolism and cerebral vasculitis would be unexpected (by virtue of greater specificity) if the Investigator's Brochure only listed cerebral vascular accidents. "Unexpected," as used in this definition, refers to an AE that has not been previously observed (eg, included in the Investigator's Brochure) rather than from the perspective of such experience not being anticipated from the pharmacological properties of the pharmaceutical product.

<Letter/Sentence/Paragraph Deleted>

Treatment-Emergent Signs and Symptoms (TESS)

Any AE that was not evident during baseline as defined by the study protocol, or that increases in intensity or frequency, or changes in character during treatment.

Treatment-Emergent Signs and Symptoms (TESS)

Any AE that was not evident during baseline as defined by the study protocol, or that increases in intensity or frequency, or changes in character during treatment.

Posttreatment Adverse Event

Any AE that occurs after study treatment is discontinued. Posttreatment follow-up and posttreatment adverse events of interest to the study will be defined per protocol.

Lack of Efficacy

A worsening of the disease being studied or lack of desired effect of the study drug (not reported as an AE if defined as an efficacy parameter in the protocol).

2.1. Attributes of Adverse Events

2.1.1. Treatment-Emergent Signs and Symptoms (TESS)

Any condition/diagnosis that meets the definition of a TESS event is captured as such on the AE CRF.

March 26, 2003 – Amendment 4

2.1.2. Serious Adverse Events

All SAEs, as defined in Section *1.2*, are immediately reportable to ***Pfizer Inc. and its affiliates. <Letter/Sentence/Paragraph Deleted>***

If there is an exception to the SAE definition, it is described in the protocol.

2.1.3. Intensity

Appendix D
Version 2, 24 September 2002

The following criteria are used to assess the intensity of each AE:

• *Mild*: The subject/patient is aware of the sign or symptom, but finds it easily tolerated.
• *Moderate*: The subject/patient has discomfort enough to cause interference with or change in usual activities.
• *Severe:* The subject/patient is incapacitated and unable to work or participate in many or all usual activities.

Note: For oncology studies, in lieu of these criteria, standardized coding criteria may be used as defined in the protocol.

<u>March 26, 2003 – Amendment 4</u>
2.1.4. Relationship to Study Drug—Physician's Assessment
There are 6 categories for the physician's assessment of the causal relationship between study drug and an AE:

Physician's Assessment	*Causal Relationship Reported to Regulatory Agencies*
Definitely	***Yes***
Probably	***Yes***
Possibly	***Yes***
Unlikely	***No***
Definitely Not	***No***
Unknown[a]	***Yes (ie, interpreted as "possibly" related)***

[a] ***Insufficient information or indeterminable, too***

The following criteria are to be used to assess the causal relationship between an AE and study drug:

Definite:

Follows a reasonable temporal sequence from administration of the drug or from drug levels established in body fluids or tissues; AE improves or disappears after stopping or reducing the dosage (dechallenge); AE reappears with rechallenge.

or

Is known to be associated with the drug or class of compounds and cannot be explained by other therapy or subject/patient's physical condition.

Probable:

Follows a reasonable temporal sequence from drug administration; AE improves or disappears after stopping or reducing the dosage (dechallenge) and rechallenge was not attempted; AE cannot be explained by other therapy or the subject/patient's physical condition.

Possible (Reasonable Possibility):

Plausible temporal sequence; it is reasonable to suspect drug causation after considering other therapy or the subject/patient's physical condition.

Unlikely:

Does not meet above criteria and current knowledge indicates that a relationship is extremely unlikely.

Definitely Not:

Does not meet above criteria; AE is known to be associated with other therapy or the subject's/patient's physical condition.

Unknown:

Not possible to assign to any other category.

March 26, 2003 – Amendment 4
2.1.5. Clinical Outcome
The following categories are used to assess the clinical outcome of each AE:

- ***Unknown:***

  The outcome of the event is not known at the time of reporting. Attempt to obtain information for follow-up report.
- ***Patient Recovered***
- ***Patient has Not Yet Recovered:***
  The patient still has symptoms. This can be identified as "Recovering", "Recovered with Sequelae", or "Change in Intensity".
- ***Patient Died Due to Adverse Events or Patient Died due to Other Causes:***

  If the patient died, whether or not the death is attributed to the adverse event. The date and probable cause(s) of death should be reported.

3. CAPTURING ADVERSE EVENTS

3.1. Pre-Existing Condition
A pre-existing condition should be reported as an AE if the frequency, intensity, or the character of the condition worsens during study treatment.

3.2. Lack of Efficacy

Signs or symptoms defined in the protocol as lack of efficacy or collected as efficacy parameters should not be reported as AEs.

March 26, 2003 – Amendment 4
3.3. *<Letter/Sentence/Paragraph Deleted>* Clinical Laboratory Adverse Event

A clinical laboratory abnormality should be reported as an AE only if the conditions are met as defined in Section 2.

Any AE that results in hospitalization (ie, subject/patient admitted—not just an emergency room visit) should be reported as an SAE unless specifically instructed otherwise in the protocol. Any condition/diagnosis responsible for surgery/procedure should be reported as an AE if it meets the criteria for an AE. The surgery/procedure itself will be reported as a Clinical Outcome of the underlying event.

Events that prolong any hospitalization are reported as SAEs.

3.4. Death

The cause of death should be reported as an AE. Death should not be reported as an AE, but as a Clinical Outcome. The only exception is "Sudden Death" when the cause of death is unknown, which is reported as an AE with death as the Clinical Outcome.

March 26, 2003 – Amendment 4
3.5. General Physical Examination Findings

At screening, any clinically significant finding should be recorded on the General Medical History CRF. After the signing of the informed consent document and being formally accepted into the study, any new clinically significant finding that meets the definition of an AE must be documented as such. ***Illnesses or medical events discovered after signing Informed Consent during screening phase but prior to meeting all inclusion/ exclusion criteria should be considered a pre-existing condition, and therefore, not recorded as an AE.***

4. REPORTING TO THE SPONSOR

All AEs that occur at any time during the study including the posttreatment period as defined in the protocol, are to be reported in the subject's/patient's CRFs.

<u>March 26, 2003 – Amendment 4</u>

4.1. Immediately Reportable Adverse Events

If an AE meets the definition of Serious (see Section ***1***), it is *immediately reportable*. The investigator should contact ***Pfizer Inc and its affiliates*** by telephone or fax ***and report event*** to the Clinical/Medical Colleague ***immediately***. In addition, the investigator must complete an AE CRF. If any SAE occurs, the investigator can withdraw the subject/patient from the study at the investigator's discretion while taking the appropriate follow-up action.

The Pfizer Inc and its affiliates Worldwide Adverse Event Monitoring System ***(AEM)*** is the database ***Pfizer Inc and its affiliates uses*** to collect SAE information from all studies. For each immediately reportable ***SAE, an AEM*** form must be filled out by the ***Clinical/Medical Colleague*** with the assistance of the investigator/site personnel, describing what is known about the event and its management.
<Letter/Sentence/Paragraph Deleted>

The ***AEM*** form is IN ADDITION TO, not instead of, the standard AE CRF. Any discrepancies between the completed standard AE CRF and the AEM form should be resolved at the time the event is being evaluated and must be resolved prior to submission of the AE CRF to ***Pfizer Inc and its affiliates***.

<Letter/Sentence/Paragraph Deleted>

<u>March 26, 2003 – Amendment 4</u>

4.2. Other Adverse Events

AEs that are not immediately reportable according to the definitions in this appendix will be recorded on the standard AE CRF. ***<Letter/Sentence/Paragraph Deleted>***

4.3. Follow-up Period

For SAEs, the subject/patient must remain under observation until the SAE has subsided or stabilized and all serious pathological values and findings have returned to normal or stabilized.

Follow-up information will not be collected for "not yet recovered" or continuing nonserious AEs unless time frames are specifically written in the protocol.
